# From GWAS to drug screening: repurposing antipsychotics for glioblastoma

**DOI:** 10.1186/s12967-021-03209-2

**Published:** 2022-02-04

**Authors:** Wei-Zhi Lin, Yen-Chun Liu, Meng-Chang Lee, Chi-Tun Tang, Gwo-Jang Wu, Yu-Tien Chang, Chi-Ming Chu, Chia-Yang Shiau

**Affiliations:** 1 Graduate Institute of Life Sciences, National Defense Medical Center, No.161, Sec. 6, Minquan E. Rd., Neihu Dist., Taipei City, 11490 Taiwan; 2 School of Medicine, National Defense Medical Center, No.161, Sec. 6, Minquan E. Rd., Neihu Dist., Taipei City, 11490 Taiwan; 3 School of Public Health, National Defense Medical Center, No.161, Sec. 6, Minquan E. Rd., Neihu Dist., Taipei City, 11490 Taiwan; 4 Graduate Institute of Medical Sciences, National Defense Medical Center, Taipei, Taiwan; 5 Department of Neurological Surgery, Tri-Service General Hospital, No. 325, Sec. 2, Chenggong Rd., Neihu District, Taipei, 11490 Taiwan; 6 Department of Obstetrics and Gynecology, Tri-Service General Hospital, No. 325, Sec. 2, Chenggong Rd., Neihu District, Taipei, 11490 Taiwan; 7 Fidelity Regulation Therapeutics Inc., 161, Sec. 6, Minquan E. Rd., Neihu Dist., Taipei City, 11490 Taiwan

**Keywords:** Glioblastoma, In-silico screening, Drug repositioning, Antipsychotics, Antitumor

## Abstract

**Background:**

Glioblastoma is currently an incurable cancer. Genome-wide association studies have demonstrated that 41 genetic variants are associated with glioblastoma and may provide an option for drug development.

**Methods:**

We investigated FDA-approved antipsychotics for their potential treatment of glioblastoma based on genome-wide association studies data using a ‘pathway/gene-set analysis’ approach.

**Results:**

The *in-silico* screening led to the discovery of 12 candidate drugs. DepMap portal revealed that 42 glioma cell lines show higher sensitivities to 12 candidate drugs than to Temozolomide, the current standard treatment for glioblastoma.

**Conclusion:**

In particular, cell lines showed significantly higher sensitivities to Norcyclobenzaprine and Protriptyline which were predicted to bind targets to disrupt a certain molecular function such as DNA repair, response to hormones, or DNA-templated transcription, and may lead to an effect on survival-related pathways including cell cycle arrest, response to ER stress, glucose transport, and regulation of autophagy. However, it is recommended that their mechanism of action and efficacy are further determined.

**Supplementary Information:**

The online version contains supplementary material available at 10.1186/s12967-021-03209-2.

## Background

Glioblastoma multiforme (GBM) classified by the WHO as a grade IV glioma is the most malignant and lethal tumor to occur in the brain with rapid de novo progression and limited survival rate [1, 2]. The usual conventional therapies for GBM are surgical resection, radiation therapy, and chemotherapy. Concurrent radio- and chemotherapy will usually be enforced after surgery in case the cancer cells have invaded distal tissue or infiltrated the parenchyma. Currently, Temozolomide (TMZ) is the standard drug of choice used for the primary management against GBM. TMZ is a prodrug that is spontaneously hydrolyzed into an active form, 3-methyl-(triazen-1-yl) imidazole-4-carboxamide which alkylates DNA and triggers the death of tumor cells [3]. However, the median survival time (overall survival) of GBM patients is only extended a few months with TMZ treatment [4], and resistance to TMZ usually quickly develops through DNA repair mediated by methyl guanine methyl transferase, base excision repair or alkylpurine-DNA-N-glycosylase [3, 5]. Although an FDA-approved anti-angiogenic therapy (bevacizumab) with a combination of TMZ has been applied in GBM management, it did not result in significant improvement in overall survival but only prolonged progression-free survival [6, 7]. Therefore, a therapeutic approach for GBM still falls far short of medical needs.

Genome-wide association studies (GWASs) investigate the association between genetic variants and traits of interest, for example, the association between single nucleotide polymorphisms (SNPs) and diseases [8, 9]. In rare cases, SNPs located in the promoter or the well-known regulatory elements can be easily correlated to the regulation of genes. In most of cases (~ 88%), the SNPs of interest are located in intergenic or intronic regions, and are often associated with traits of interest with unknown reason [10]. Currently, four GWASs have reported that 41 risky SNPs are associated with GBM [11–14], only three of them are located in the untranslated regions or miRNA which is known to be implicated in oncogenesis [15–17]. Rs78378222 disrupts the polyadenylation of TP53 resulting in impaired mRNA processing and its reduced expression [15]. Rs10069690, providing an alternative splicing site and leading to a splice isoform of Telomerase reverse transcriptase [16], was found to have the most significant P value associated with GBM. Rs11558961 affects the secondary structure of Glial fibrillary acidic protein mRNA, which promotes the binding of miR-139, and thus decreases the susceptibility to chemotherapy [17]. The roles of other SNPs in GBM remain unclear to date.

Drug discovery and development are an expensive and highly time-consuming undertaking. It usually costs billions of dollars and takes decades to bring a compound to clinical application. Furthermore, newly developed drugs do not always confer a superior clinical benefit [18]. Scientists and pharmaceutical industries face growing demand for new drugs to fit unmet medical needs, and are under pressure to increase R&D productivity with limited resources [19]. Drug repositioning (or drug repurposing) is a process that resort to the approved drugs for new indications or answer of unmet medical needs. Clinically approved drugs have been proven to be safe, and the dosage range and formulation are already well studied. Taking advantage of abundant information about clinically approved drugs, drug development by drug repositioning may have a lower risk of failure, cost less and require less time to complete preclinical and phase I/II clinical trials [20, 21]. Furthermore, the efficacy for unidentified targets of approved drugs within other diseases remains worthwhile pursuit.

Over the past decades, GWASs have uncovered a lot of genetic variants which may provide targets for drug repositioning. Lau and So summarized several approaches for drug repositioning using GWAS data [22]. The ‘candidate gene approach’ is the most straightforward method that maps the top risky SNPs to their related genes by functional annotation tools or eQTL, and then queries these genes in the gene-drug database. This approach is a clear-cut strategy with low computational cost, but meets difficulties such as limited druggable genes [23], uncertain effect size of risky SNPs [24], and the complexity of proper annotation of risky SNPs. This approach may also miss multi-target drugs which are considered to be more effective than single target drugs [25]. The ‘pathway/gene-set analysis’ organizes multiple function or biologically-related genes and further studies their perturbational expression under interruption of drugs. Although the ‘pathway/gene-set analysis’ seems to be able to overcome the defects of the ‘candidate gene approach’, its challenge lies in the complexity of identifying the drug-mediated pathways or gene sets, because the mechanisms of many drugs are not fully understood. The computational tools Gene2drug [26] and Drug Set Enrichment Analysis (DSEA) [27] provide an opportunity to overcome the complexity of identifying drug-mediated pathways. Gene2drug analyses the genes perturbed by drug treatments and annotates those genes to biological/functional pathways; DSEA reversely annotates queried drugs to the pathways.

Previous review indicate that patients receiving drugs acting on the brain to sedate psychosis have a lower incidence of cancers than the general population [28]. Thus, antipsychotics have been considered as potential candidate agents against GBM [29–31]. This work aimed to discover whether there are current drugs that may be beneficial for GBM therapy using GWAS data via ‘pathway/gene-set analysis’. Gene2drug, Gene Ontology (GO) Resource [32] and the Kyoto Encyclopedia of Genes and Genomes (KEGG) [33] were used to assist in *in-silico* screening. The antitumor activities of the candidate drugs and TMZ were extracted from DepMap for which data were identified by PRISM viability assay on 42 glioma cell lines [34].

## Materials and methods

### Data source

The risky SNPs of GBM (Trait NO.: EFO_0000519) and the annotated risk genes were collected from the *GWAS Catalog, EMBL-EBI* [35]. Gene2drug (https://gene2drug.tigem.it/) is a state-of-the-art online tool and easily accessible technique applying the Gene Expression Profiles to Pathway Expression Profiles (gep2pep) algorithm [26] with Connectivity Map (CMap) database Molecular Signatures Database (MSigDB). The gene ontology of the annotated genes recoded by Gene Ontology Resource (GO) [32] or KEGG [33]were selected. The sensitivity of glioma cells to candidate drugs or TMZ were sorted from PRISM Repurposing Secondary Screen 19Q4 [34]. The gene mutation profiles (Mutation Public 21Q1) were extracted from DepMap portal (https://depmap.org/portal/). The gene expression profiles were downloaded from Cancer Cell Line Encyclopedia (CCLE_expression 21Q1) [36].

### Gene to drug analysis

Candidate compounds were suggested by Gene2drug with the imported biological pathways in which the annotated genes were involved [26]. Briefly, the biological pathways of query genes were selected. A list of compounds will be generated by each pathway which suggests that the compounds in the list may interrupt the pathway based on data from MSigDB. Then, a merge process which selected compounds exist in all the lists and are ranked by P-value decides the output of Gene2drug.

Nine annotated genes were found to be involved in a certain biological process. The significant compounds which had a P value lower than 1E-2 were included. The compounds co-existing in more than four lists were selected (six lists at most with candidate drugs included, a median number, four, was set as cut-off), and then chosen within FDA approved drugs with known central nervous activities based on the treatment note of PRISM assay. The co-existence of compounds among the lists was analyzed with tool Venn Diagrams (Van der Peer Lab) and outputted in text format.

### Prediction of pathways in which the candidate drugs are involved

The pathways which may be mediated by candidate drugs were predicted by using DSEA [27]. The downstream pathway shared by candidate drugs and the pathway based similarity were generated at the same time. The enrichment scores (E-score) from −1 to + 1 refer to down-regulation or up-regulation by drug treatments.

### Validating the antitumor activity of candidate drugs

The sensitivity of candidate drugs and TMZ was extracted from DepMap for which data were identified by PRISM viability assay on more than 40 glioma cell lines. Briefly, cell lines with a barcode integrated in the genome were exposed to compounds for five days. Then, the mRNA was isolated from cells, amplified and detected. The viability of cells was calculated based on the level of barcode abundance and the sensitivity of cells to compounds was generated by comparing the treatment group and the control group [34]. The process sorted data from PRISM was performed by a python script (https://github.com/LinWZ-tw/g2d-prism).

### Gene expression profile

The expression profiles of cell lines were further analyzed with data downloaded from the CCLE via DepMap portal. The cluster and heatmaps of genes were generated by using ClustVis [37].

### Prediction of drug target

The targets of candidate drugs were predicted by a web-based platform, GalaxySagittarius, which combines ligand similarity-based and receptor structure-based approaches with AUC up to 0.8 [38]. Briefly, a protein target database is generated by similarity-based screening, then the target protein is ranked by protein–ligand docking. The predictions were run with a search model of similarity combined prediction and 3D structure compound obtained from ZINC: ZINC000002040609 (Norcyclobenzaprine) and ZINC000001530764 (Protriptyline). The 3D binding poses and binding structures were downloaded from the web server; the 2D binding structures were generated by using BIOVIA Discovery Studio 2020. The gene ontologies of interest were analyzed by GO Ontology Resource (http://geneontology.org/) [32].

### Limitations

For *in-silico* screening, we collected GBM-associated SNPs and their mapping genes from the GWAS Catalog which applies Ensembl mapping pipeline for genome annotation. Although the risky SNPs are likely associated with their mapping gene, the possibility that risky SNPs have an effect on distal genes via chromatin looping structures may be omitted [10]. The *in-silico* screening conducted by Gene2drug was limited by the size of MSigDB while it is one of the largest and most popular databases so far [26, 39]. Validating work was relied on PRISM data which may contain divergent results in replicate tests.

## Results

### *In-silico* screening of potential drugs

**Fig. 1:**
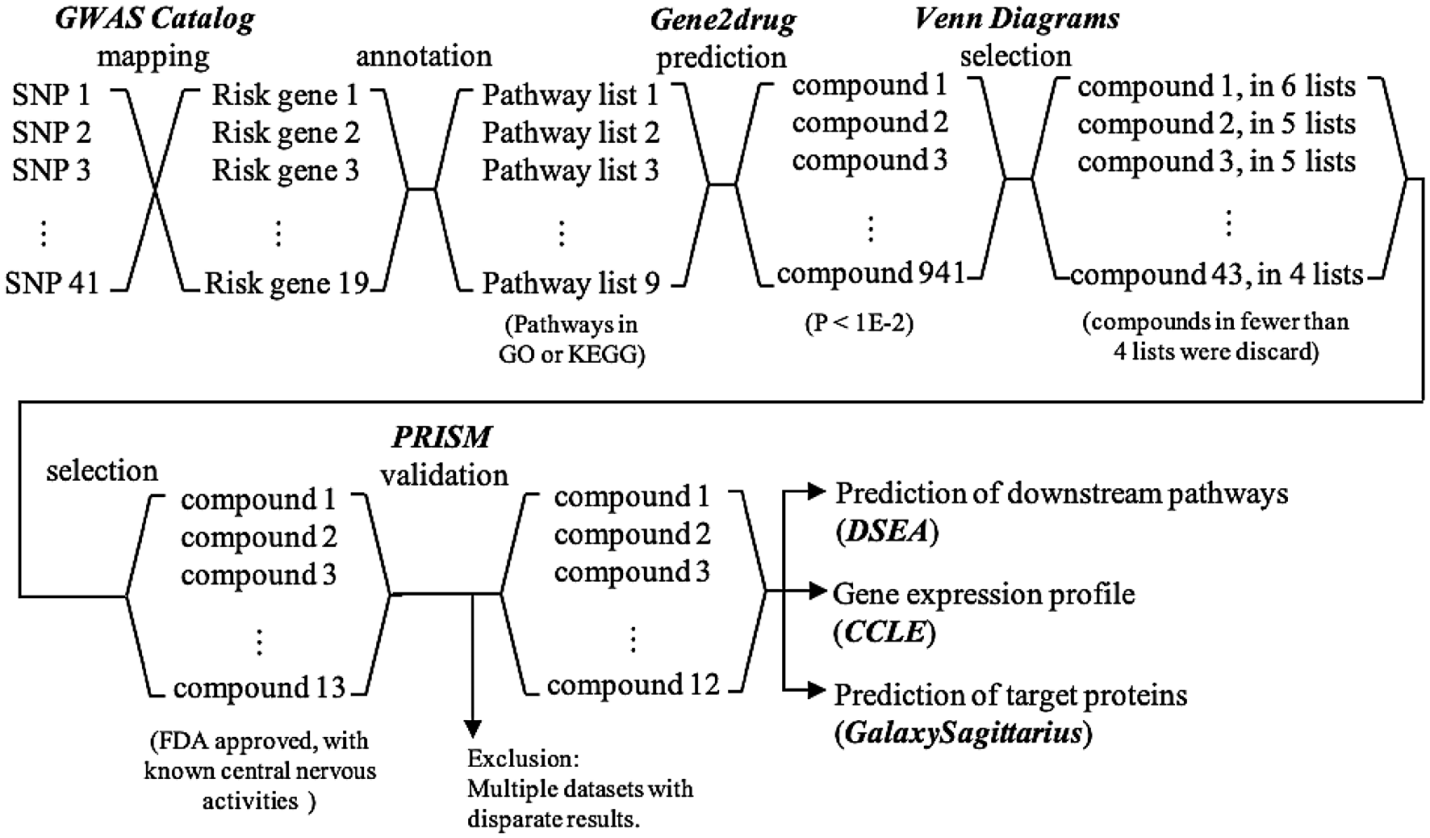
Experimental design and flow chart for *in-silico* screening

Four studies from the *GWAS Catalog* were used [11–14], which include 41 risky SNPs and 15 annotated genes (Additional file 1: Table S1). Only 9 of the annotated genes were found to be involved in biological processes or pathways collected in GO or KEGG (Additional file 1: Table S2). Then, lists of candidate compounds from each annotated gene were generated from Gene2drug [26]. Further analysis of compounds showed that one compound co-existed in six lists, eight compounds co-existed in five lists and 20 compounds co-existed in four lists (Table S3), no compounds could be found in more than six lists, and compounds found in fewer than four lists were discarded. Given that the aim was drug repositioning for GBM therapy, compounds had been approved by FDA for safety, and compounds known to affect the central nervous system were selected. A total 12 FDA-approved drugs were suggested (Table 1, Additional file 1: Figure S1). The protocol design and overall flowchart is shown (Fig. 1).

**Table 1:** *In-silico* screening led to 12 candidate drugs

Drug	Indication	Publication in PubMed		Clinical Trial
		*Brain cancer*	*Other Cancers*	
Norcyclobenzaprine	Depression	Yes	NA	NA
Protriptyline	Depression	NA	Prostate cancer	NA
Iobenguane	Neuroblastoma	Yes	NA	Pheochromocytoma and paraganglioma: FDA approved (AZEDRA)
Haloperidol	Schizophrenia Tourette's disorder	Yes	Pancreatic cancer	NA
Alimemazine	Sedative	NA	Colon cancer	NA
Nortriptyline	Depression	NA	Bladder tumor, multiple myeloma, osteosarcoma and prostate cancer	Relapsed Small Cell Carcinoma: Phase I (NCT02881125)
Melatonin	Sleep cycle support	Yes	Pan-cancer	30 completed clinical trials in multiple oncological indications
Trifluoperazine	Schizophrenia	Yes	Breast cancer, colon cancer, lung cancer, lymphoma, multiple myeloma ovarian cancer and pancreatic cancer	Doxorubicin resistance cancer: Phase I/II
Perphenazine	Schizophrenia Nausea Vomiting	Yes	Breast cancer, colon cancer, endometrial cancer, leukemia, melanoma and pancreatic cancer	NA
Spiperone	Schizophrenia	Yes	Gastric cancer	NA
Imipramine	Depression	Yes	Colon cancer, small cell lung cancer	Breast Cancer: Early phase I (NCT03122444)
Levomepromazine	Psychosis Schizophrenia Bipolar disorder Nausea Insomnia	NA	Breast cancer and leukemia	NA

### Prediction of pathways in which the candidate drugs are involved

The downstream pathways shared by candidate drugs were predicted by DSEA (see Methods). A list of 327 downstream pathways shared by 12 candidate drugs were collected (Additional file 1: Table S4). The much commonly shared pathways of significance (lowest P value) were ranked by DSEA utility (Additional file 1: Figure S2A). Among them, several categories of pathway which may lead to cell death are summarized, including cell cycle arrest, response to ER stress, glucose transport, and regulation of autophagy (Table 2).

**Table 2:** Categories of pathways predicted to involve the candidate drugs

*Category*	*Pathway name*	E-score*	*P value*
Mitotic cell cycle	Mitotic nuclear envelope disassembly	− 0.73	5.63E−06
	Mitotic cell cycle	− 0.70	1.61E−05
	G1/S transition of mitotic cell cycle	− 0.59	5.60 E−04
	Mitotic metaphase plate congression	− 0.52	3.03 E−03
	Mitotic sister chromatid segregation	− 0.51	4.24 E−03
	Regulation of ubiquitin-protein ligase activity involved in mitotic cell cycle	− 0.51	4.33 E−03
	Negative regulation of mitotic cell cycle	0.50	4.76 E−03
	Negative regulation of ubiquitin-protein ligase activity involved in mitotic cell cycle	− 0.47	1.04 E−02
	Positive regulation of ubiquitin-protein ligase activity involved in mitotic cell cycle	− 0.45	1.57 E−02
	Mitotic cell cycle arrest	0.44	1.97 E−02
ER stress	Endoplasmic reticulum unfolded protein response	0.70	1.49 E−05
	Response to endoplasmic reticulum stress	0.66	6.22 E−05
	Intrinsic apoptotic signaling pathway in response to endoplasmic reticulum stress	0.56	1.07 E−03
Glucose transport	Regulation of glucose transport	− 0.70	1.79 E−05
	Glucose transport	− 0.51	4.59 E−03
	Negative regulation of glucose import	0.49	7.22 E−03
	Cellular response to glucose starvation	0.46	1.37 E−02
Autophagy	Positive regulation of autophagy	0.54	2.01 E−03
	Autophagy	0.52	3.08 E−03
	Regulation of autophagy	0.47	1.15 E−02

^*^ Escore: Enrichment score from − 1 to + 1, refer to down/up-regulation

### The sensitivities of glioma cells to candidate drugs

**Fig. 2:**
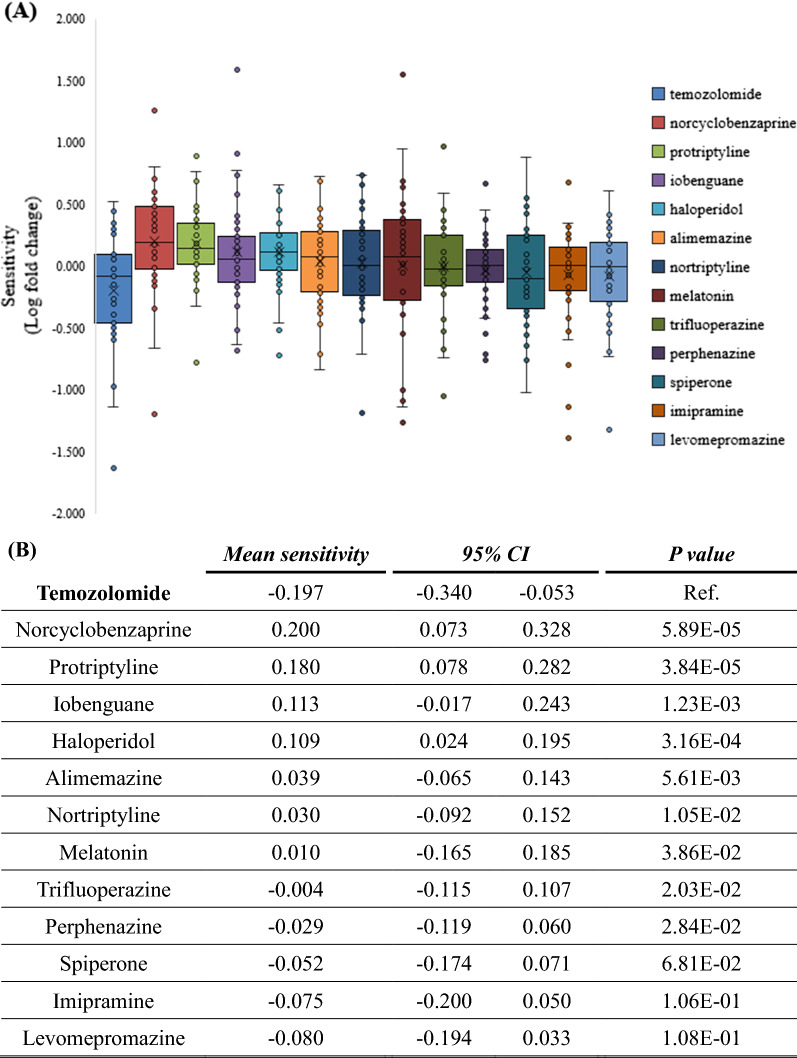

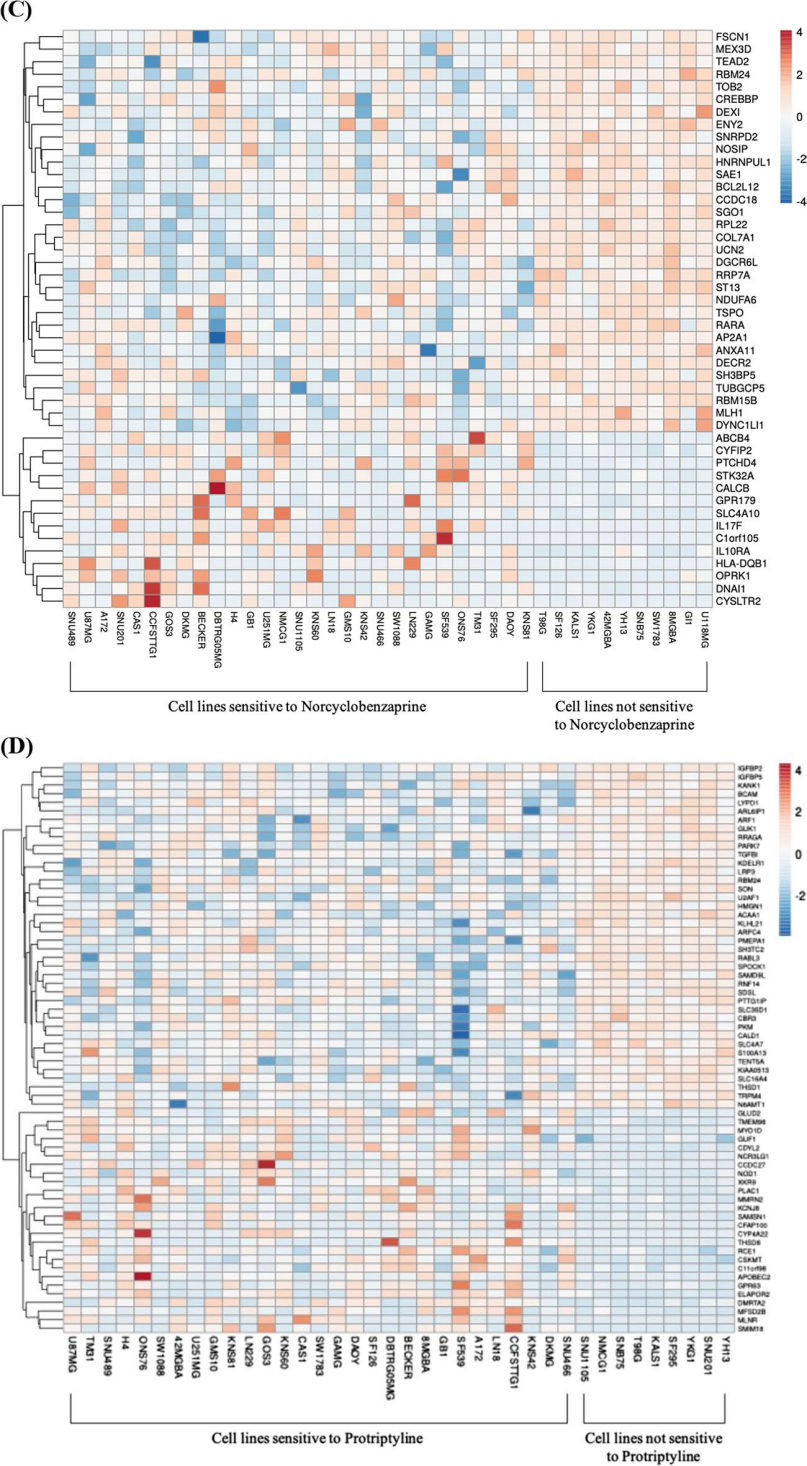
Sensitivity of glioma cells to TMZ and candidate drugs. **A** The sensitivities of glioma cells to candidate drugs and TMZ ranking by mean sensitivity. (Ranking by mean sensitivity. × : mean value. CI: Confidence Interval) (**B**) Cells show significant higher sensitivities to two of candidate, Norcyclobenzaprine and Protriptyline (*P* = 5.89E−05 and 3.84E−05 respectively). **C**, **D** Heatmap of gene show significantly different expression in sensitive and non-sensitive cells to Norcyclobenzaprine and Protriptyline, respectively. The order of cell line follows the sensitivities of cells to Norcyclobenzaprine and Protriptyline from highest (left) to lowest (right)

Among the candidate drugs output from screening protocol, Chlorprothixene was excluded because two datasets of Chlorprothixene were found in which glioma cells show disparate responses. The sensitivities of 42 glioma cells to the rest 12 candidate drugs were extracted from PRISM (Additional file 1: Table S5). The sensitivities to all the candidate drugs were higher than that to TMZ based on mean sensitivity among cell types (Fig. 2A, B).

In particular, Norcyclobenzaprine and Protriptyline were interesting in that their mean sensitivities were significantly higher than that to TMZ (*P* < 1 E−4) (Fig. 2A, B). The sensitivities to Protriptyline have no significant correlation with well-known oncological genes such as expression level of EGFR or mutations in TP53 and PTEN (Additional file 1: Figure S3A–C and G). The sensitivities to Norcyclobenzaprine were correlated with mutations in TP53 (0.017 by Mann–Whitney U test) but were not correlated with PTEN or expression level of EGFR (Additional file 1: Figure S3 D–F and G). It suggests that these two candidate drugs may have potential application on tumors with a wide spectrum of genetic backgrounds, while the mutation in TP53 should be avoid when Norcyclobenzaprine is applied.

To investigate the difference between sensitive and not sensitive cells, genes showing a significantly (*P* < 1E−3) different level of expression were selected. A group of genes with a consistent pattern in cells not sensitive to Norcyclobenzaprine was disclosed (Fig. 2C). Another group of genes showed a similar pattern in cells not sensitive to Protriptyline (Fig. 2D). Subjects with similar expression level in those groups of gene may not respond to Norcyclobenzaprine and Protriptyline.

### Prediction of drug target

Several proteins were predicted by GalaxySagittarius to be targets of candidate drugs, the top ten from each prediction were selected (Additional file 1: Table S6). The top ten for Norcyclobenzaprine were: Poly(ADP-Ribose) Polymerase 1 (PARP1), Poly(ADP-Ribose) Polymerase 2 (PARP2), Orosomucoid 2 (ORM2), Retinol Binding Protein 1 (RBP1), SET domain containing lysine methyltransferase 7 (SETD7), Estrogen Related Receptor Gamma (ESRRG), Progesterone Receptor (PGR), Estrogen Receptor 1 (ESR1), Nuclear Receptor Coactivator 2 (NCOA2), Retinoic Acid Receptor Beta (RARB). The top ten for Protriptyline were: RBP1, PGR, NCOA2, PARP2, PARP1, Retinoid X Receptor Alpha (RXRA), RARB, Choline Kinase Alpha (CHKA), Retinoic Acid Receptor Gamma (RARG), ORM2. The binding poses of candidate drugs and their interaction with predicted proteins are shown (Additional file 1: Figure S4). The targets of both Norcyclobenzaprine and Protriptyline have similar gene ontology which are correlated with DNA-templated transcription, RNA synthesis, response to hormone, retinoic acid biosynthetic process, etc. (Table 3).

**Table 3:** The ontology of predicted target genes

***Predicted targets***	***Norcyclobenzaprine***			
	***GO biological process complete***	***Over/under***	***Fold enrichment***	***FDR***
PARP1 PARP2 ORM2 RBP1 SETD7 ESRRG PGR ESR1 NCOA2 RARB	Positive regulation of RNA biosynthetic process (GO:1902680)	+	7.78	3.68 E−03
	Positive regulation of transcription DNA-templated (GO:0045893)	+	7.78	4.39 E−03
	Positive regulation of RNA metabolic process (GO:0051254)	+	7.15	4.66 E−03
	Positive regulation of macromolecule biosynthetic process (GO:0010557)	+	6.8	4.69 E−03
	Transcription initiation from RNA polymerase II promoter (GO:0006367)	+	41.69	4.84 E−03
	Positive regulation of macromolecule metabolic process (GO:0010604)	+	4.5	4.92 E−03
	Positive regulation of transcription by RNA polymerase II (GO:0045944)	+	9.15	4.93 E−03
	Response to steroid hormone (GO:0048545)	+	27.79	4.94 E−03
	Intracellular receptor signaling pathway (GO:0030522)	+	32	4.97 E−03
	Positive regulation of biosynthetic process (GO:0009891)	+	6.32	5.03 E−03
	Epithelial cell development (GO:0002064)	+	31.37	5.11 E−03
	transcription by RNA polymerase II (GO:0006366)	+	20.74	5.11 E−03
	positive regulation of cellular biosynthetic process (GO:0031328)	+	6.44	5.14 E−03
	DNA-templated transcription initiation (GO:0006352)	+	32.5	5.24 E−03
	DNA ADP-ribosylation (GO:0030592)	+	> 100	5.27 E−03
	Peptidyl-serine ADP-ribosylation (GO:0018312)	+	> 100	5.44 E−03
	Positive regulation of nucleic acid-templated transcription (GO:1903508)	+	7.78	5.49 E−03
	Response to hormone (GO:0009725)	+	11.61	5.74 E−03
	Positive regulation of metabolic process (GO:0009893)	+	4.15	5.76 E−03
	Positive regulation of nucleobase-containing compound metabolic process (GO:0045935)	+	6.53	5.93 E−03
	Hormone-mediated signaling pathway (GO:0009755)	+	48.37	6.34 E−03
	Cellular response to hormone stimulus (GO:0032870)	+	17.97	6.92 E−03
	Mammary gland branching involved in pregnancy (GO:0060745)	+	> 100	7.72 E−03
	RNA biosynthetic process (GO:0032774)	+	14.59	7.95 E−03
	Retinoic acid biosynthetic process (GO:0002138)	+	> 100	8.02 E−03
	Steroid hormone mediated signaling pathway (GO:0043401)	+	66.01	8.16 E−03
	Nucleic acid-templated transcription (GO:0097659)	+	14.92	8.33 E−03
	Transcription, DNA-templated (GO:0006351)	+	14.95	8.61 E−03
	Diterpenoid biosynthetic process (GO:0016102)	+	> 100	8.69 E−03
	Positive regulation of nitrogen compound metabolic process (GO:0051173)	+	4.57	8.70 E−03
	Protein poly-ADP-ribosylation (GO:0070212)	+	> 100	8.98 E−03
	Vitamin A metabolic process (GO:0006776)	+	> 100	9.95 E−03

***Predicted targets***	***Protriptyline***			
	***GO biological process complete***	***Over/under***	***Fold enrichment***	***FDR***
RBP1 PGR NCOA2 PARP2 PARP1 RXRA RARB CHKA RARG ORM2	Cellular response to hormone stimulus (GO:0032870)	+	17.97	3.46 E−03
	Retinoic acid receptor signaling pathway (GO:0048384)	+	> 100	4.74 E−03
	Hormone-mediated signaling pathway (GO:0009755)	+	48.37	6.34 E−03
	Transcription by RNA polymerase II (GO:0006366)	+	20.74	8.18 E−03
	Positive regulation of transcription by RNA polymerase II (GO:0045944)	+	9.15	8.21 E−03
	DNA ADP-ribosylation (GO:0030592)	+	> 100	8.29 E−03
	Transcription initiation from RNA polymerase II promoter (GO:0006367)	+	41.69	8.47 E−03
	Epithelial cell development (GO:0002064)	+	31.37	8.51 E−03
	Intracellular receptor signaling pathway (GO:0030522)	+	32	8.59 E−03
	DNA-templated transcription, initiation (GO:0006352)	+	32.5	8.91 E−03
	Peptidyl-serine ADP-ribosylation (GO:0018312)	+	> 100	9.66 E−03

### Direct screening protocol based on PRISM assay data

We built another screening protocol which directly ranks drugs according to PRISM assay data (Additional file 1: Figure S5A). In the field of neurology, there are 124 FDA-approved medications. Replicate tests were found in some of drugs result in a total of 140 treatments including TMZ as a control were employed. Glioma cells show higher mean sensitivities to 107 treatments (76.98%) than that to TMZ, but only 23 (16.55%) out of them show significantly higher sensitivities than TMZ (*P* < 1E−3). Among the candidate drugs with sensitivities higher than TMZ, the standard deviations were ranging from 0.255 to 0.805 (Additional file 1: Figure S5B). The top ten candidate drugs were Amitriptyline, Tranylcypromine, Mianserin, Triflupromazine, Doxylamine, Protriptyline, Metixene, Citalopram, Benserazide and Acepromazine (Additional file 1: Figure S5C-D). Moreover, nine of top ten candidate drugs were predicted to bind to PGR and eight were predicted to bind to Androgen Receptor (AR) (Additional file 1: Figure S5E). Both of PGR and AR have been reported to play important roles in glioma [40–42].

## Discussion

A total 12 FDA-approved drugs were suggested as drugs of possible interest for GBM in our *in-silico* screening: Norcyclobenzaprine, Protriptyline, Iobenguane, Haloperidol, Alimemazine, Nortriptyline, Melatonin, Trifluoperazine, Perphenazine, Spiperone, Imipramine and Levomepromazine. Protriptyline, Nortriptyline and Imipramine are used for treatment of depression and classified as tricyclic antidepressants (TCAs). Norcyclobenzaprine is one of the major metabolites of Cyclobenzaprine which is usually used for muscle spasms, while both Norcyclobenzaprine and Cyclobenzaprine can act as TCAs to block serotonin receptors. A previous case–control study indicated that long-term use of TCAs may be associated with reduced risk of glioma [43]. TCAs are known to down-regulate β-adrenergic receptors which are involved in carcinogenesis and are considered as drug targets [44]. Melatonin is commonly used for sleep disorders. Haloperidol, Trifluoperazine, Perphenazine, Spiperone and Levomepromazine are clinically used for psychosis, especially schizophrenia or bipolar disorder. Alimemazine could be used as a sedative. Perphenazine and Levomepromazine are also used to control nausea and vomiting. Alimemazine, Trifluoperazine, Perphenazine and Levomepromazine are structurally similar compounds based on a phenothiazine. One of the candidate drugs, Iobenguane, is already an FDA-approved drug for low-grade glioma, indicating the credibility of our *in-silico* screening protocol.

Among these candidate drugs, Norcyclobenzaprine, Iobenguane, Haloperidol, Melatonin, Trifluoperazine, Perphenazine, Spiperone and Imipramine have been studied for their effects on brain tumors. For example, Norcyclobenzaprine has been reported to inhibit the proliferation of GBM cells but with IC_50_ values higher than 10 µM [45]. Iobenguane has been developed and approved by the FDA as a clinical agent for treating pheochromocytoma and paraganglioma [46]. The candidate drugs identified in our study have not only been studied for use on GBM but also many types of human malignancies [47–75] and many clinical trials have been completed [76–78] or are ongoing. Especially, there are 30 completed trials and 15 ongoing trials with intervention with Melatonin. Even though the antitumor activities of the candidate drugs have been reported, most studies exclusively report the antitumor activity of antipsychotic on a few representative cell lines. PRISM provided an overview of the sensitivity among 42 glioma cell lines to queried drugs. The cell lines which are outliers in sensitivity could be further studied as a foundation for selection of participants in future clinical trials. For example, Norcyclobenzaprine did not show significant antitumor activity on certain cell lines [45]; however, the overview of sensitivity on 42 glioma cell lines indicates promising potential. Protriptyline, Alimemazine, Nortriptyline and Levomepromazine have been studied for their effects on many types of human malignancies, but have not yet been reported to have been tested on brain cancers (Table 1).

Most of candidate drugs are similar on correlation matrix according to GO biological process except iobenguane and melatonin (Additional file 1: Figure S2B). But the candidate drugs are not very similar on correlation matrix according to sensitivity (Additional file 1: Figure S2C). Iobenguane, alimemazine, perphenazine, spiperone and imipramine show significant difference when comparing the correlation matrix according to GO biological processes and that according to sensitivities on glioma cells (Additional file 1: Figure S2D).

We further predicted the targets of candidate drugs by GalaxySagittarius. The binding poses of candidate drugs and predicted proteins show that the tricyclic structure of Norcyclobenzaprine and Protriptyline contributes to π-interactions in the inner part of the binding pocket and the amine group at the ‘tail’ structure contributes to H-bonds with residues close to the mouth of the binding pocket (Additional file 1: Figure S4). Compounds with similar structures were found to have anti-tumor activity. For example, Triflupromazine which has a similar structure to trifluoromethyl-phenothiazine in the ‘head’ and a tertiary amine in the ‘tail’ shows potential against GBM (Additional file 1: Figure S5C).

While most of the pathways participated in by each risk gene did not overlap (Additional file 1: Table S2), the candidate compounds suggested by Gene2drug co-existed in multiple lists predicted from different genes (Additional file 1: Table S3). It is common that a compound may have multiple off-target mechanisms. We hypothesize that the candidate compounds which disrupt several pathways associated with GBM may have greater potential than those with a single pathway associated with GBM. We further merged the compounds suggested by each input of a gene through ranking the candidate compounds by the number of lists in which they co-existed. Then, we selected a median number as cut-off criteria, and the candidate drugs co-existing in at least four lists of pathways were employed in further analysis. Using TMZ as a control, the sensitivity of cells to all the candidate drugs selected by this protocol was higher than that to TMZ. On the other hand, the top ten candidate drugs which were directly ranked according to mean sensitivity have lower *P*-value than the 12 candidate drugs correlated with multiple disease-risk genes (Fig. 2B, Additional file 1: S5D). We assume that a drug which only interrupts a few key survival-related genes still may trigger cell death and could lead to apparent antineoplastic outcome. For example, Triflupromazine was predicted to disrupt pathways which only correlate with TP53, but Triflupromazine shows an antineoplastic activity significantly higher than TMZ (*P* = 2.63E−05, Additional file 1: Figure S5D). While screening protocol directly based on PRISM cancer cell sensitivity data without prior search of inherent biological pathway and risk genes resulted in a parallel consistent list of candidate drugs, though more, it provided less information for further drug development other than sensitivity. Our current development regimen not only aims to work on cancer disease but also for other diseases of which potential drug targets and pathways are also involved. Furthermore, for some indications for which cell- or animal-models are difficult to construct, the approach that selects candidates correlated with several disease-related genes may reduce the number of candidates to a small figure, reducing the resources required for bench work.

Based the target prediction of candidate drugs by GalaxySagittarius and disrupted pathway predicted by DSEA, we suspect that Norcyclobenzaprine and Protriptyline may bind to targets of the “shell”, such as PARP1, PARP2 or PGR, to interrupt a certain molecular function, for example, DNA-template transcription, response to hormone stimulation, etc. Then the influence of treatment is further extended to survival-related “core” pathways including cell cycle arrest, response to ER stress, glucose transport, and regulation of autophagy. However, the link between “shell” and “core” remains to be further investigated.

## Conclusion

The current study presents a screening protocol following selection of candidates using Gene2drug. Selection from candidate compounds which correlate with multiple disease-risk genes or variants may reduce the number of candidates and decrease the burden of bench work to validate their therapeutic efficacy.

Our *in-silico* screening led to ten antipsychotics which show anti-tumor activity which is higher than TMZ in 42 cell lines. In particular, Norcyclobenzaprine and Protriptyline show significant potential against GBM; they are predicted to bind targets such as PARP1, PARP2, PRG, RBP1 to disrupt DNA repair pathways, respond to hormone and DNA-templated transcription and the retinoic acid signaling pathway, further effect survival-related pathways including cell cycle arrest, response to ER stress, glucose transport, and regulation of autophagy. Of these, the activity of Protriptyline against GBM has not yet been reported. The mechanism of action and therapeutic efficacy of Norcyclobenzaprine and Protriptyline are worth pursuing further.

## Supplementary Information

**Additional file 1.** Additional figures and tables.

## Data Availability

The datasets generated and/or analysed during the current study are available in the GWAS Catalog, https://www.ebi.ac.uk/gwas/; PRISM, https://www.theprismlab.org/; DepMap, https://depmap.org/portal/.

## Abbreviations

AR: Androgen receptor
CHKA: Choline Kinase Alpha
CMap: Connectivity Map
DSEA: Drug Set Enrichment Analysis
ESR1: Estrogen Receptor 1
ESRRG: Estrogen Related Receptor Gamma
gep2pep: Gene Expression Profiles to Pathway Expression Profiles
GO: Gene Ontology
GBM: Glioblastoma multiforme
GWASs: GWASs: Genome-wide association studies
KEGG: Kyoto Encyclopedia of Genes and Genomes
MSigDB: Molecular Signatures Database
NCOA2: Nuclear Receptor Coactivator 2
ORM2: Orosomucoid 2
PARP1: Poly(ADP-Ribose) Polymerase 1
PARP2: Poly(ADP-Ribose) Polymerase 2
PGR: Progesterone Receptor
RARB: Retinoic Acid Receptor Beta
RARG: Retinoic Acid Receptor Gamma
RXRA: Retinoid X Receptor Alpha
RBP1: Retinol Binding Protein 1
SETD7: SET domain containing lysine methyltransferase 7
SNPs: Single nucleotide polymorphisms
TMZ: Temozolomide
TCAs: Tricyclic antidepressants
