# Supplementary material for: From GWAS to drug screening: repurposing antipsychotics for glioblastoma
Source: J Transl Med. 2022 Feb 4;20:70. doi: 10.1186/s12967-021-03209-2 (PMC8815269; doi:10.1186/s12967-021-03209-2)
Supplement: Supplementary file 1 — Additional file 1. Additional figures and tables. [file 12967_2021_3209_MOESM1_ESM.docx]

# Supplement


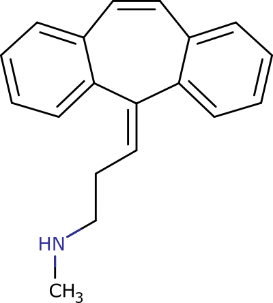

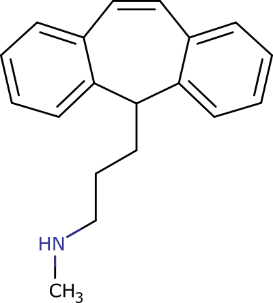

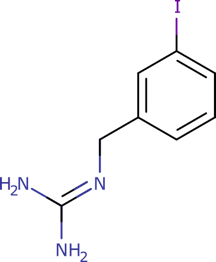

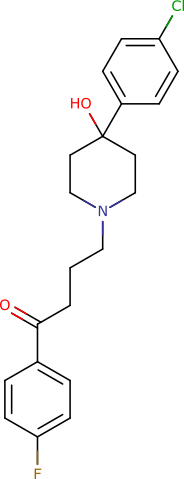


(A) Norcyclobenzaprine (B) Protriptyline (C) Iobenguane (D) Haloperidol


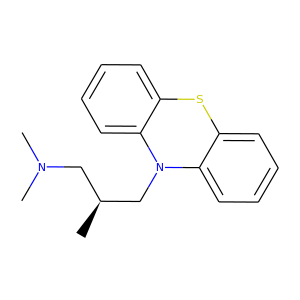

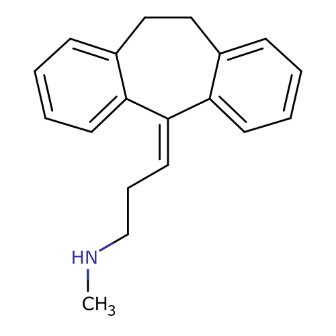

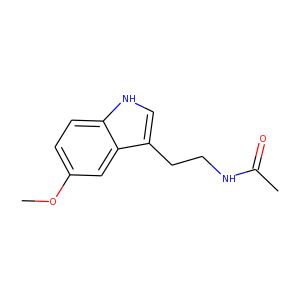

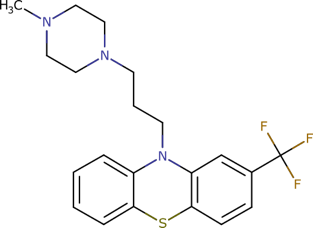


(E) Alimemazine (F) Nortriptyline (G) Melatonin (H) Trifluoperazine


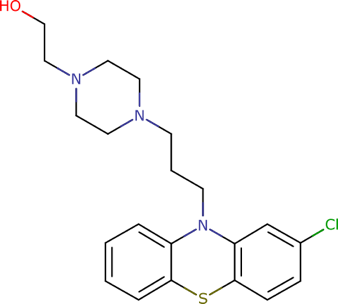

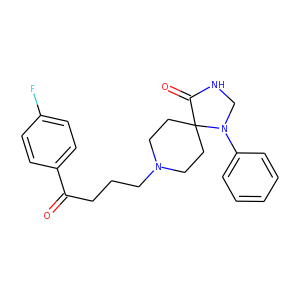

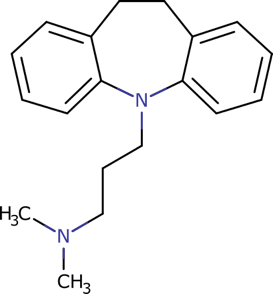

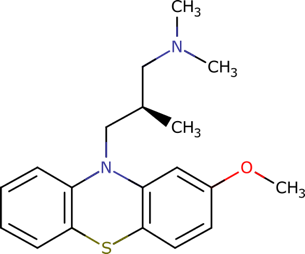


(I) Perphenazine (J) Spiperone (K) Imipramine (L) Levomepromazine

Figure S1. Structure of candidate drugs from ZINC.

(A)


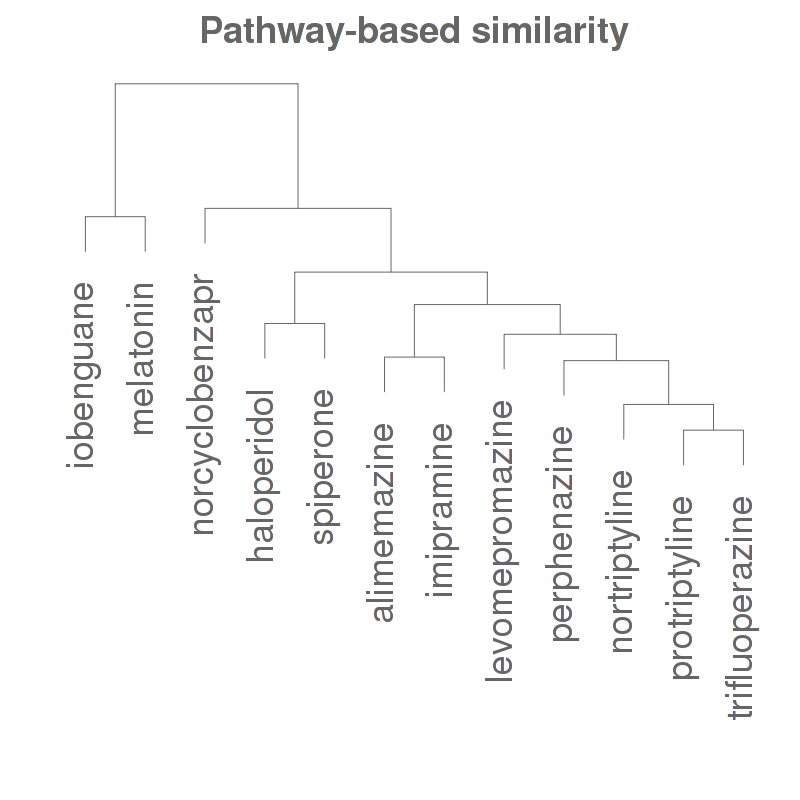


(B) (C)


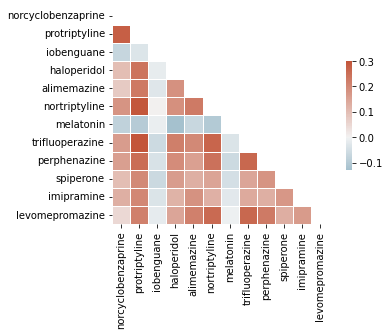

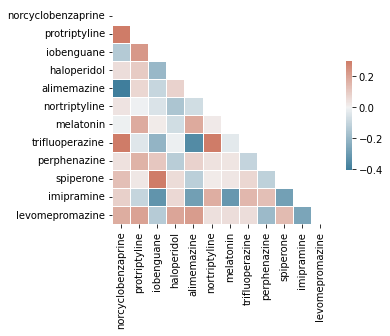


(D)

|  | norcyclobenzaprine | protriptyline | iobenguane | haloperidol | alimemazine | nortriptyline | melatonin | trifluoperazine | perphenazine | spiperone | imipramine | levomepromazine |
| --- | --- | --- | --- | --- | --- | --- | --- | --- | --- | --- | --- | --- |
| Wilcoxon sign rank | .477 | .790 | .026 | .534 | .033 | .062 | .091 | .075 | .013 | .026 | .033 | .062 |

Figure S2. Pathway-based similarity of candidate drugs

(A) The pathway-based similarity generated by DSEA. (B) The correlation matrix according to GO biological process and (C) that according to the sensitivity on glioma cells. (D) The statistical summary compares matrix according to biological process and that according to sensitivity on glioma cells. The Wilcoxon sign rank was conducted by SPSS (version 22.0).

**(A)**

**(B) (C)**

**
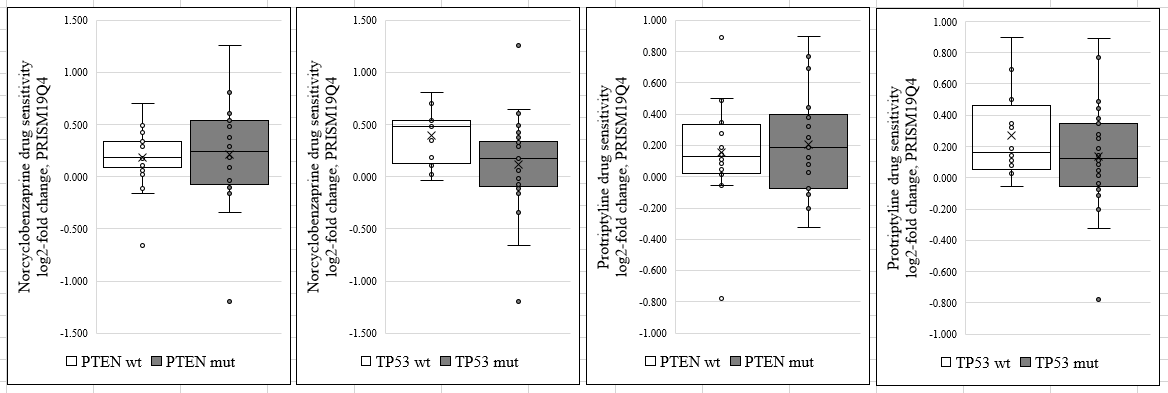

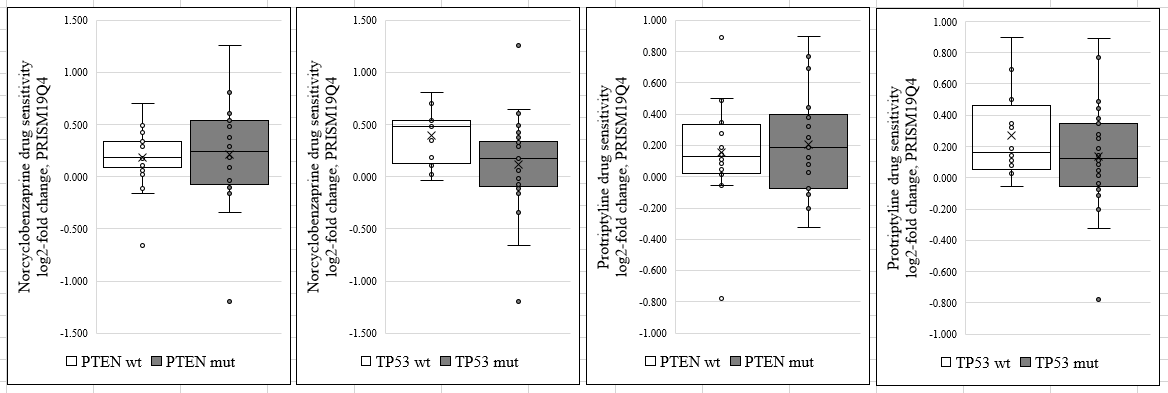
**

**(D)**

**(E) (F)**

**
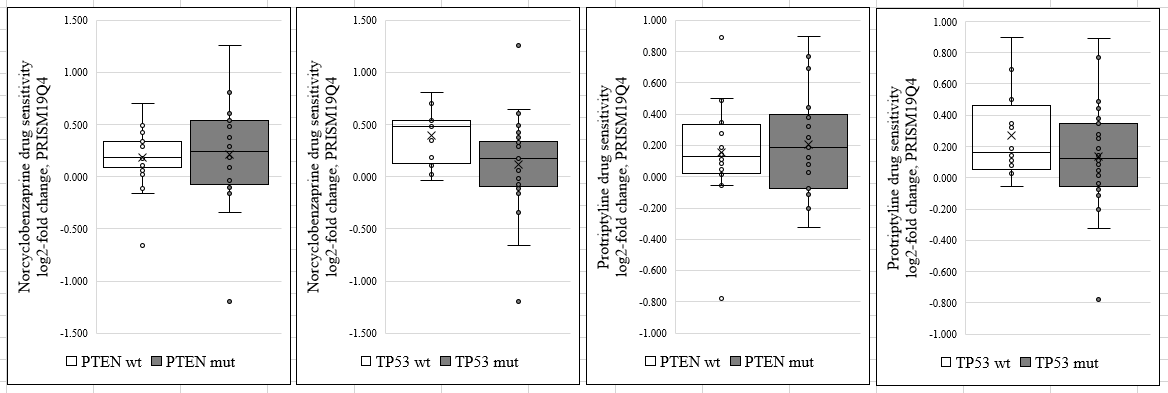

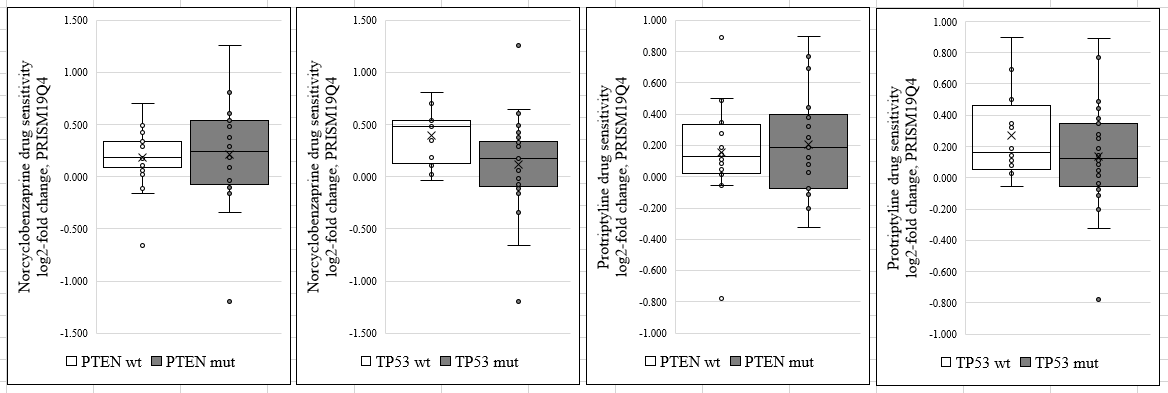
**

**(G)**

|  | ***Sensitivity to Norcyclobenzaprine*** | | |
| --- | --- | --- | --- |
|  | ***Spearman Correlation coefficient*** | ***P-value***  (linear regression) | ***P-value***  (Mann-Whitney U test) |
| EGFR expression | -0.035 | 7.26E-01 | - |
| PTEN mutation | - | - | .718 |
| TP53 mutation | - | - | .017 |
|  | ***Sensitivity to Protriptyline*** | | |
| EGFR expression | 0.181 | 5.73E-01 | - |
| PTEN mutation | - | - | .901 |
| TP53 mutation | - | - | .343 |

Figure S3. Correlation between the sensitivity of glioma cells to candidate drugs and the expression/mutation of oncological genes.

(A) Correlation plots showing the sensitivity to Norcyclobenzaprine *vs* EGFR expression, (B) *vs* PTEN mutation, (C) *vs* TP53 mutation; (D) the sensitivity to Protriptyline *vs* EGFR expression, (E) *vs* PTEN mutation, (F) *vs* TP53 mutation. (G) Summary of statistical correlation. The Spearman and P-value by linear regression were obtained from the DepMap portal. The Mann-Whitney U test was conducted by SPSS (version 22.0).

(A) (B)


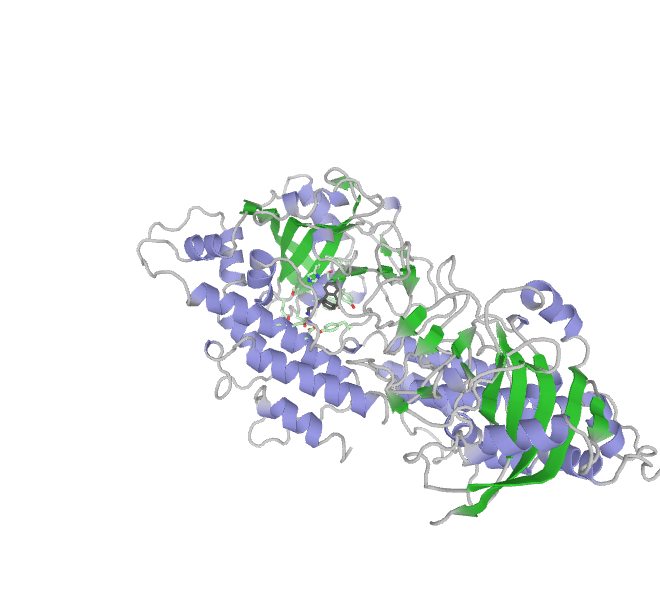

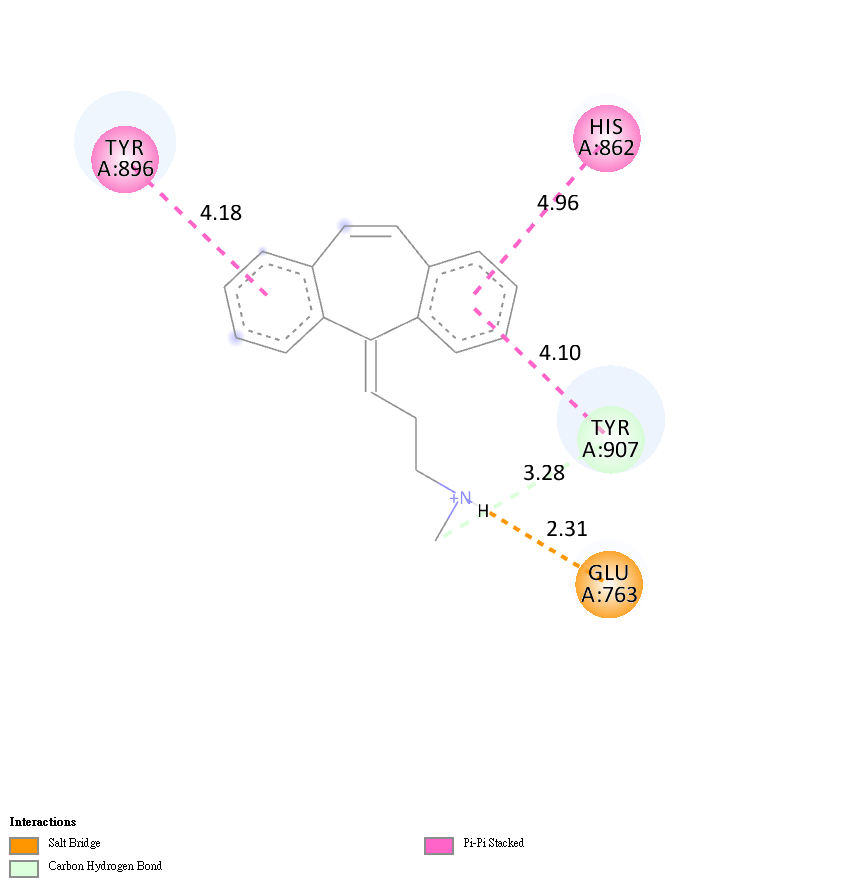

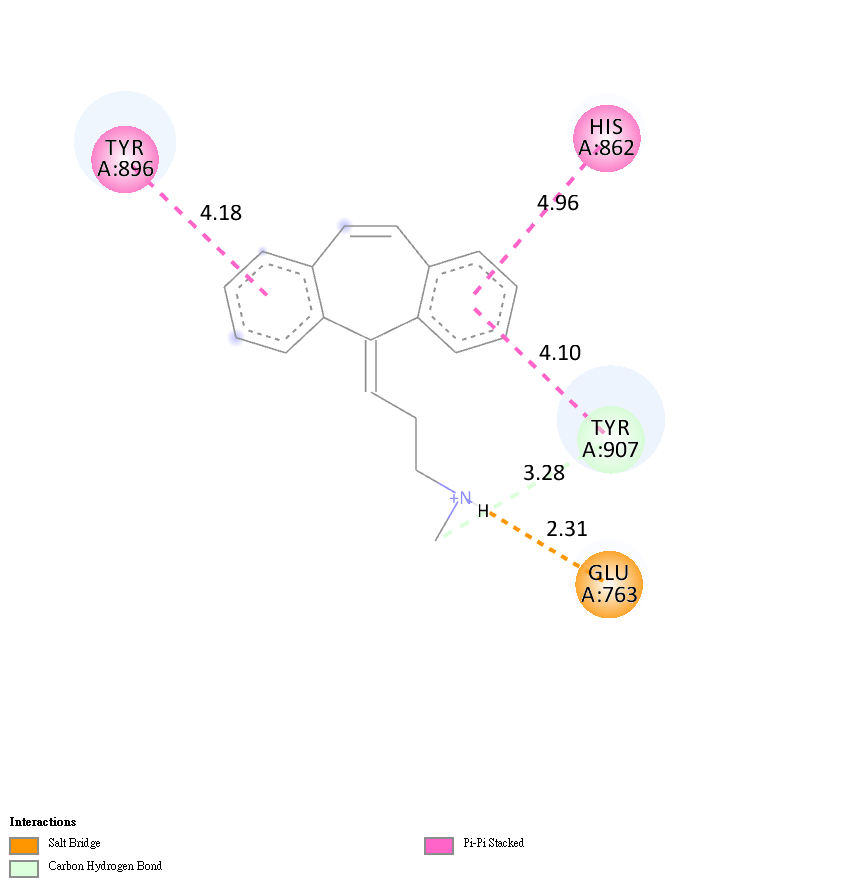

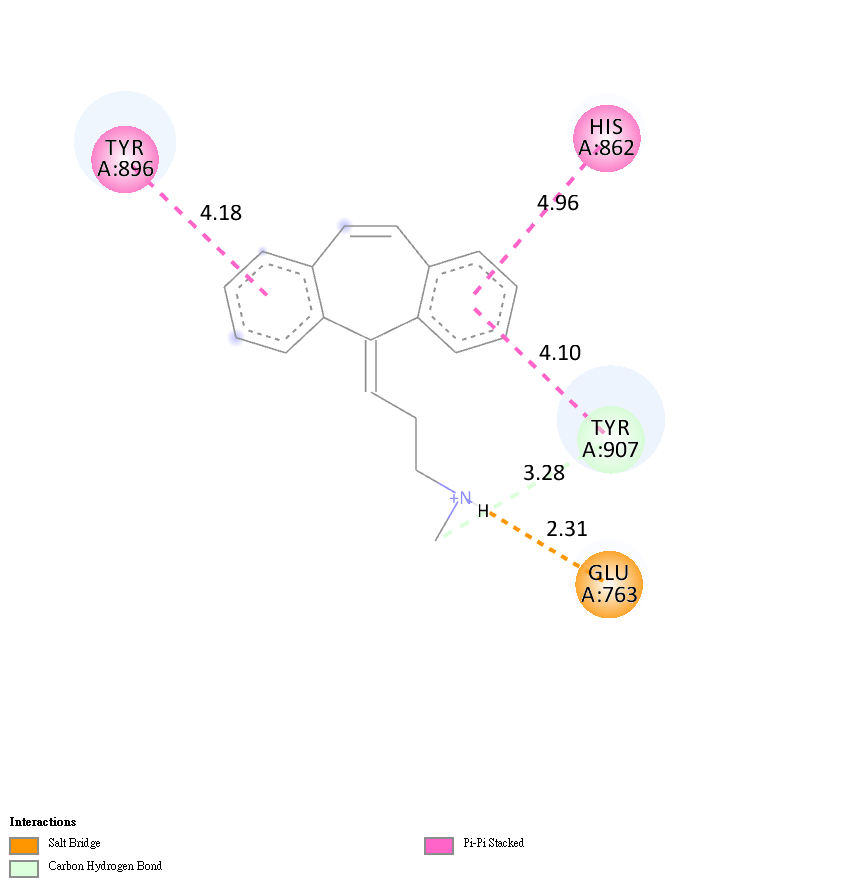

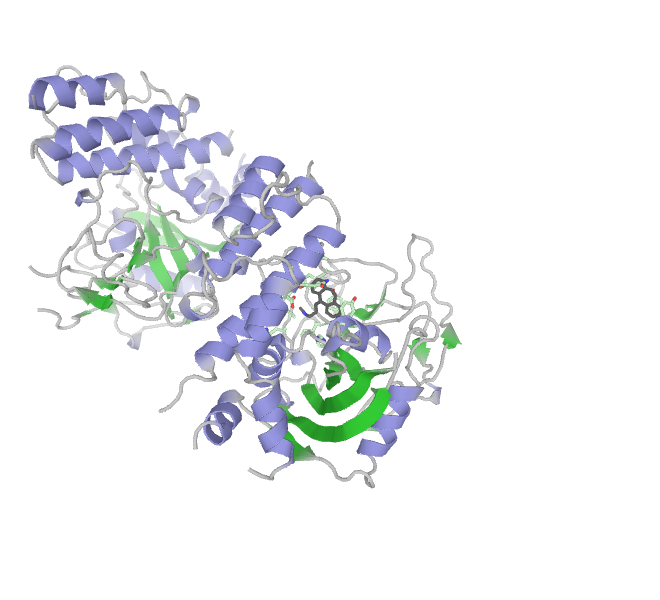

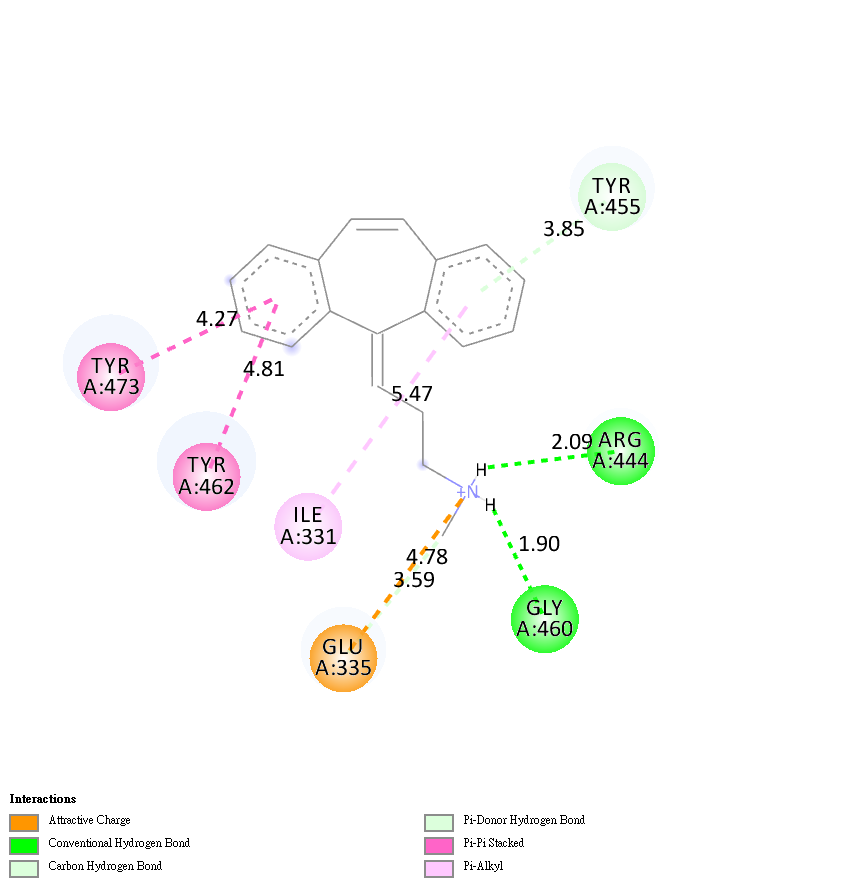

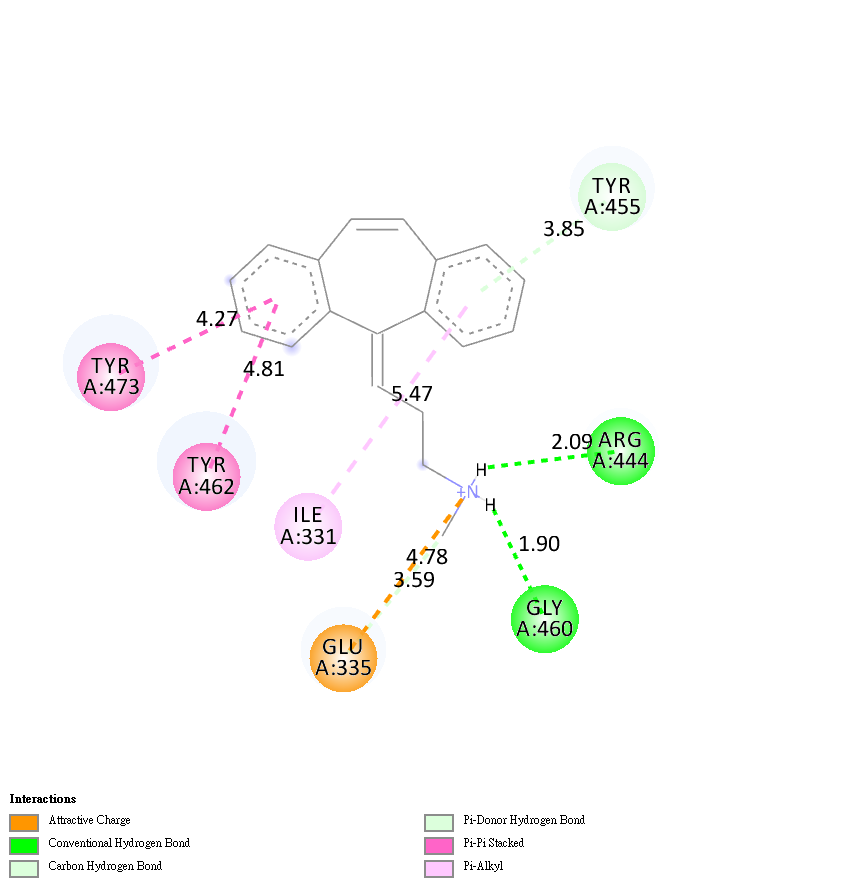

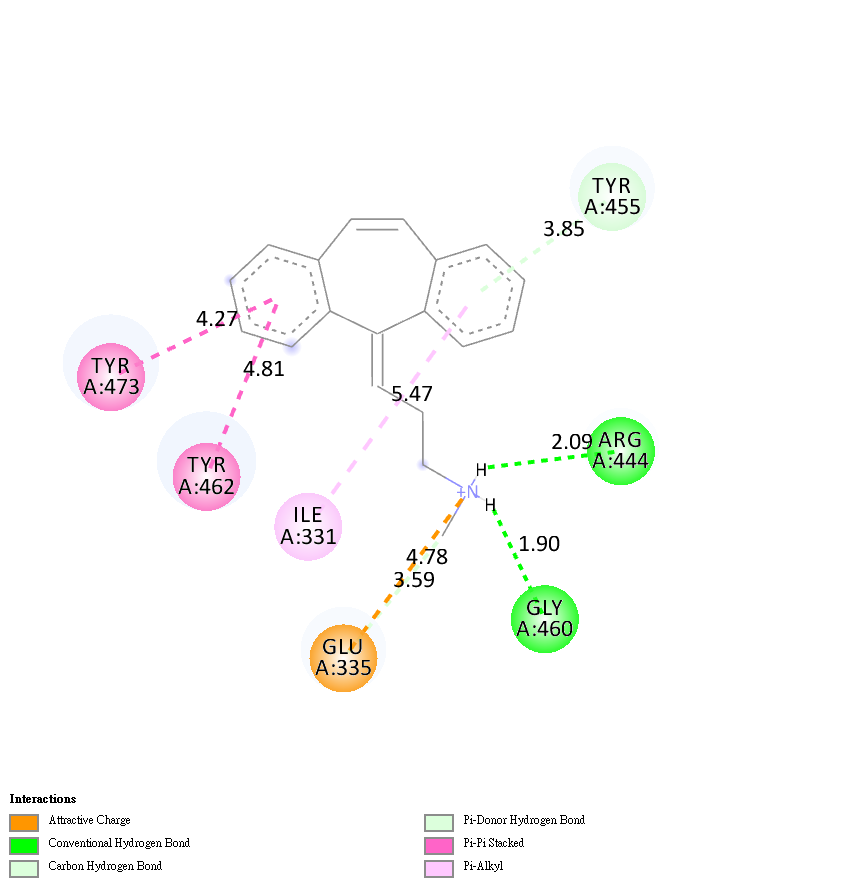


(C) (D)


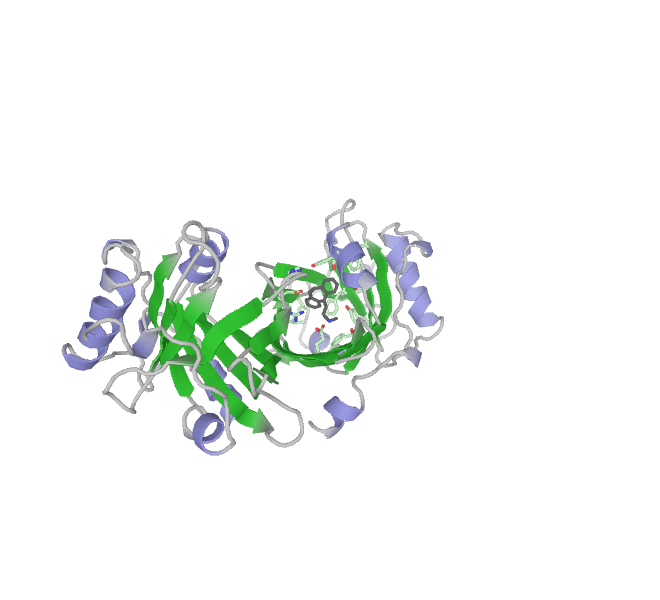

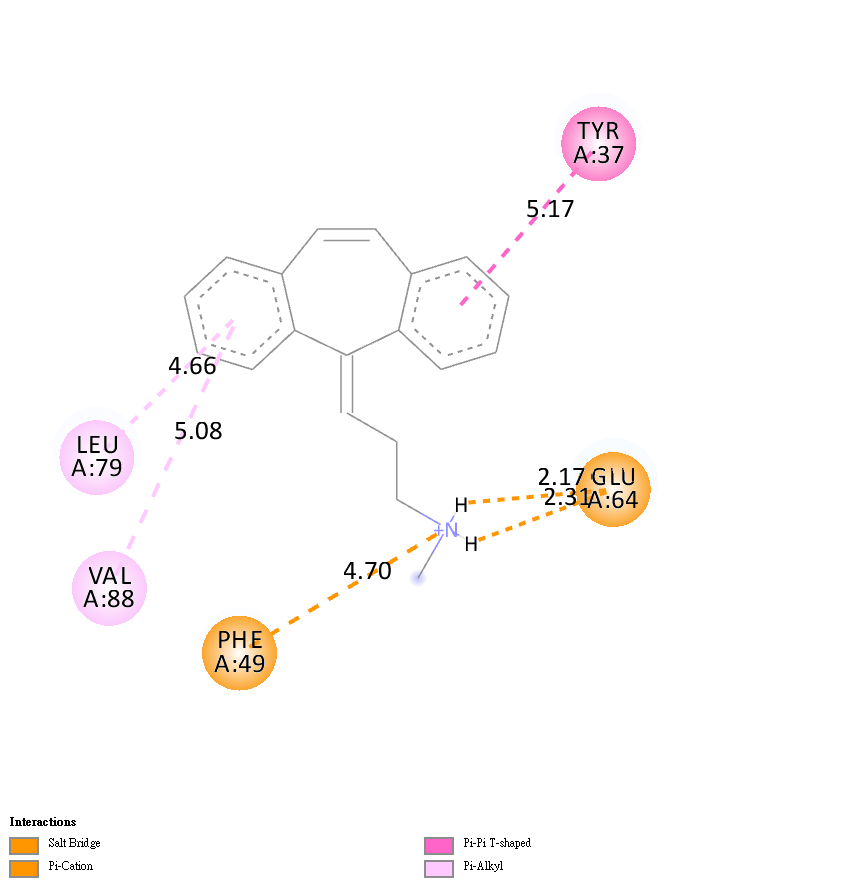

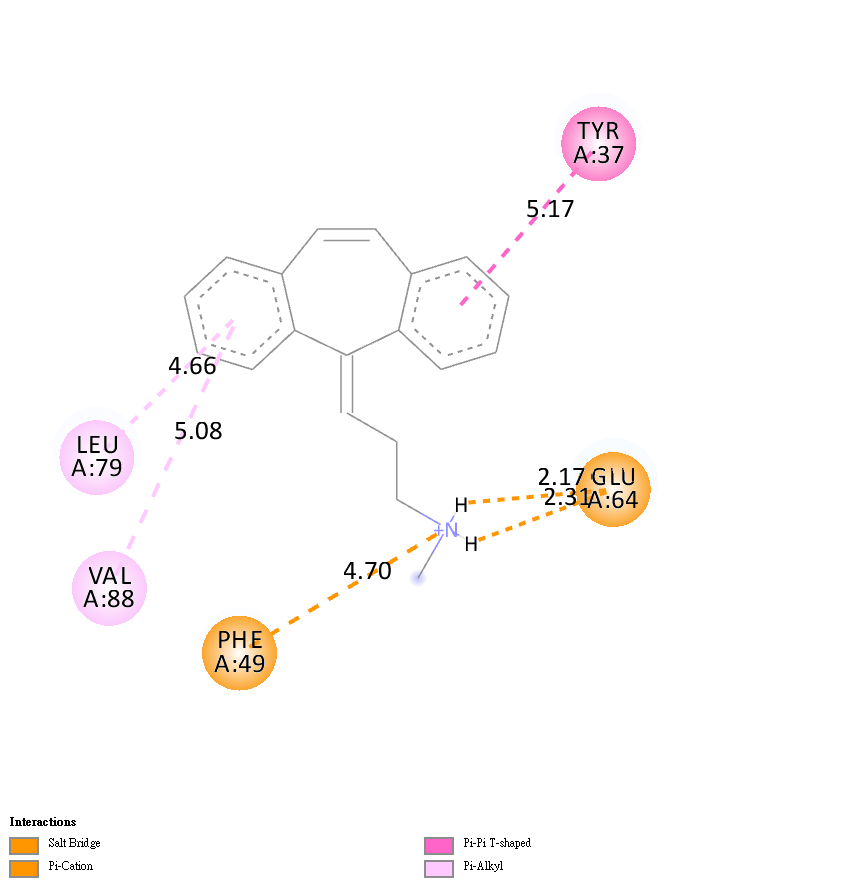

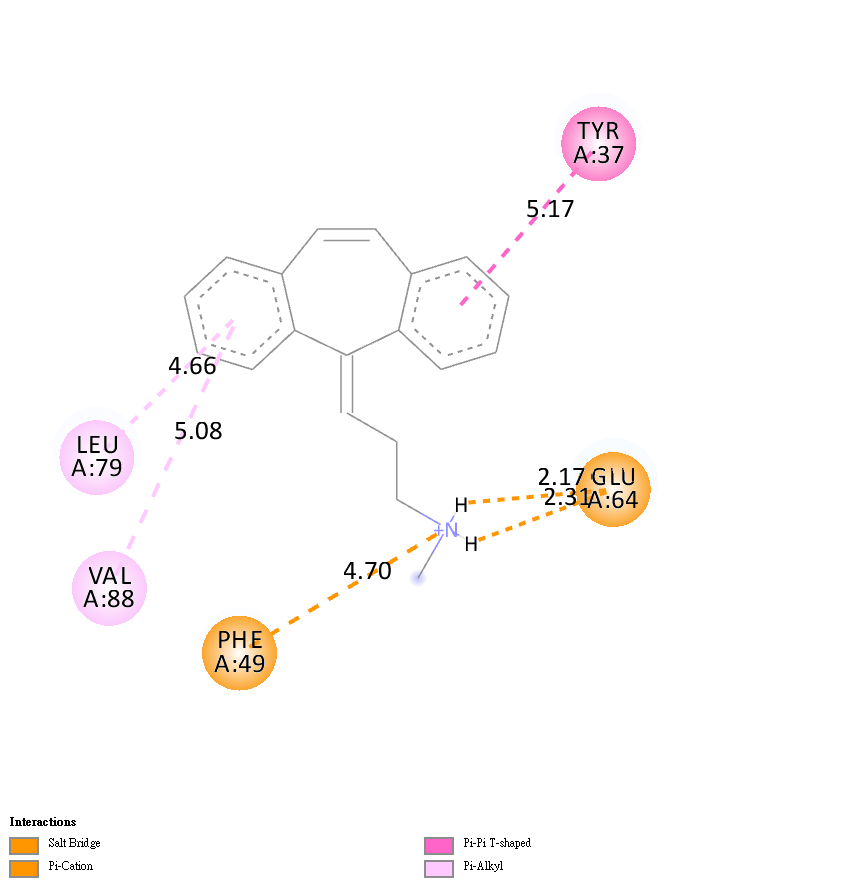

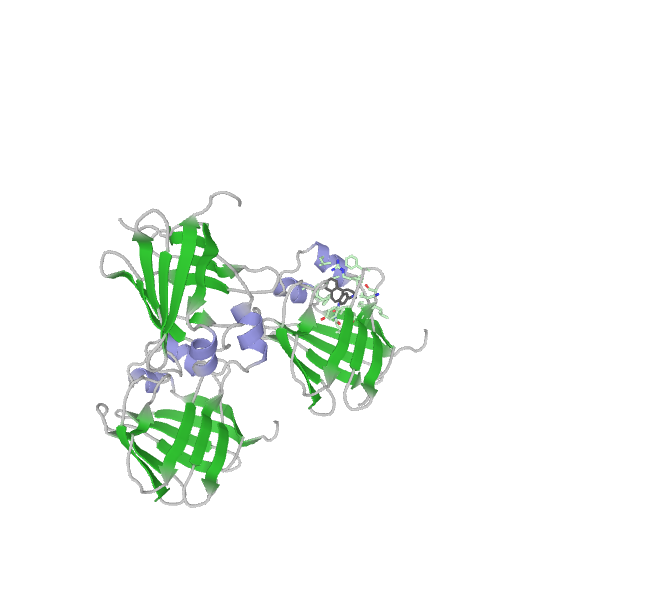

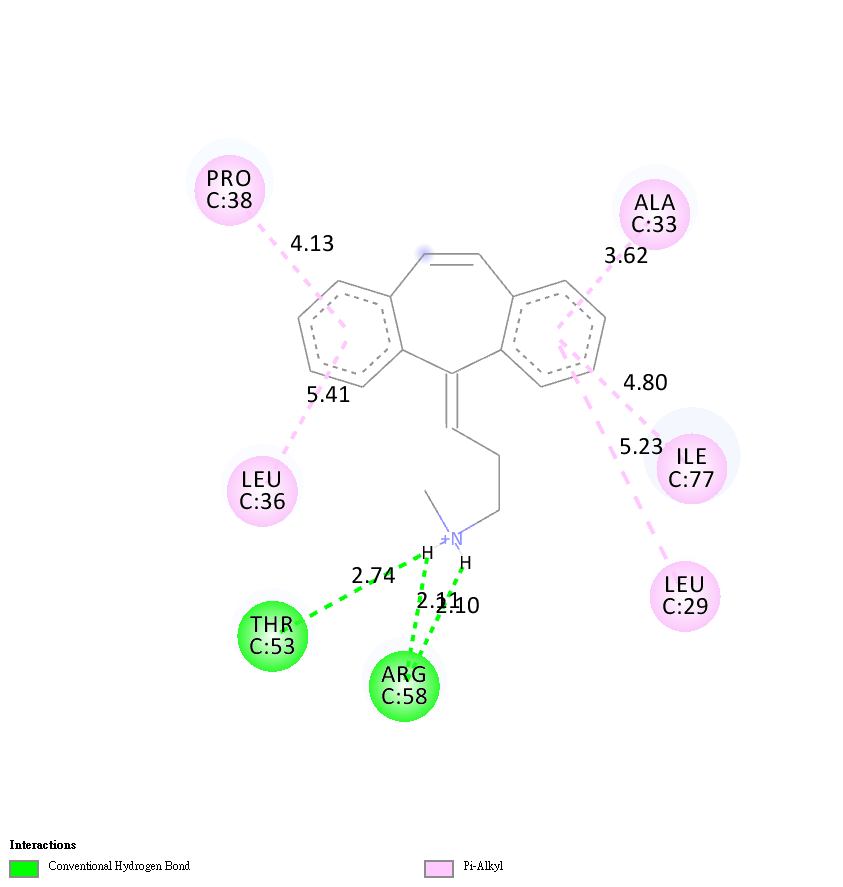

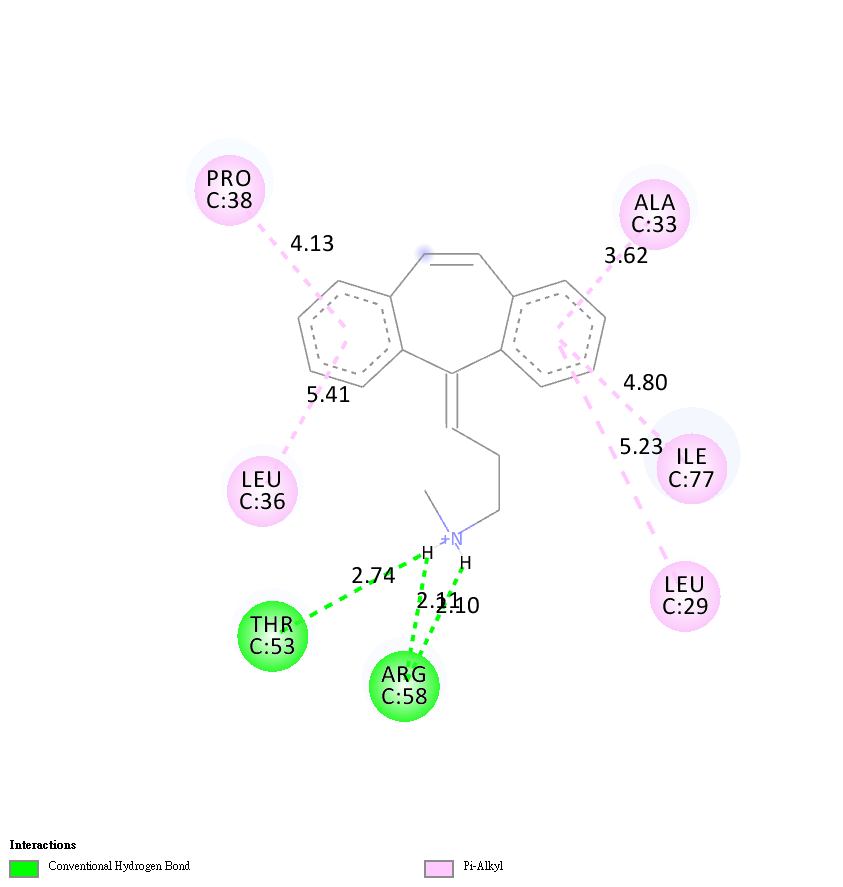

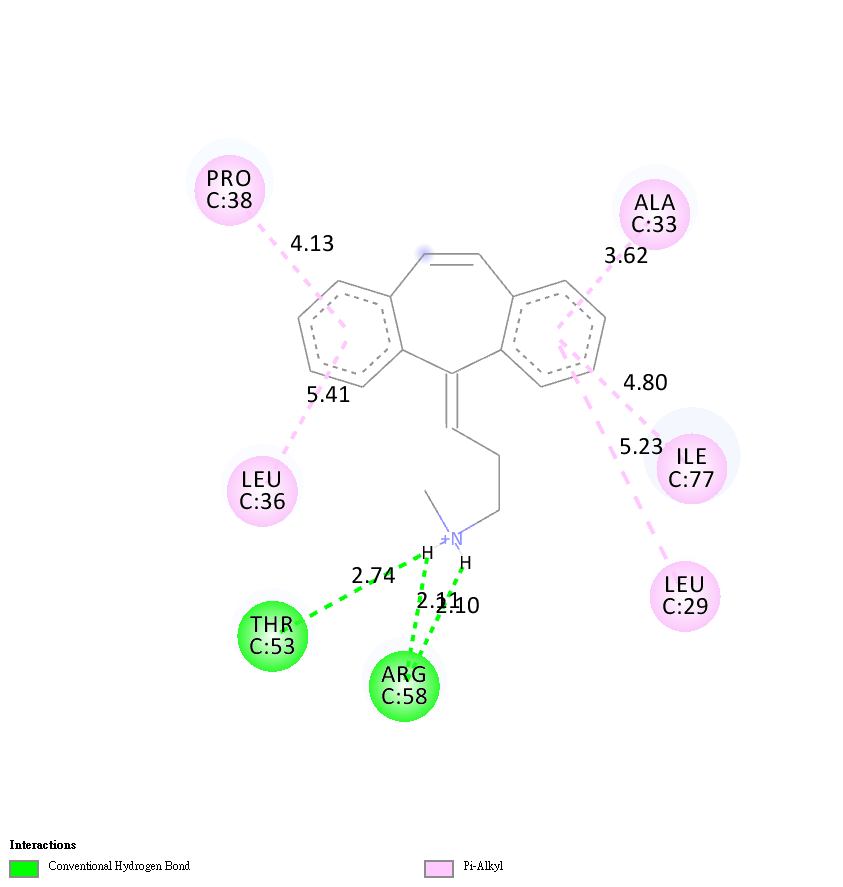


(E) (F)


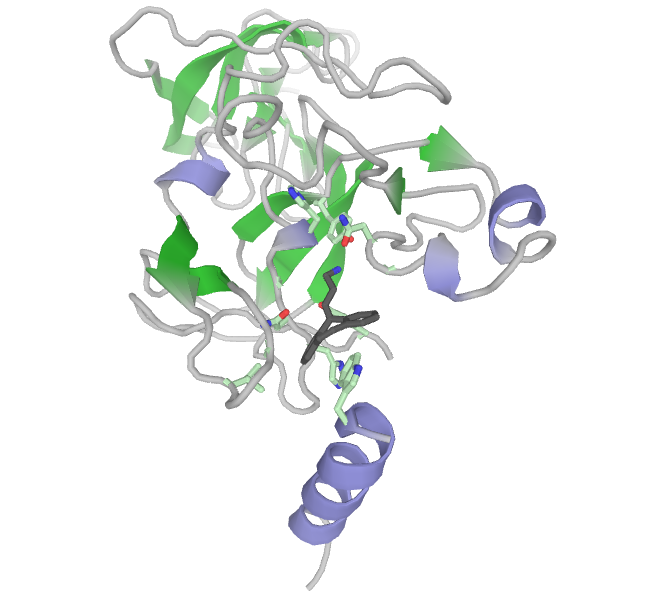

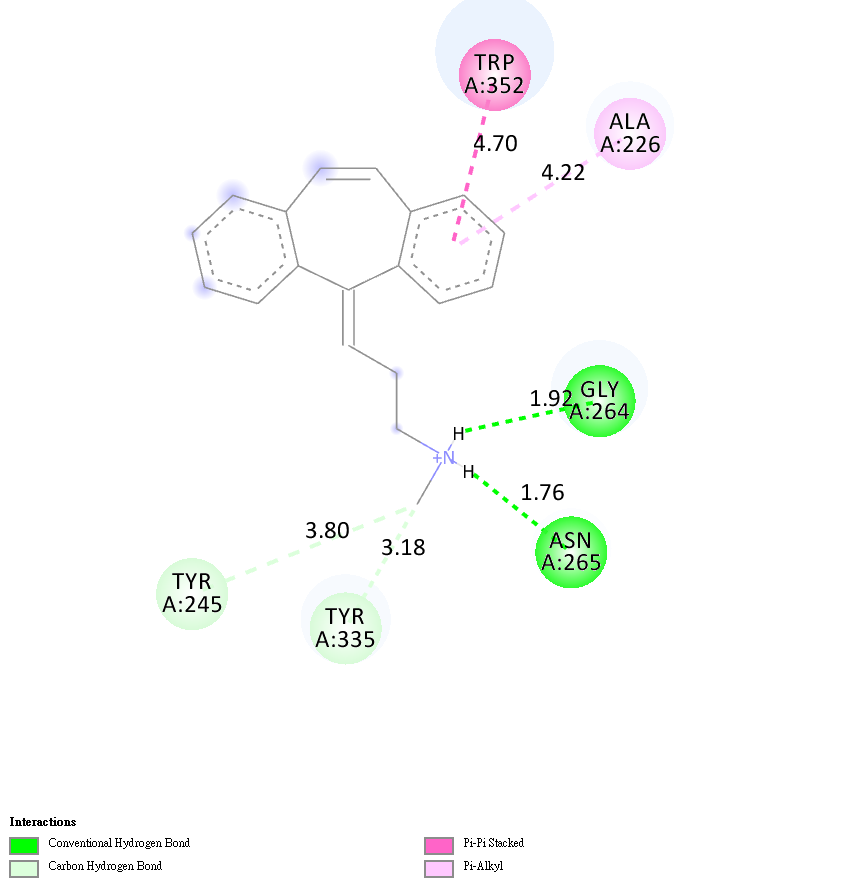

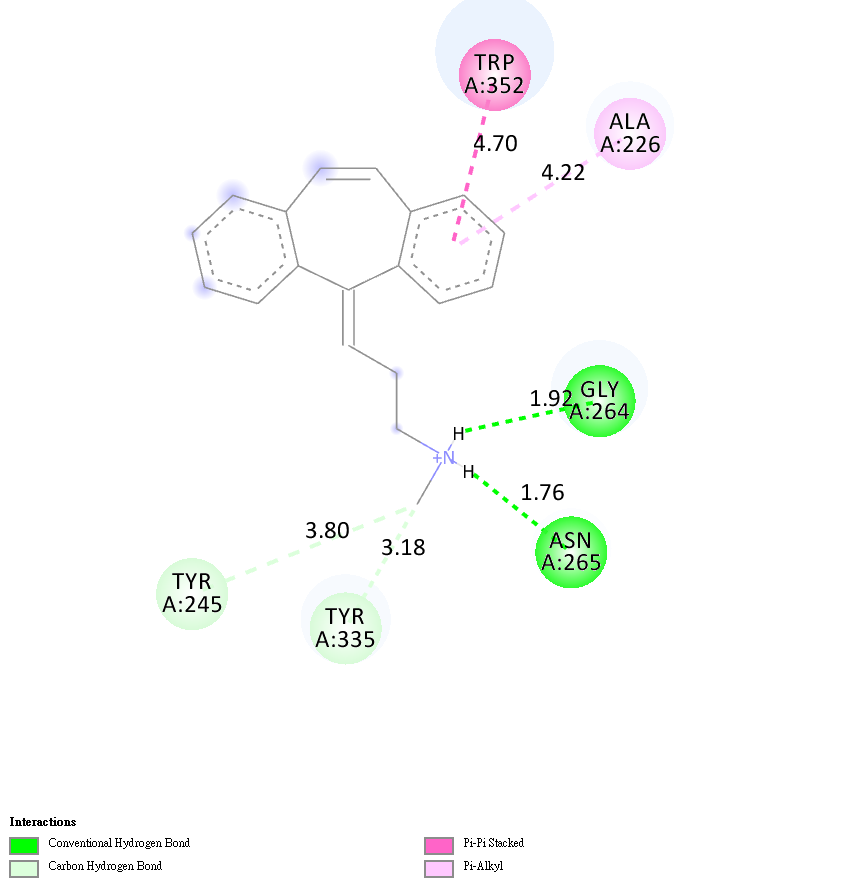

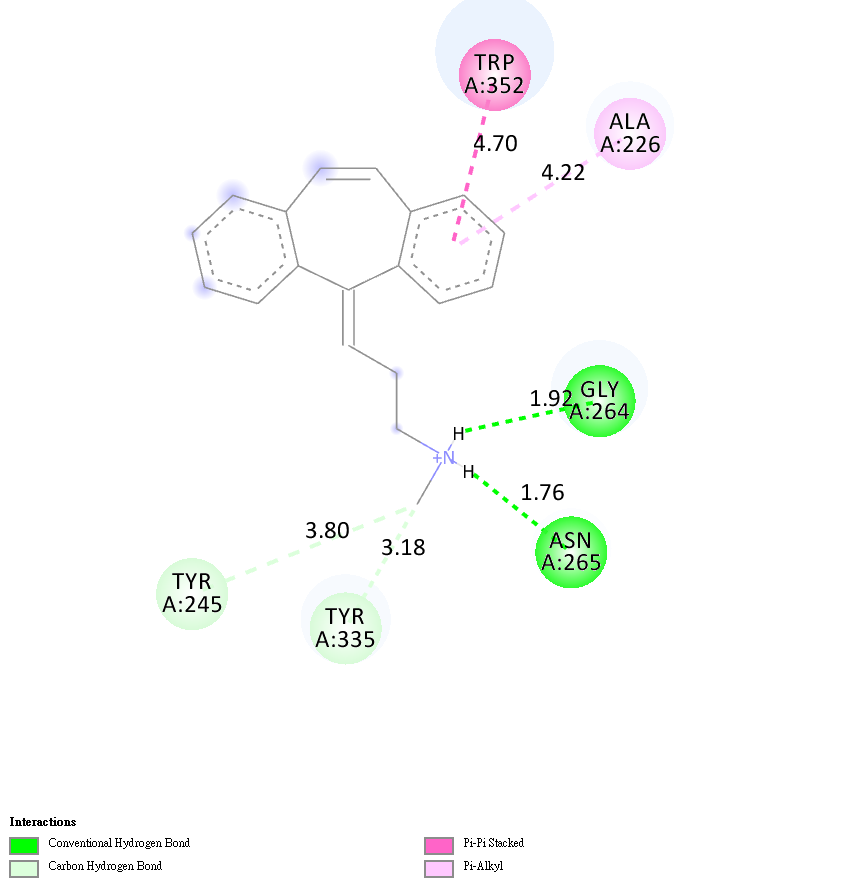

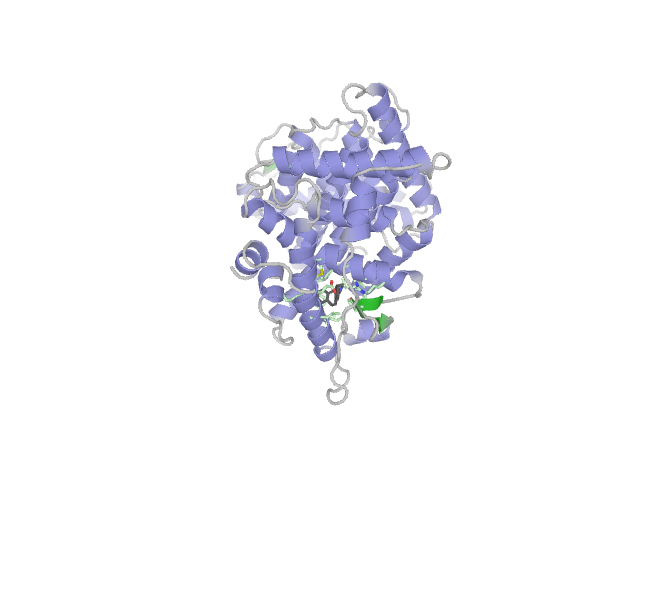

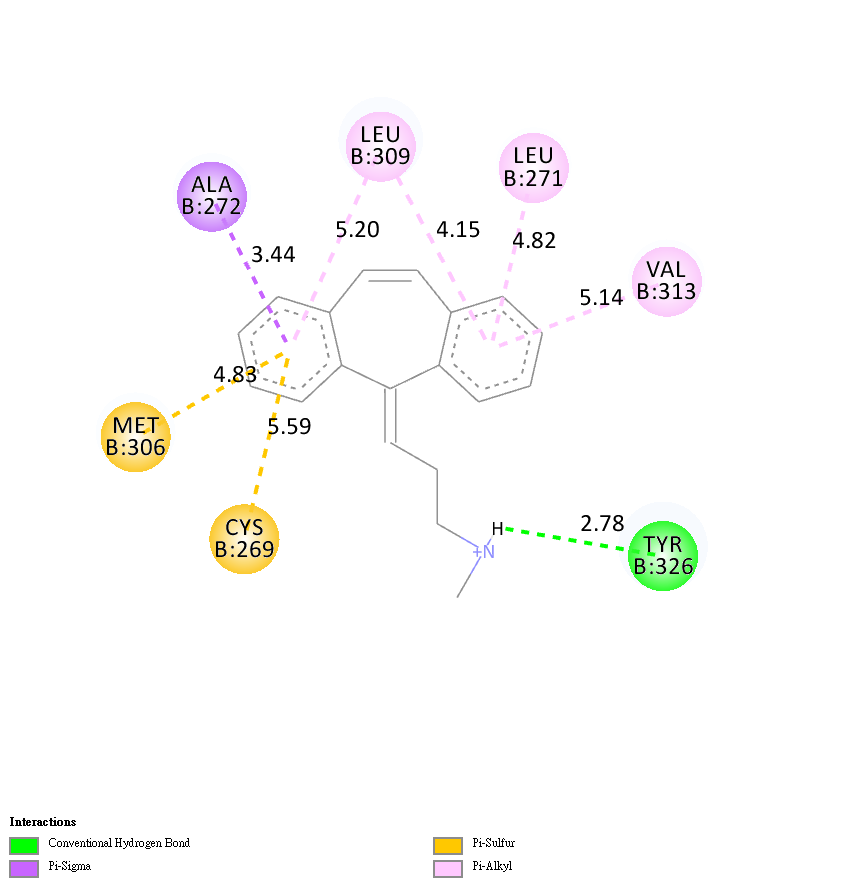

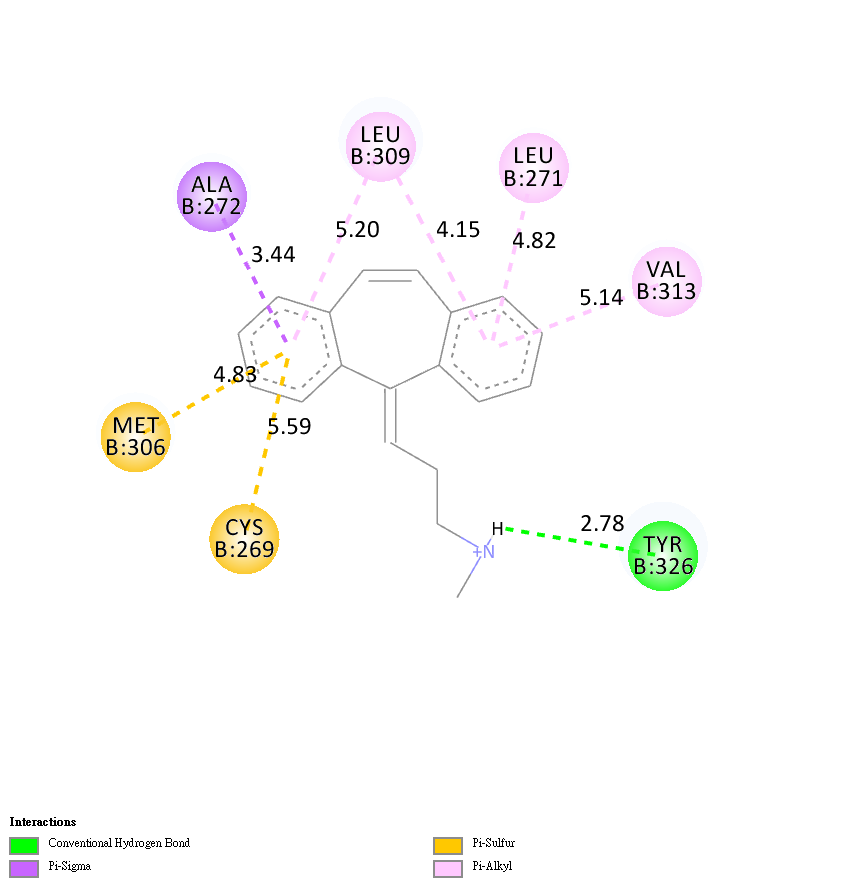

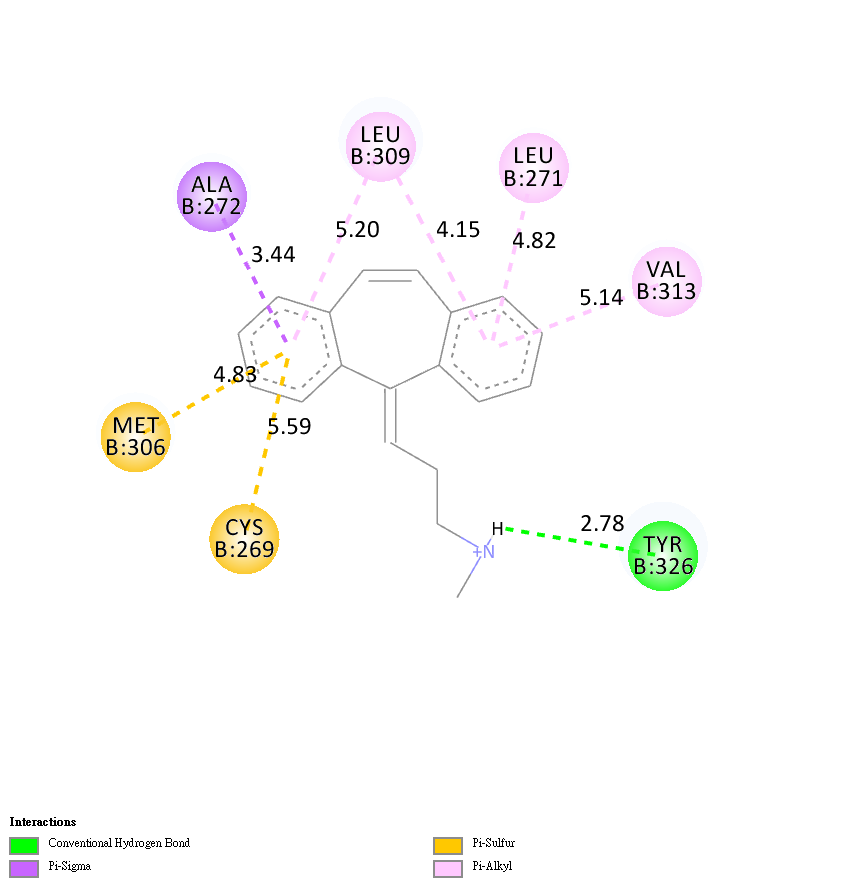


(G) (H)


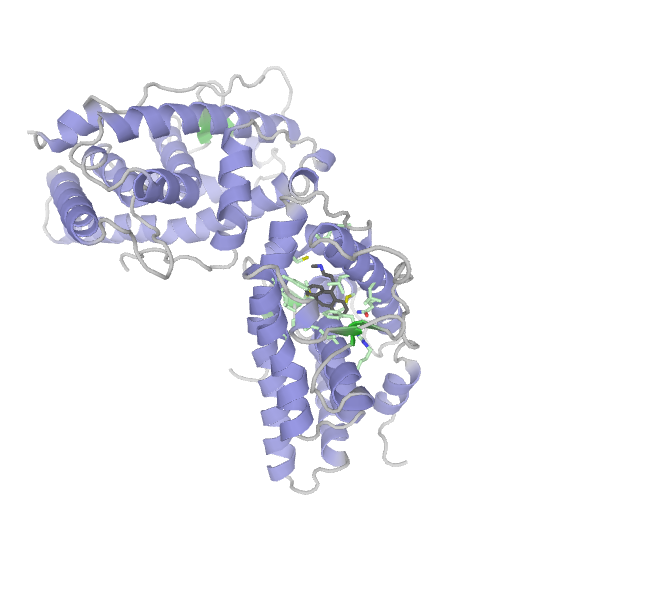

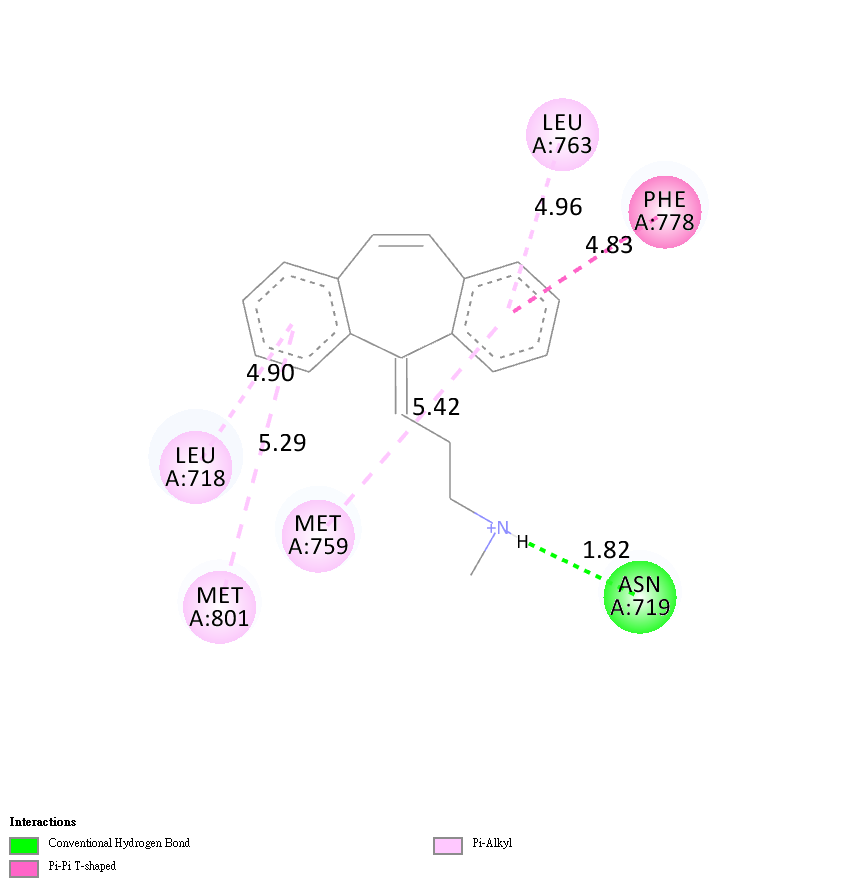

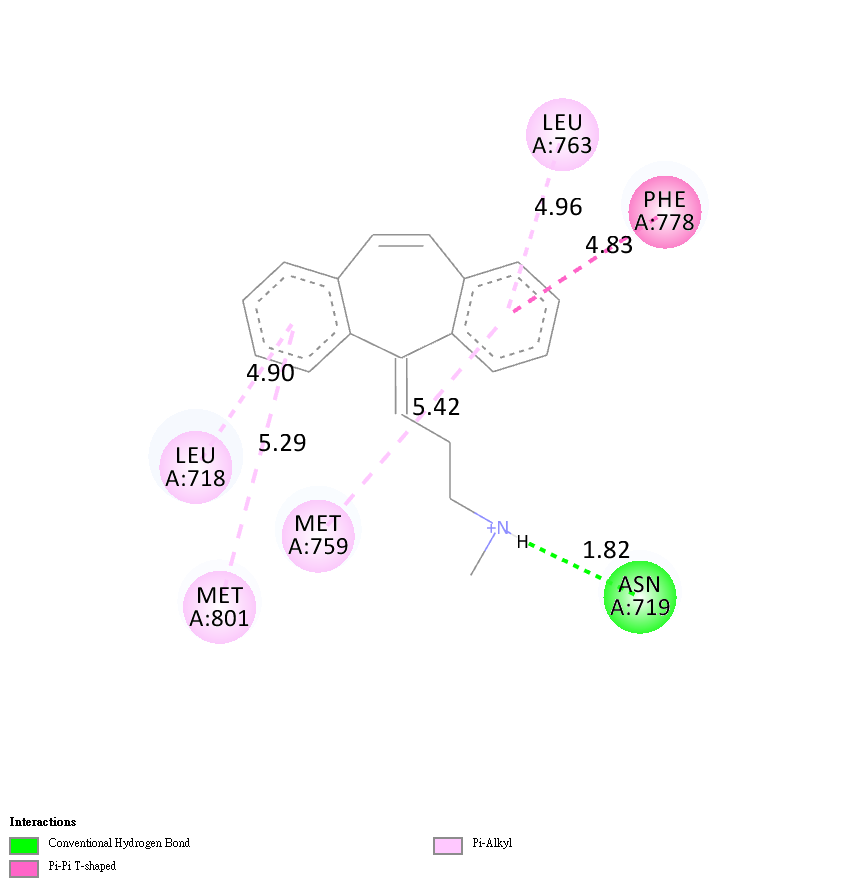

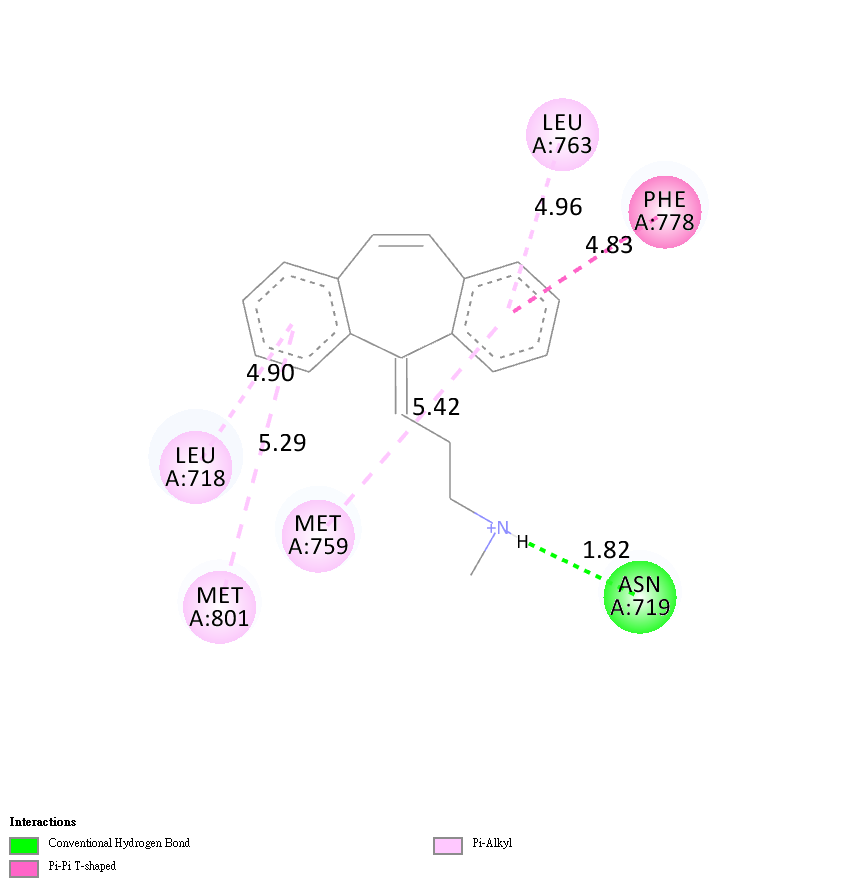

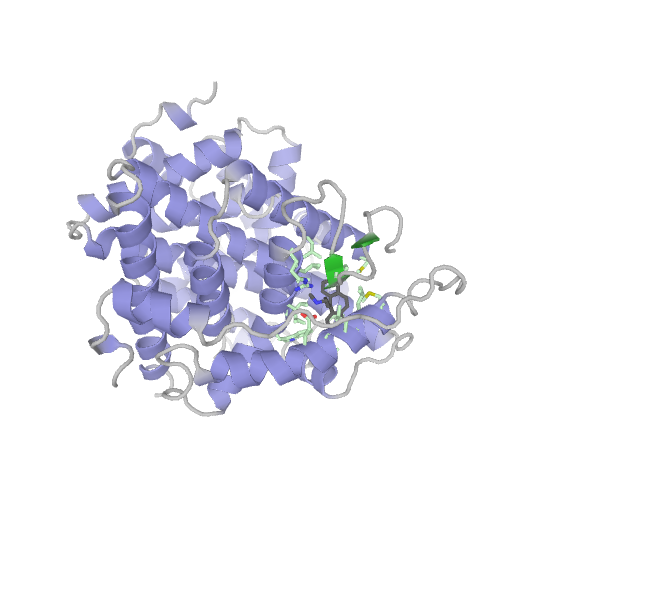

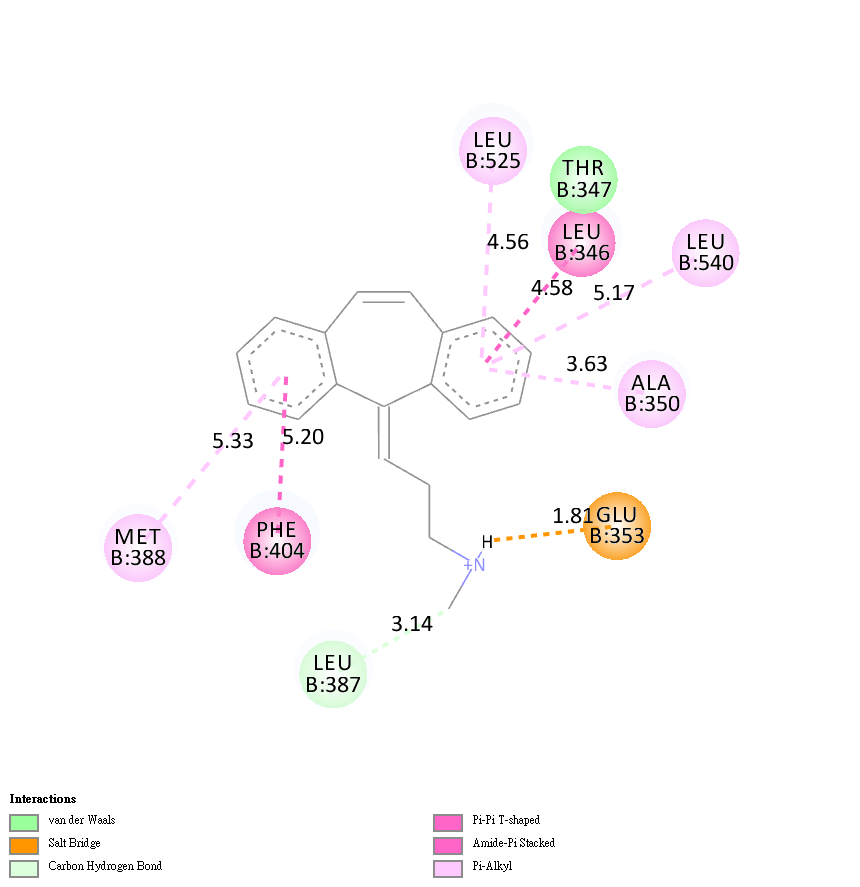

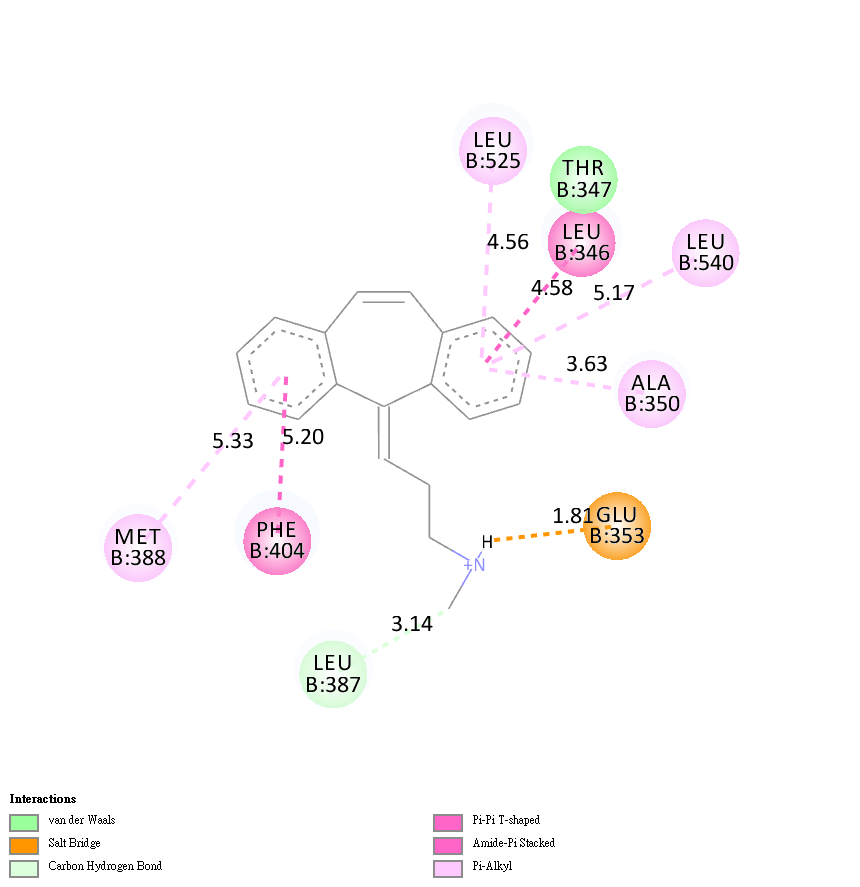

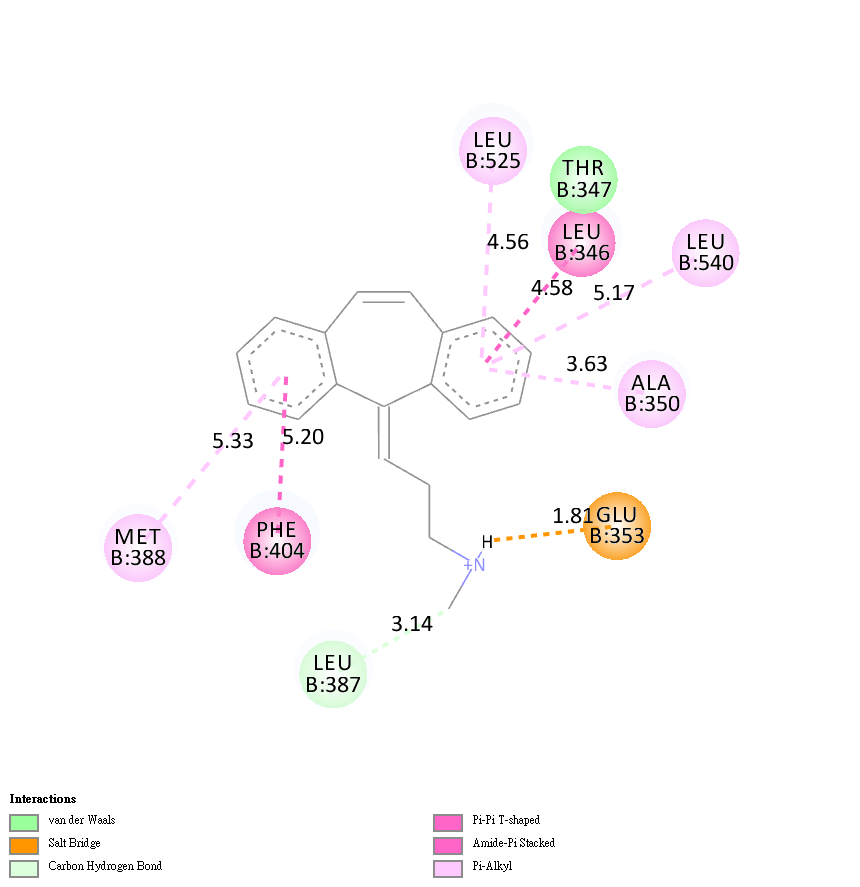


(I) (J)


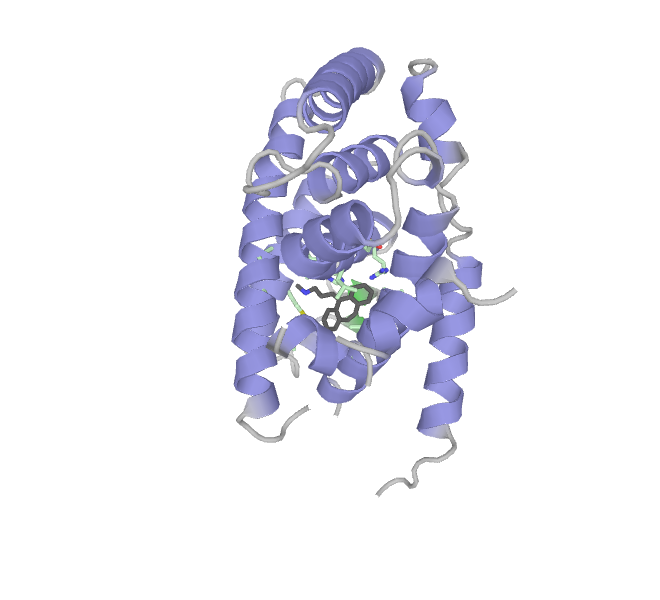

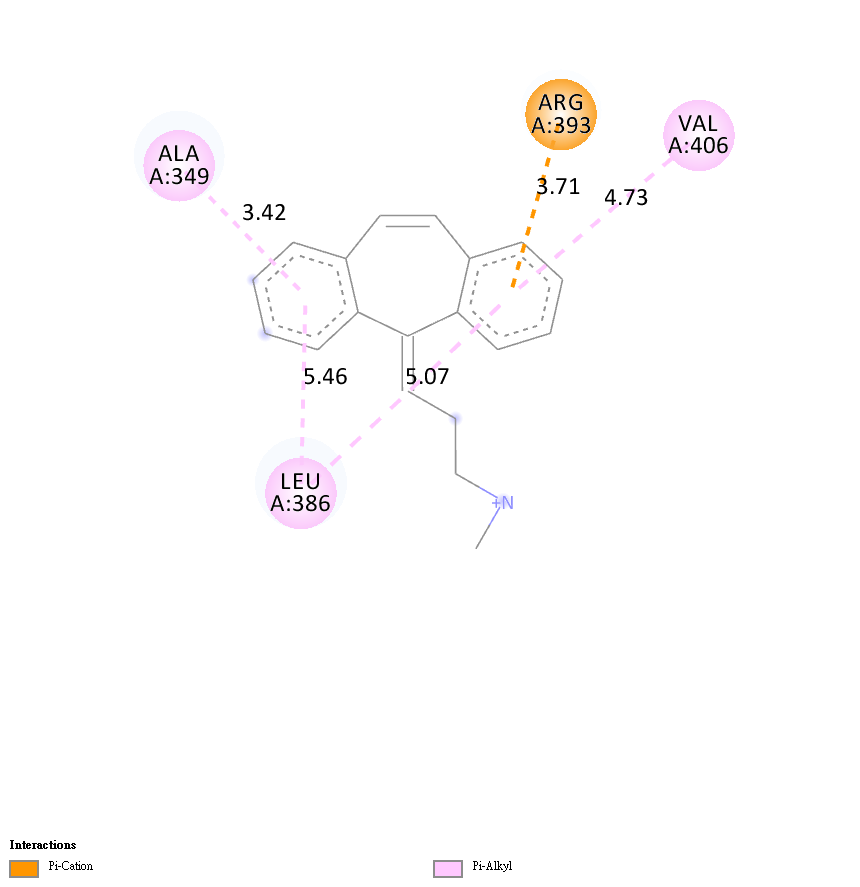

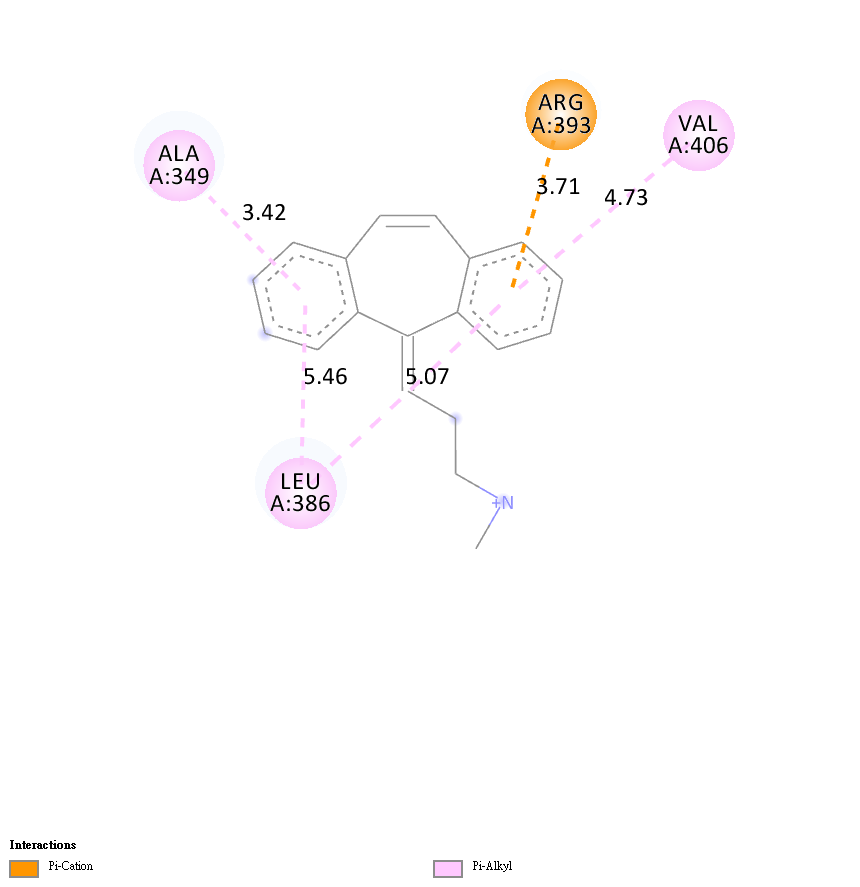

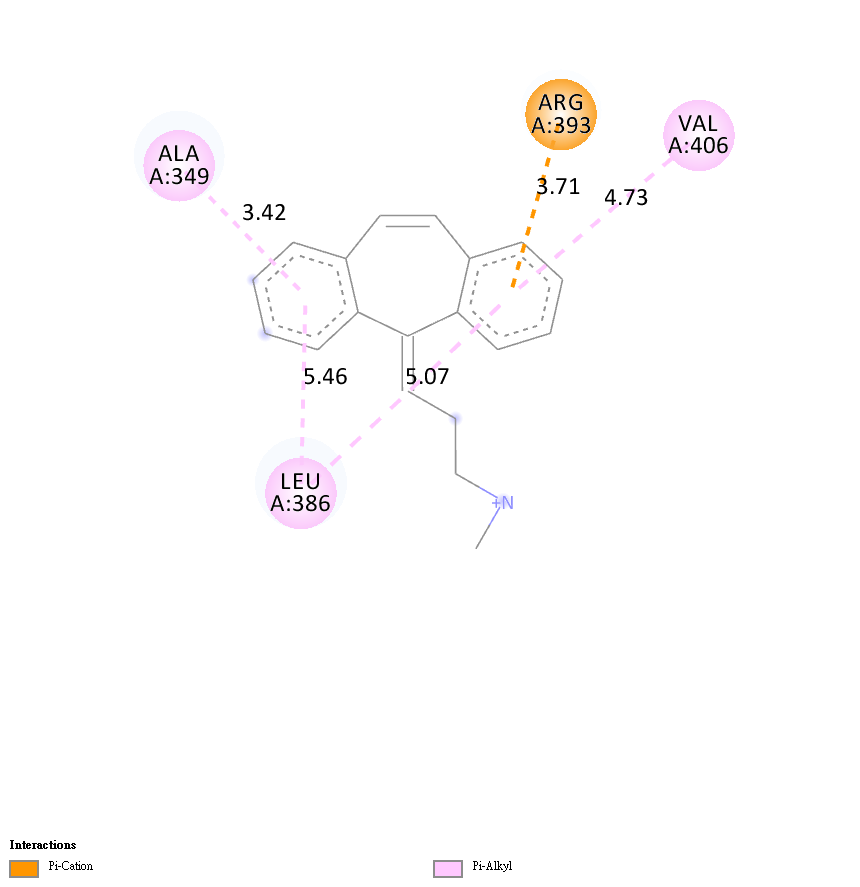

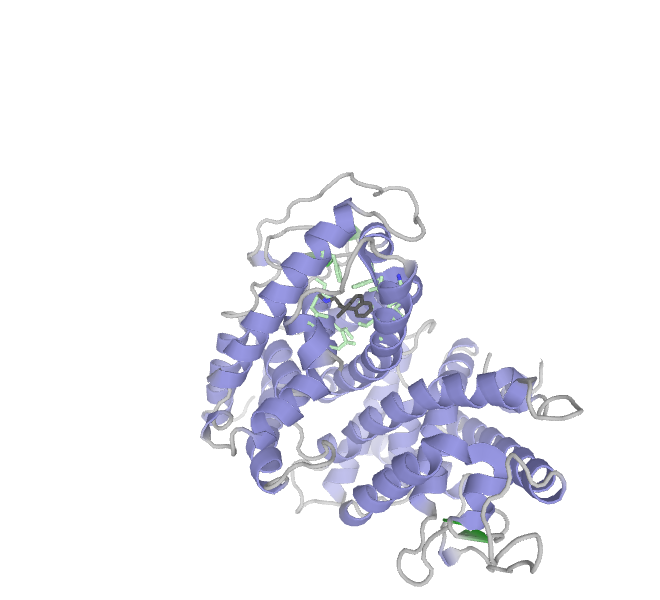

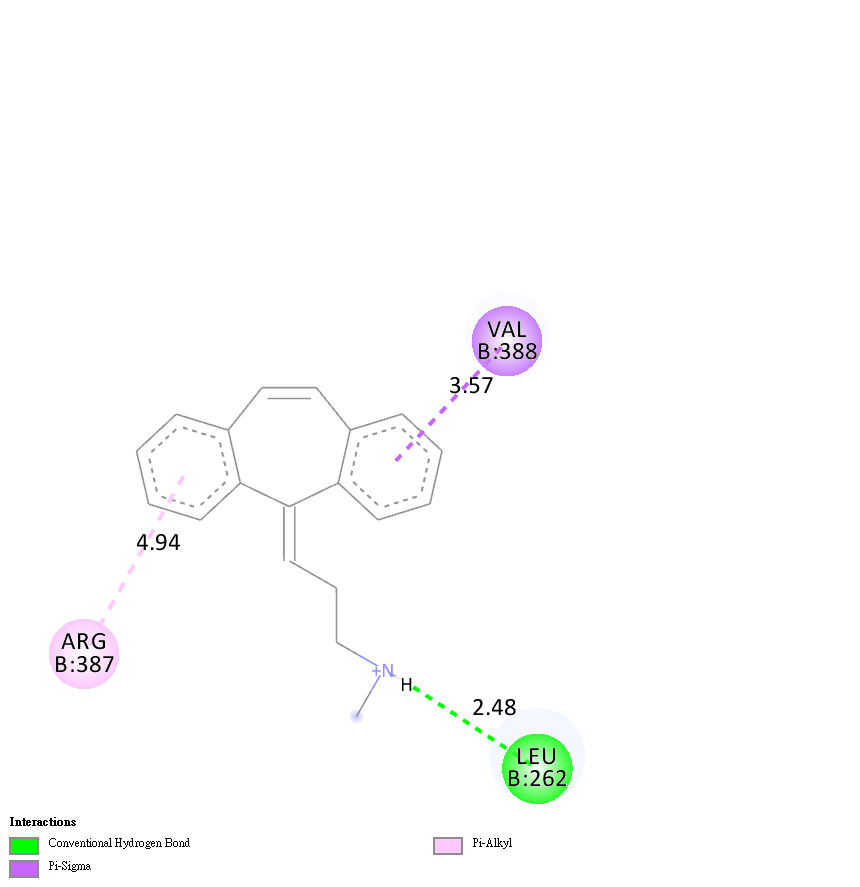

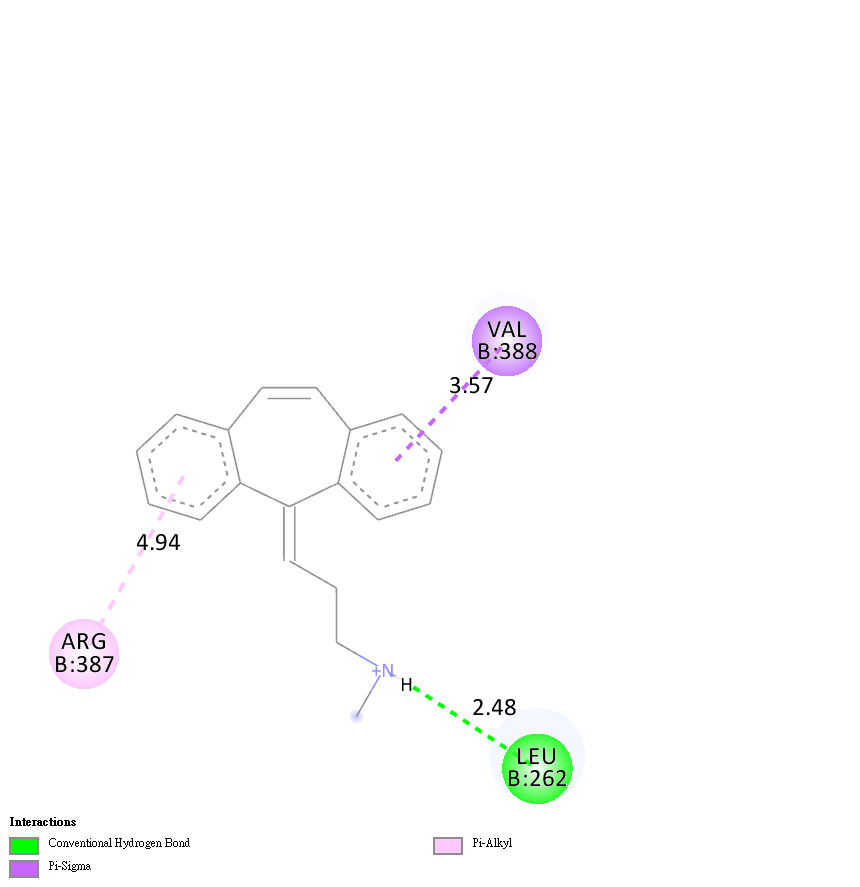

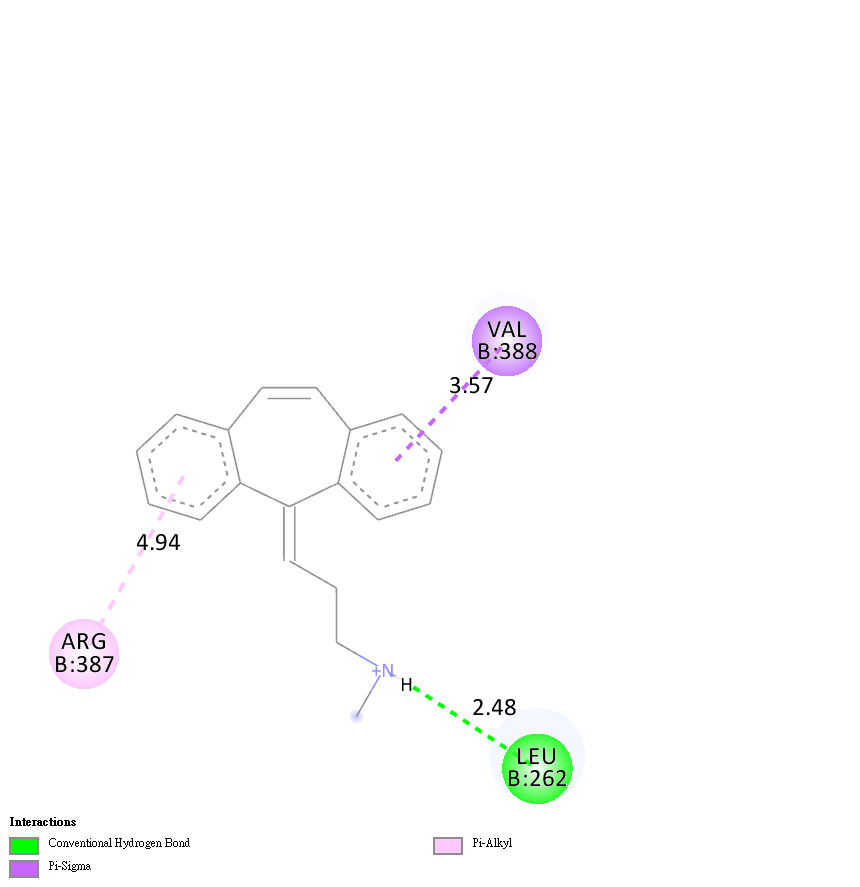


(K) (L)


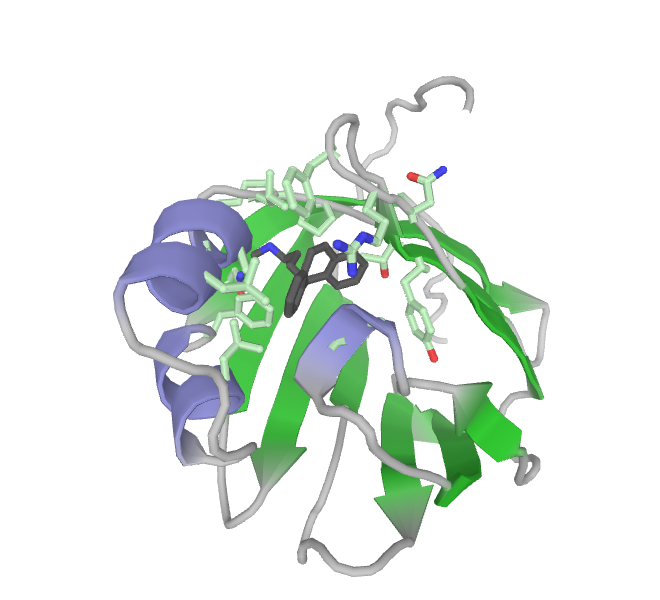

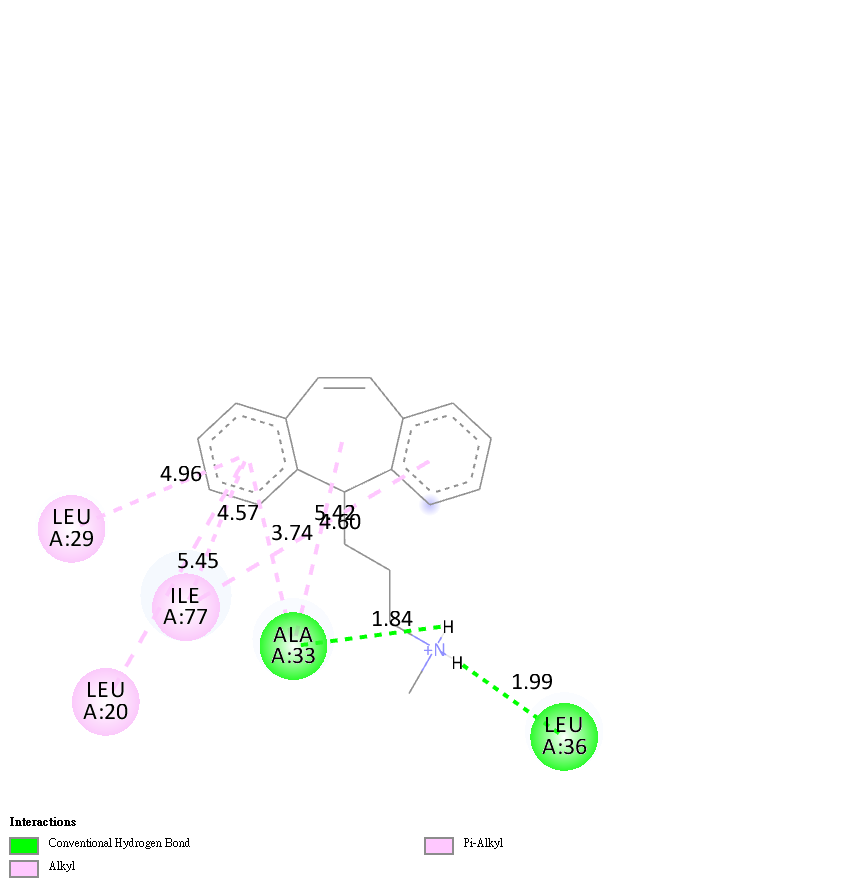

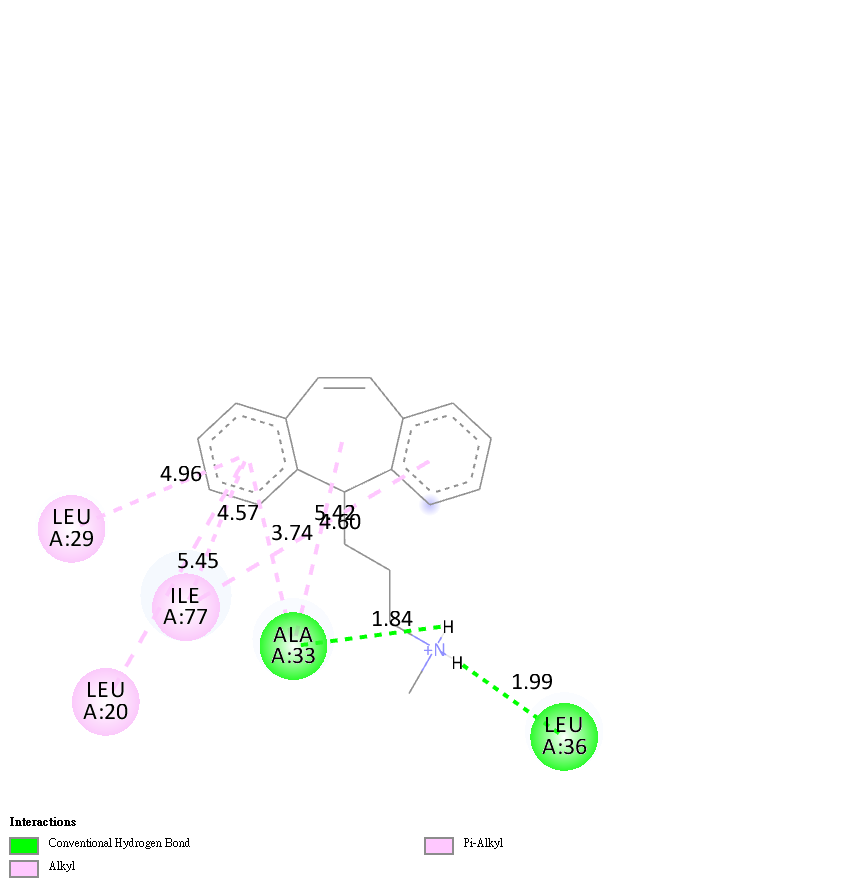

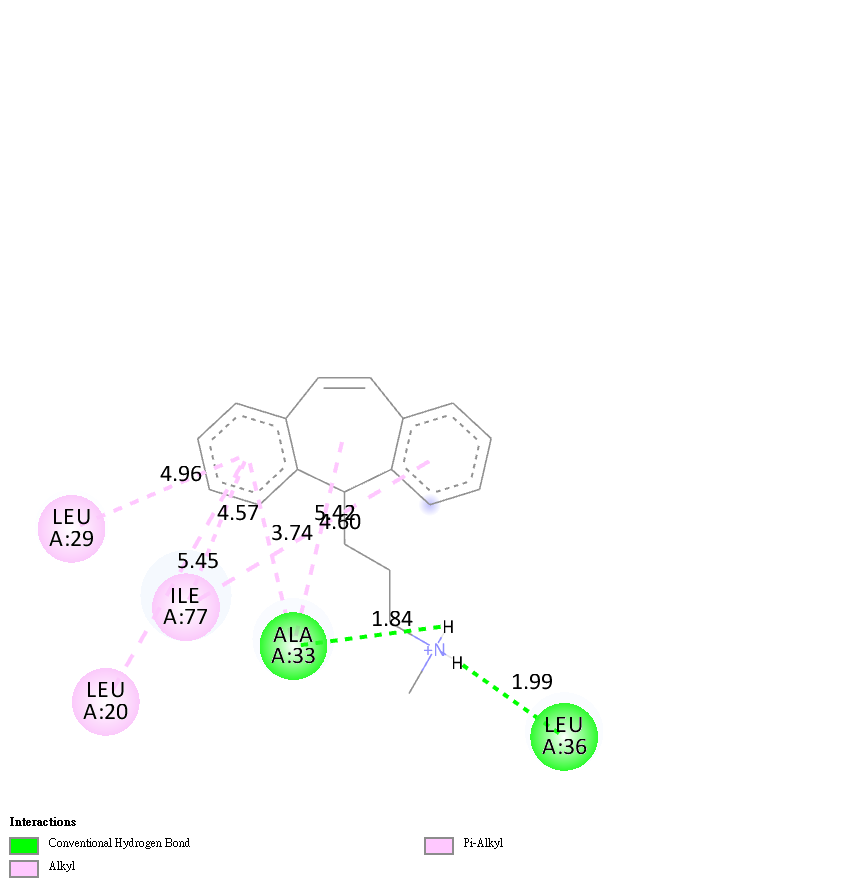

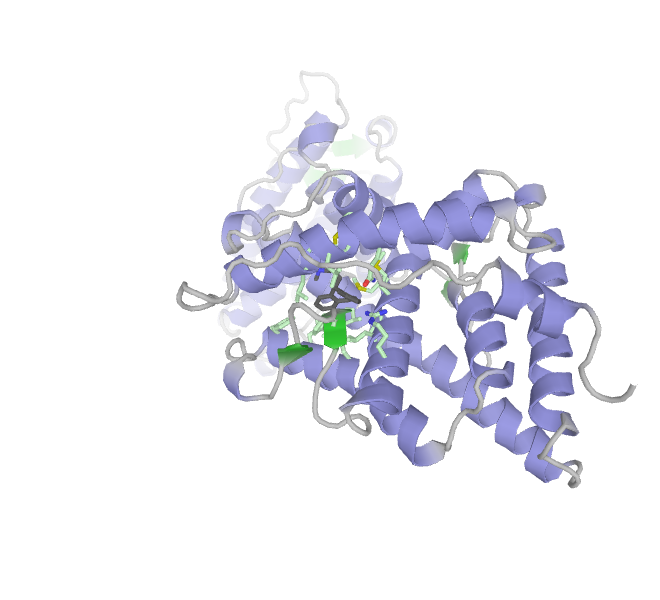

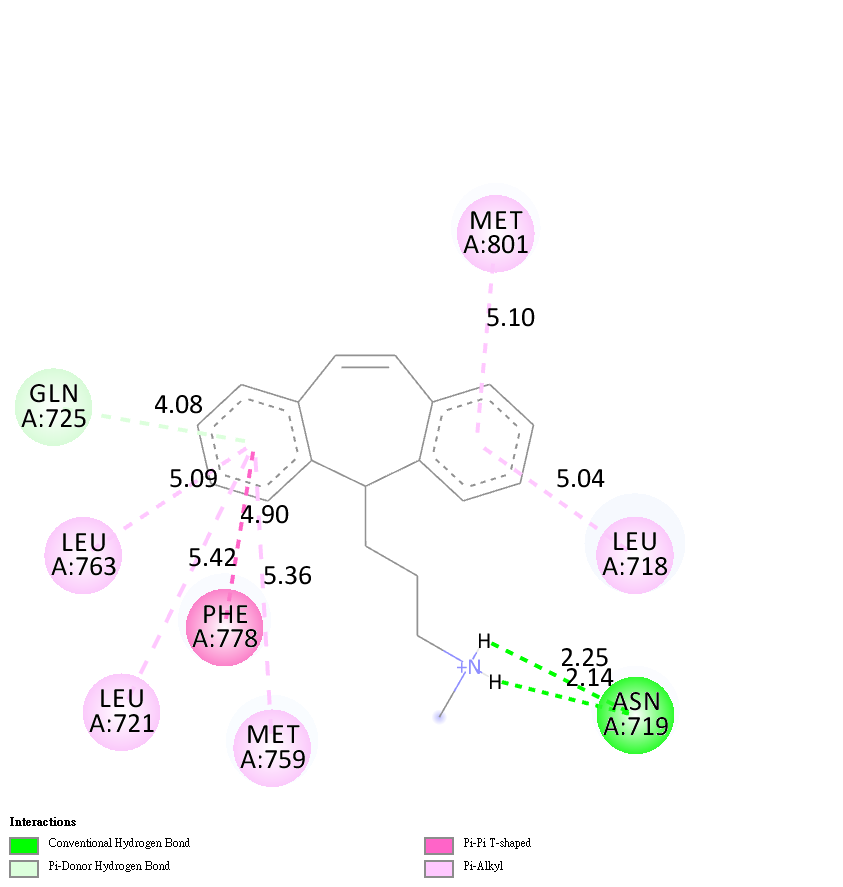

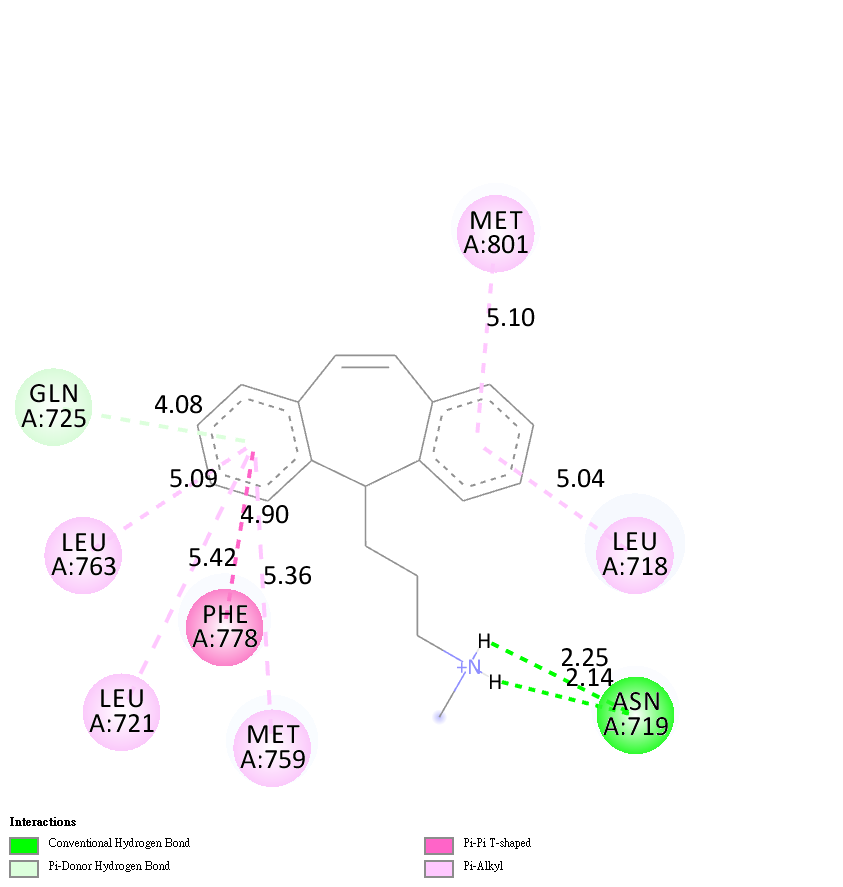

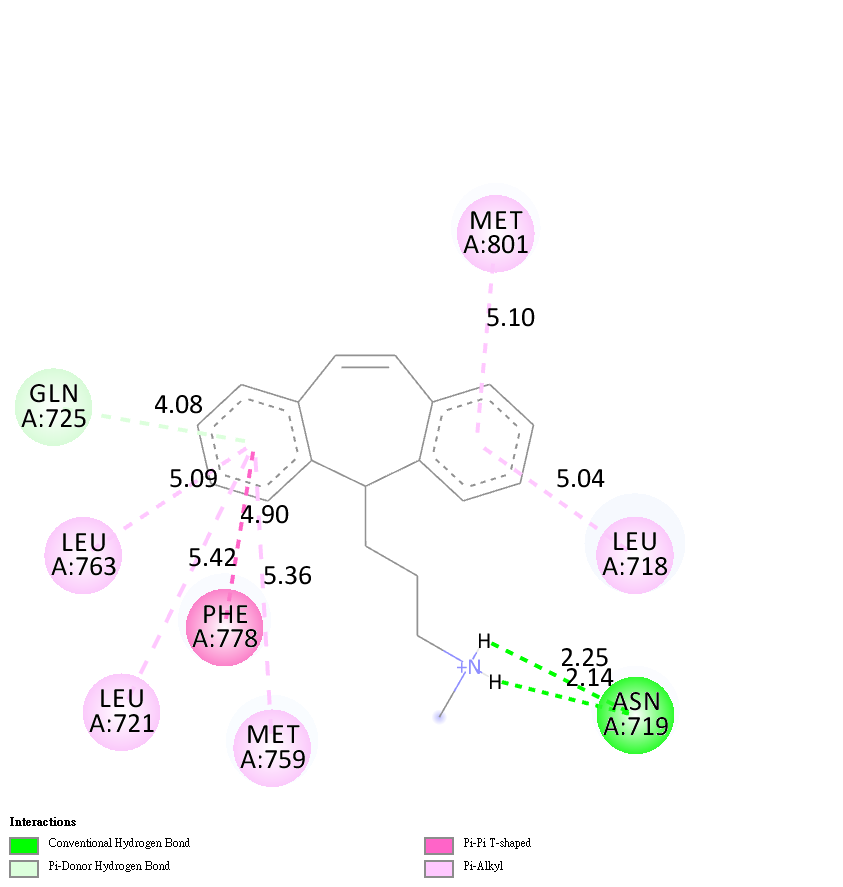


(M) (N)


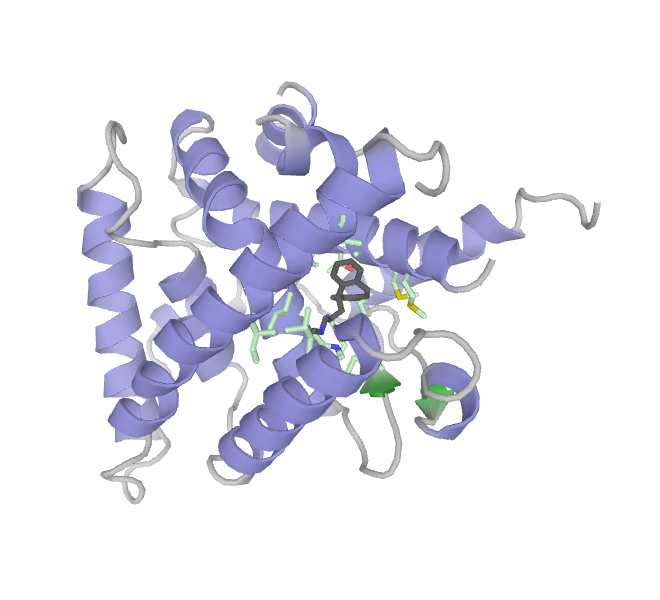

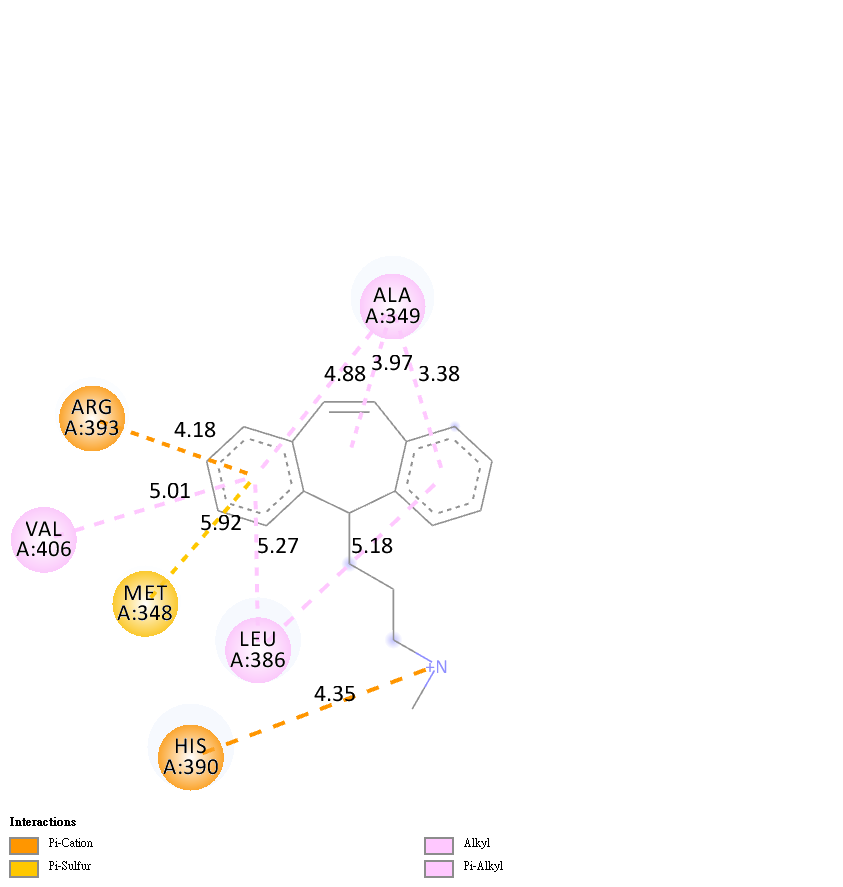

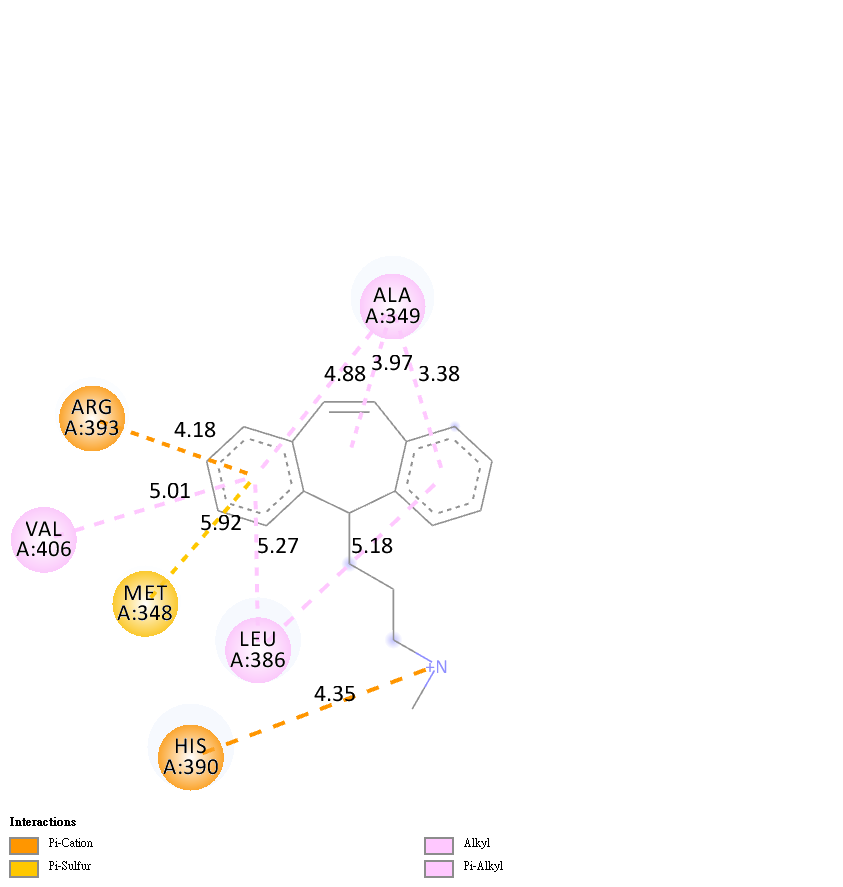

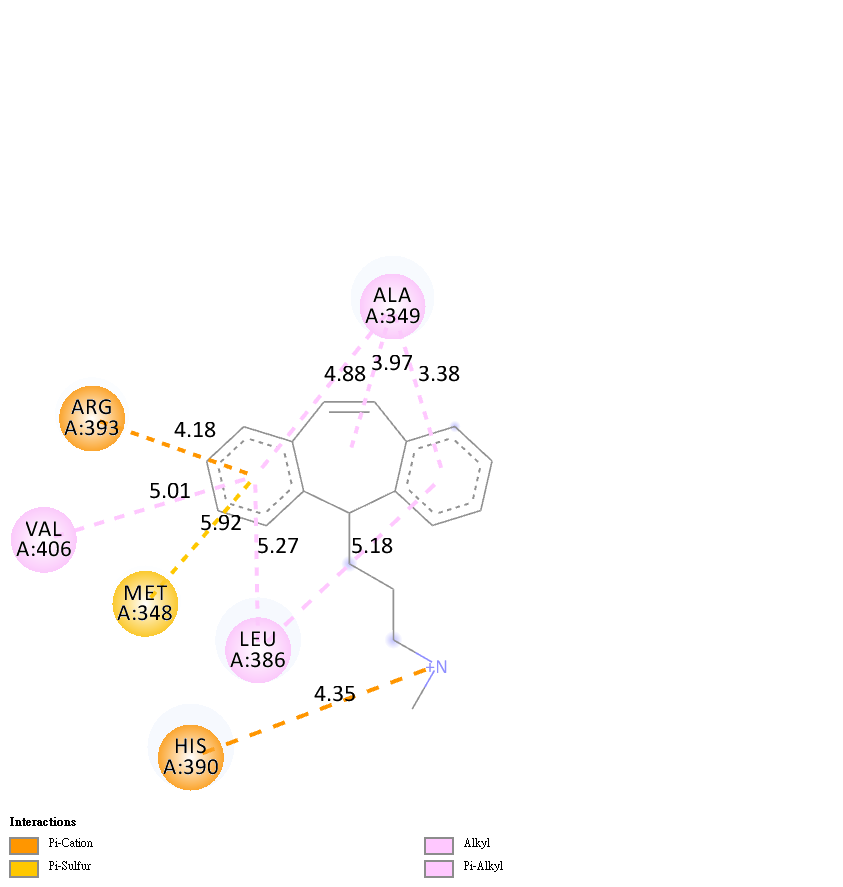

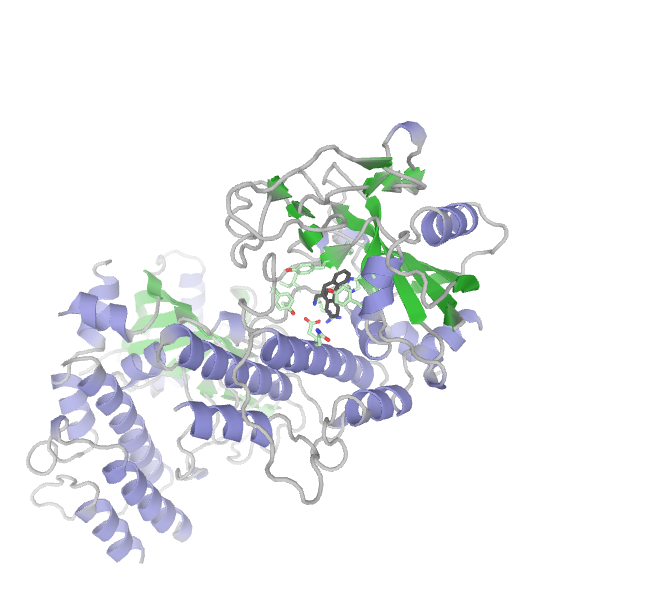

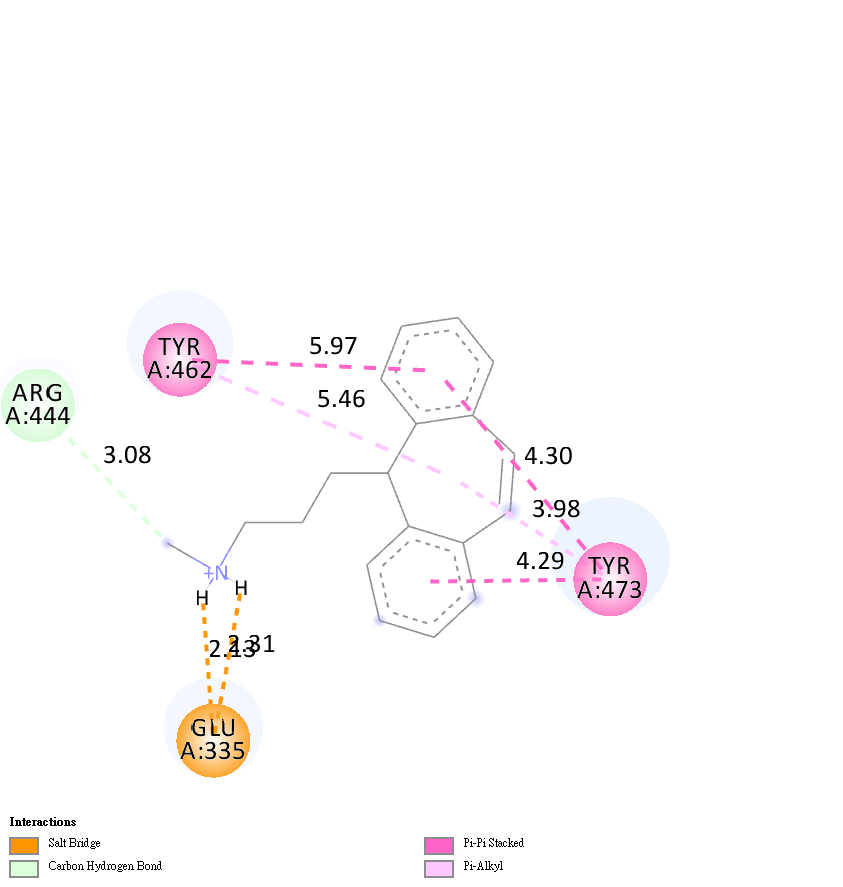

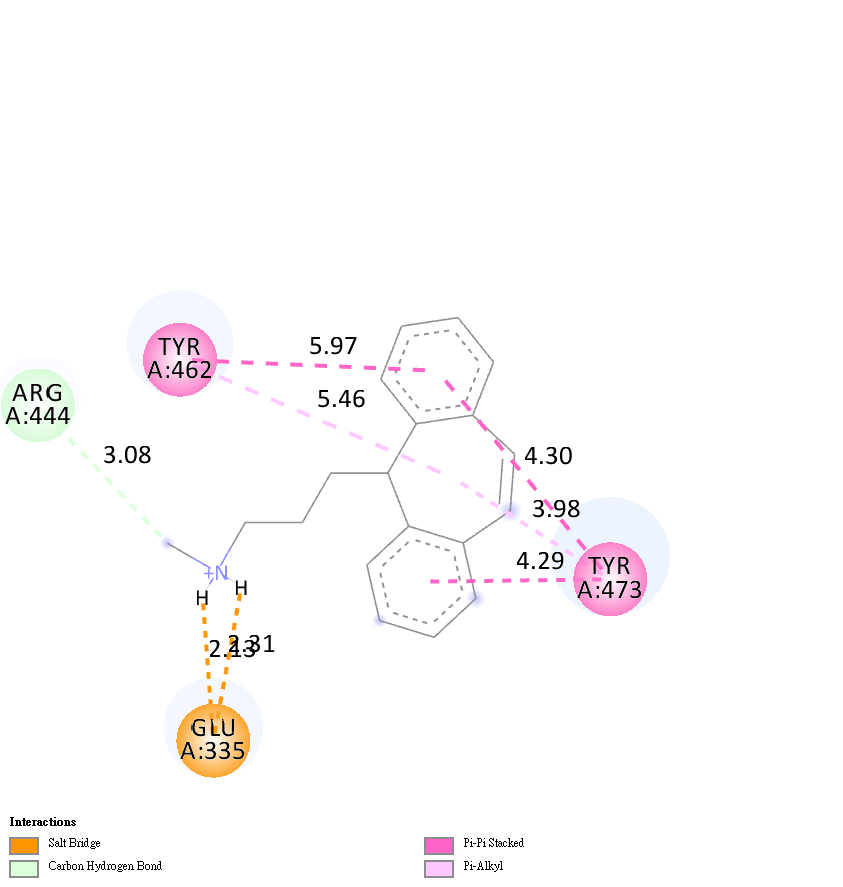

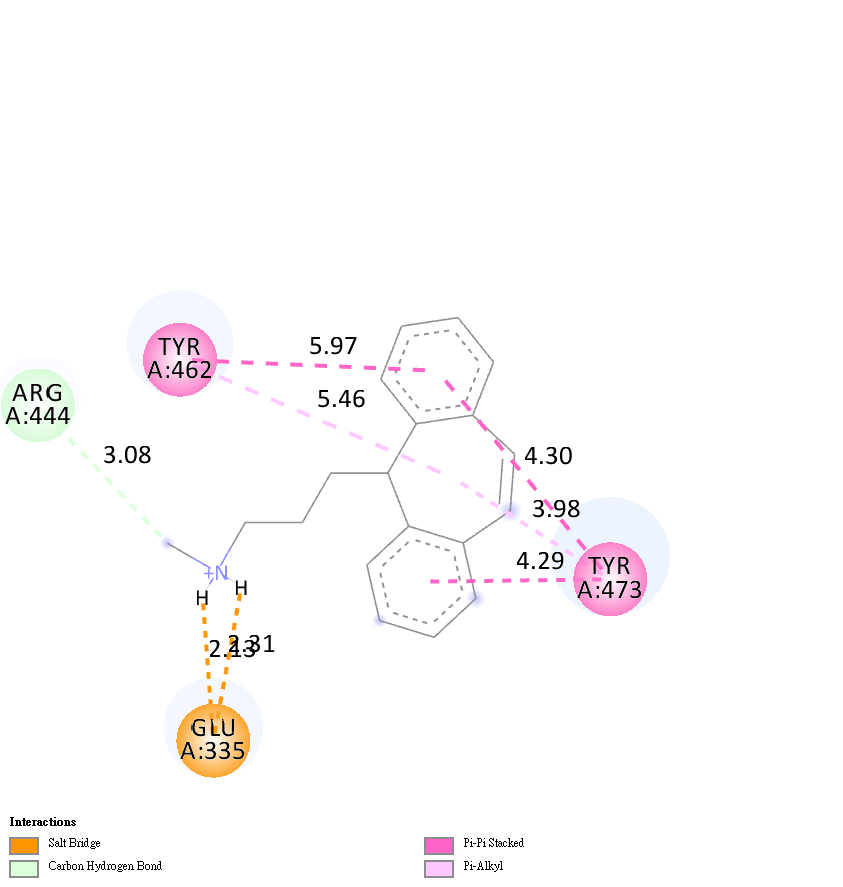


(O) (P)


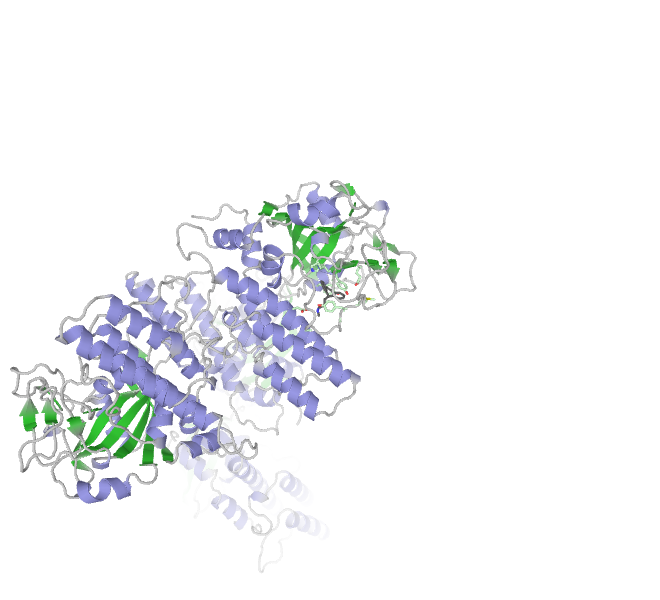

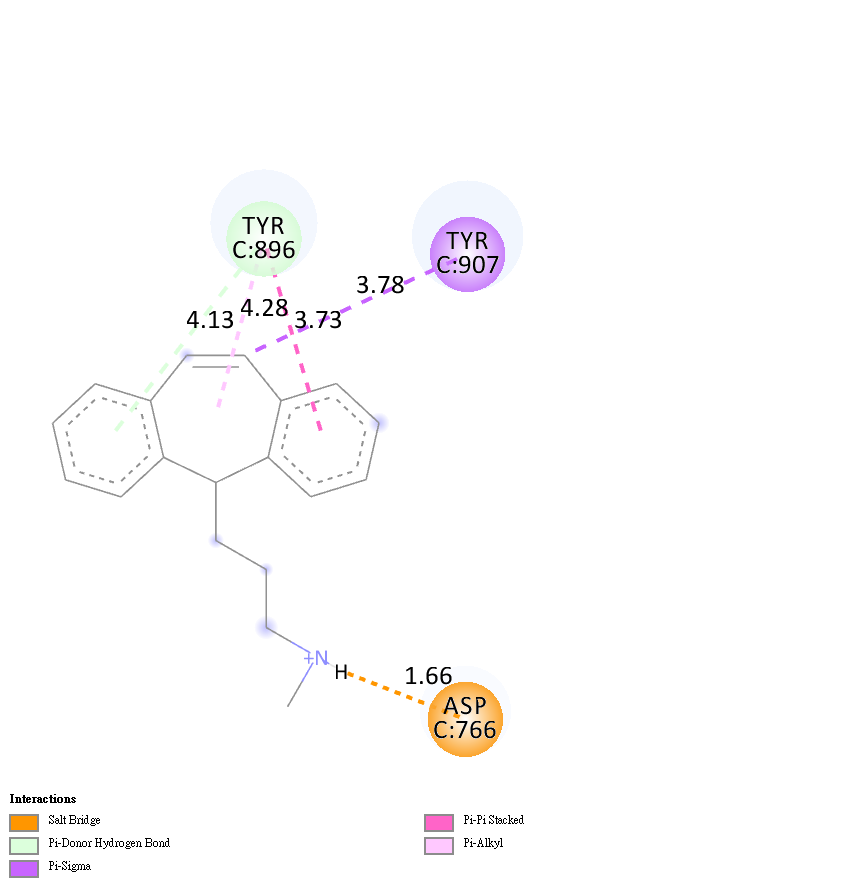

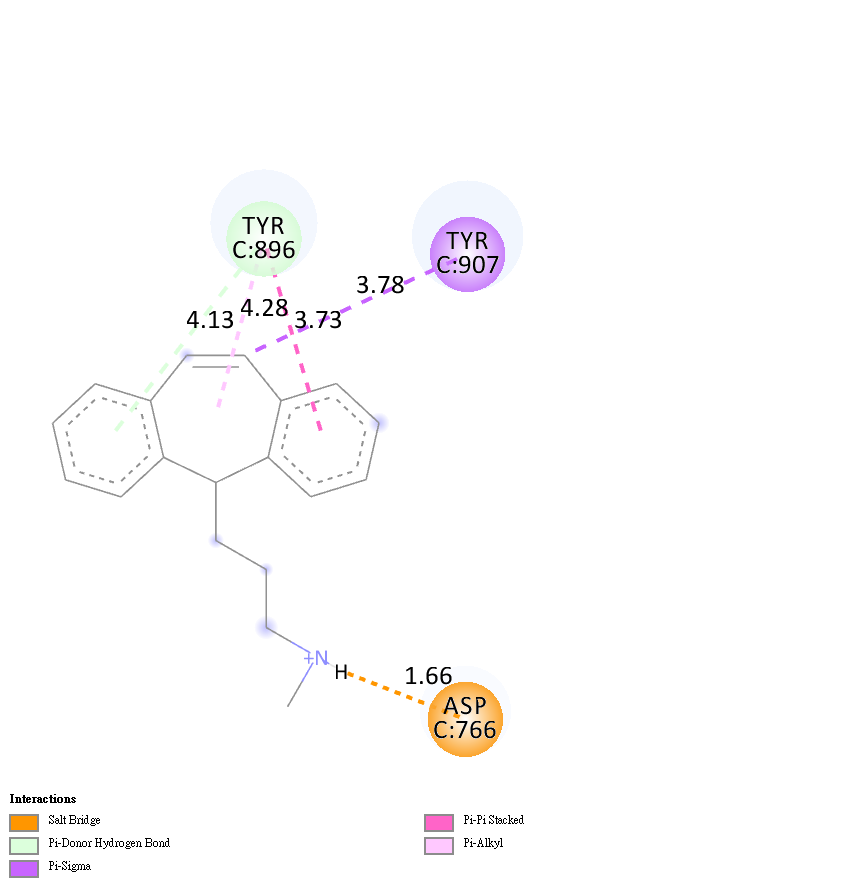

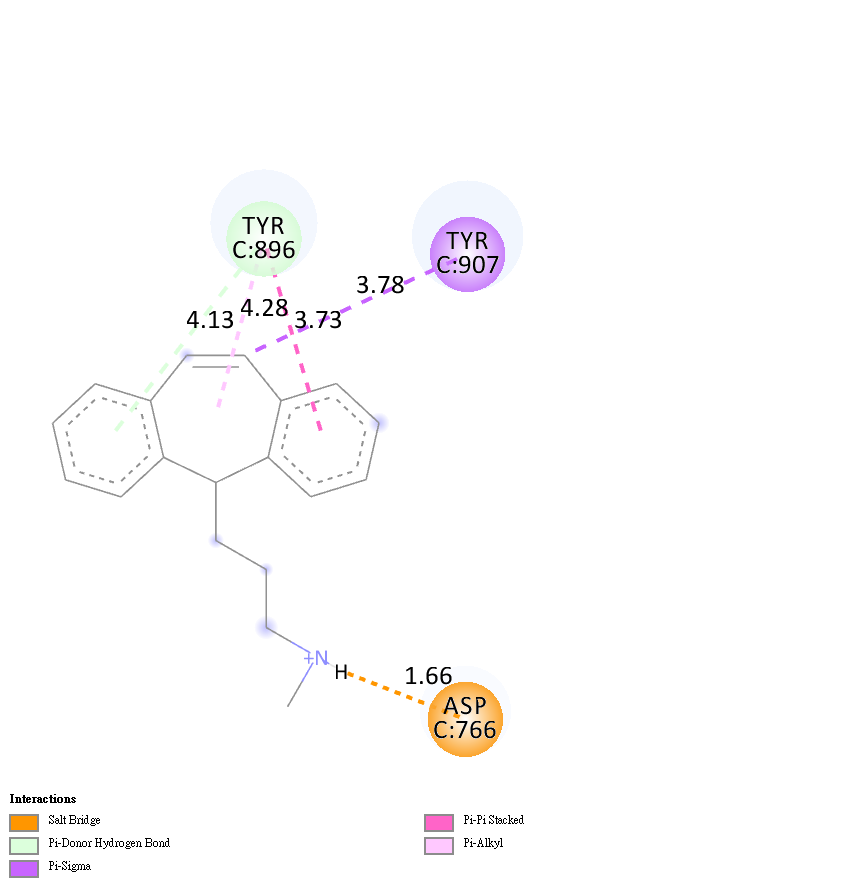

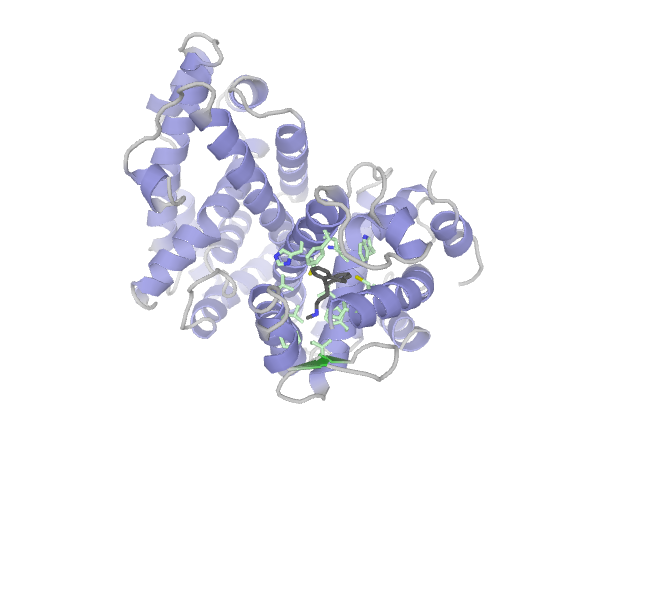

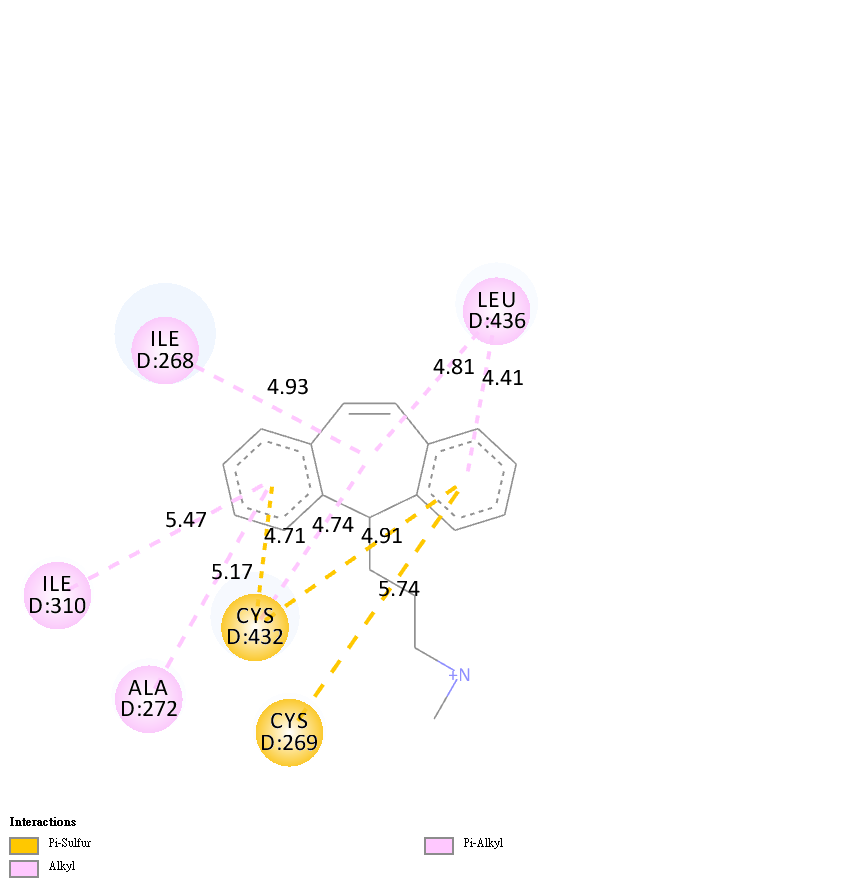

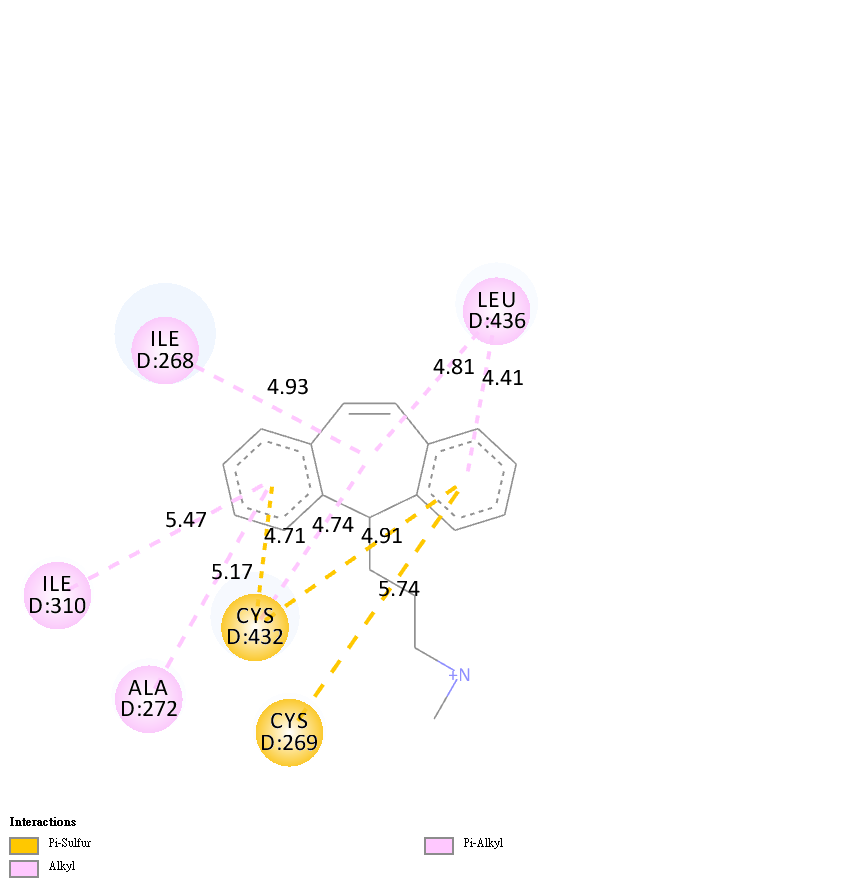

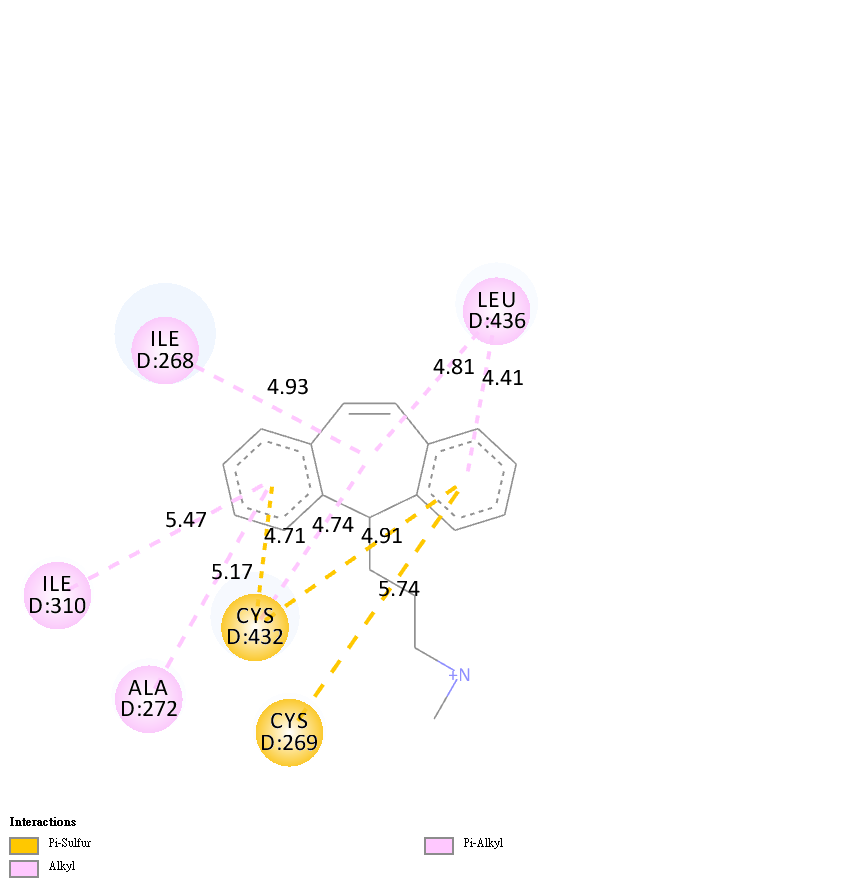


(Q) (R)


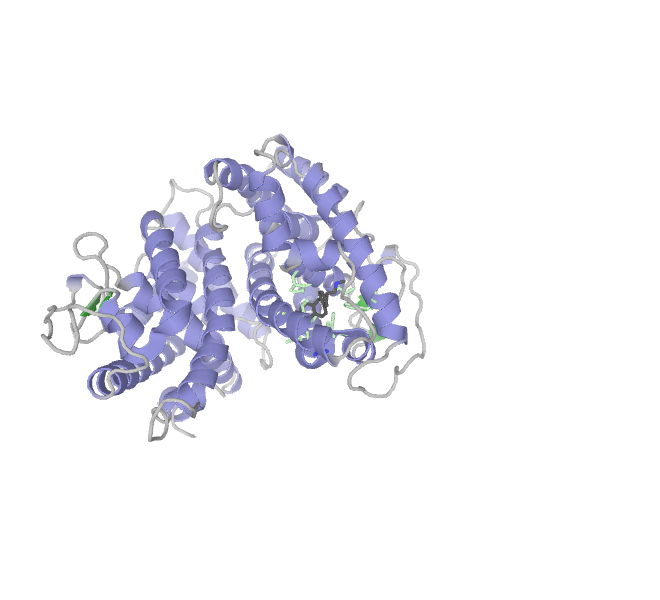

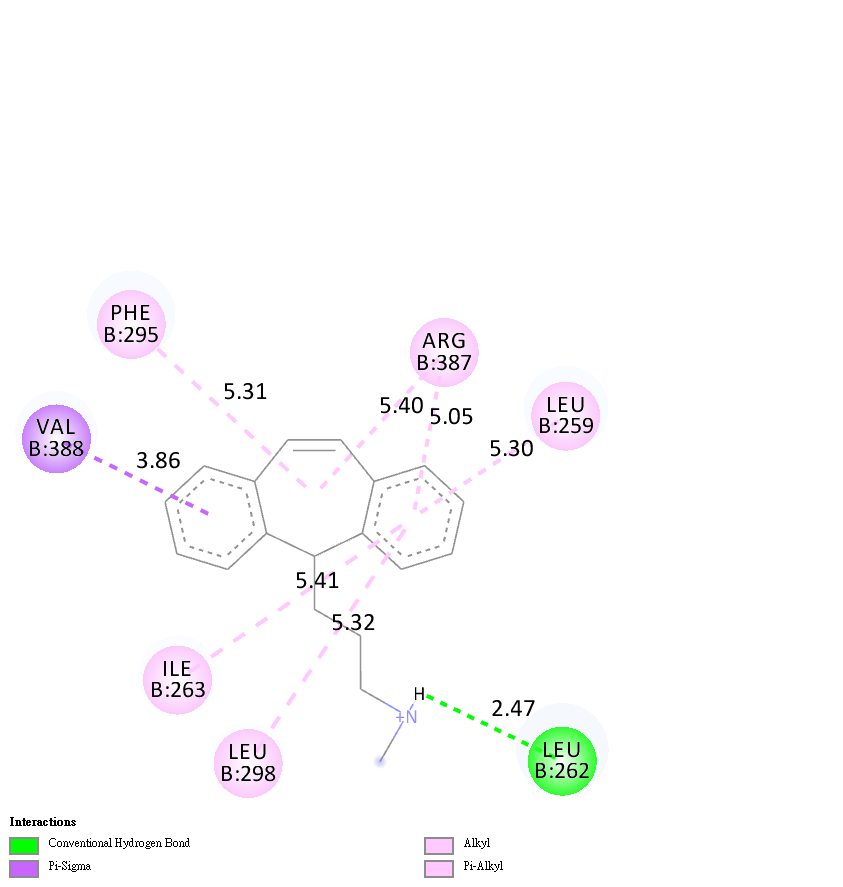

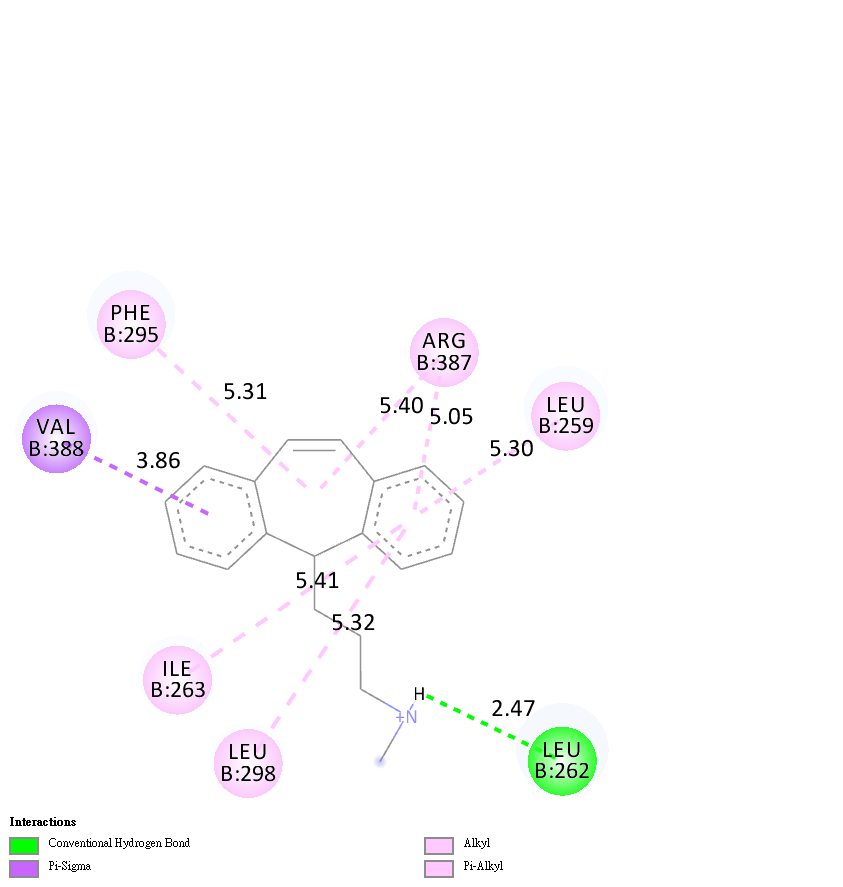

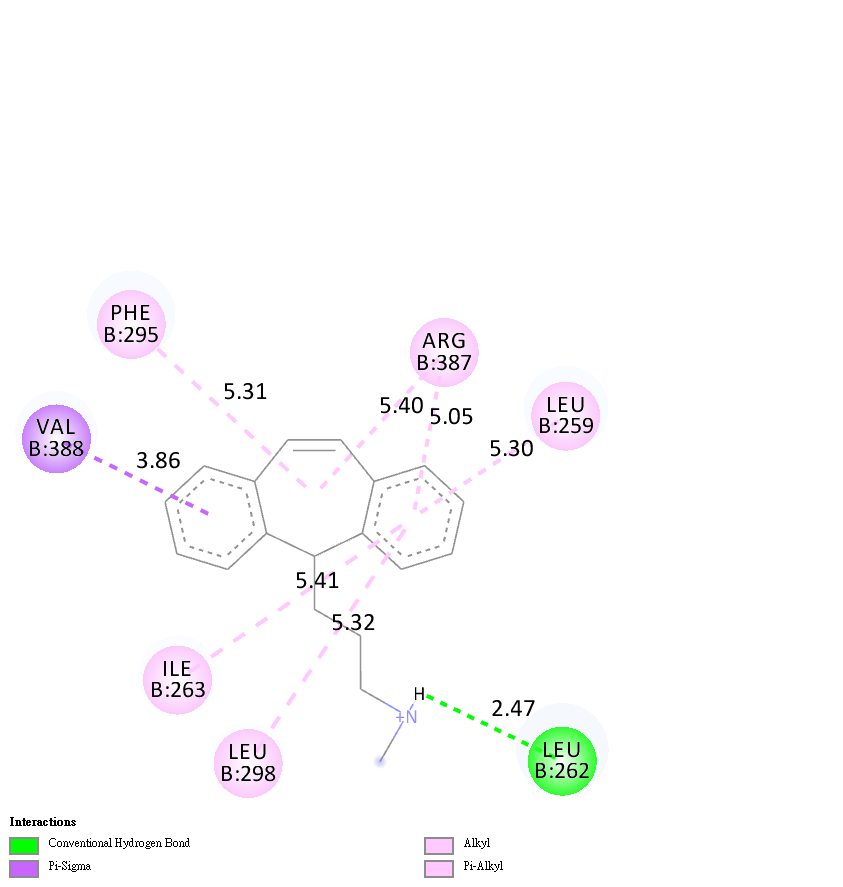

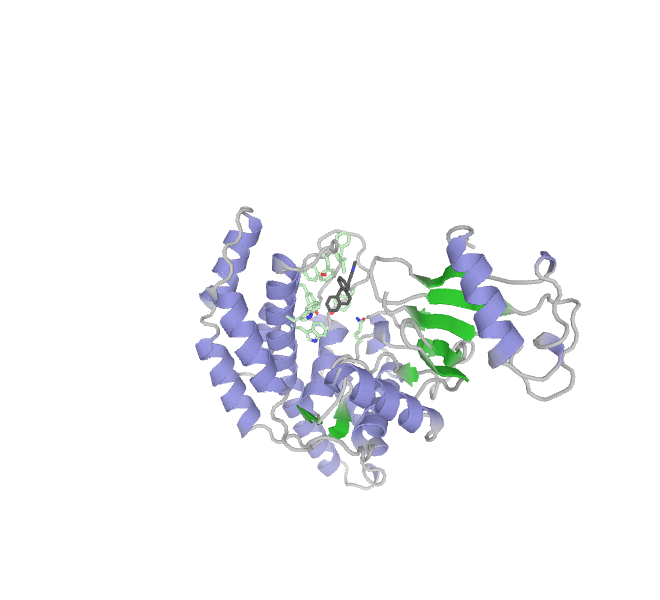

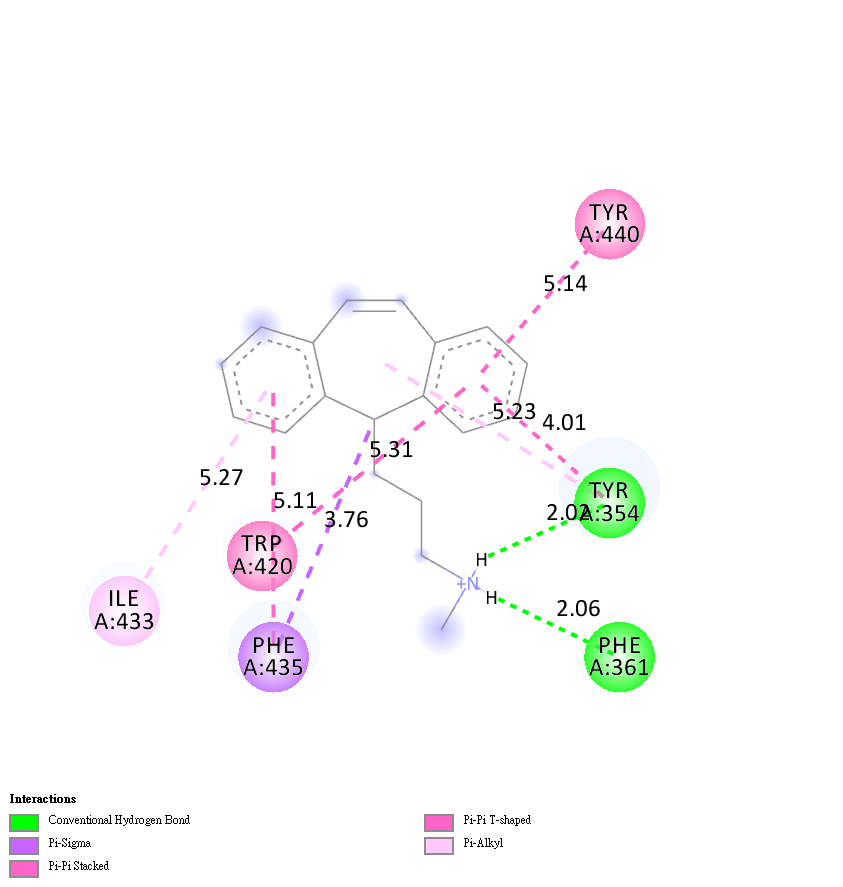

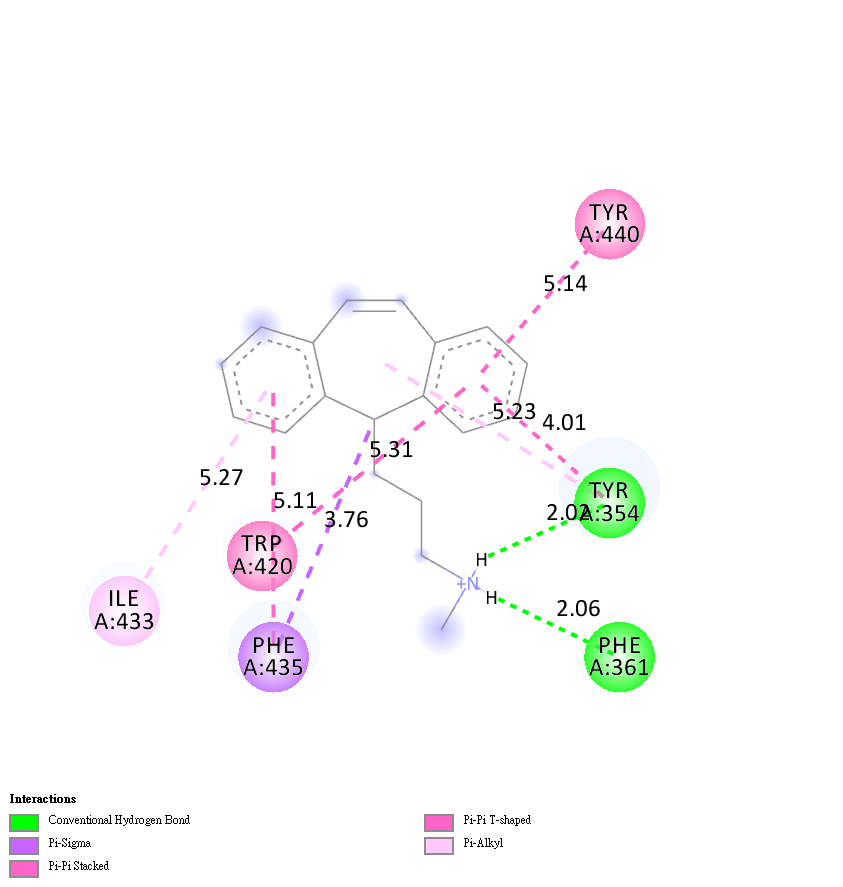

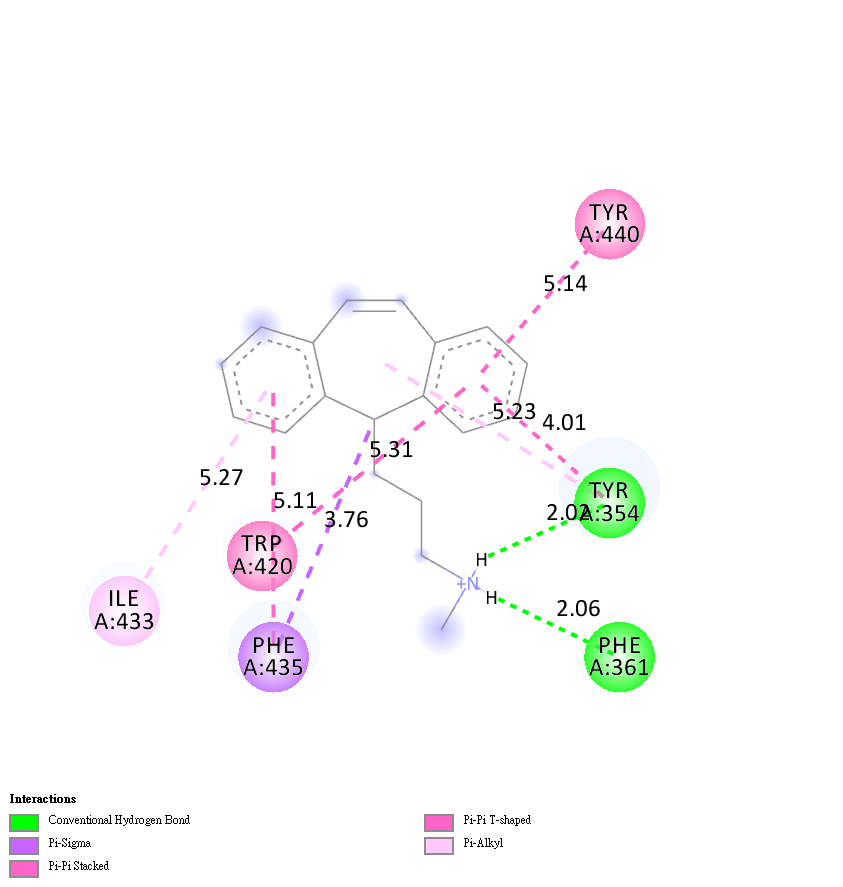


(S) (T)


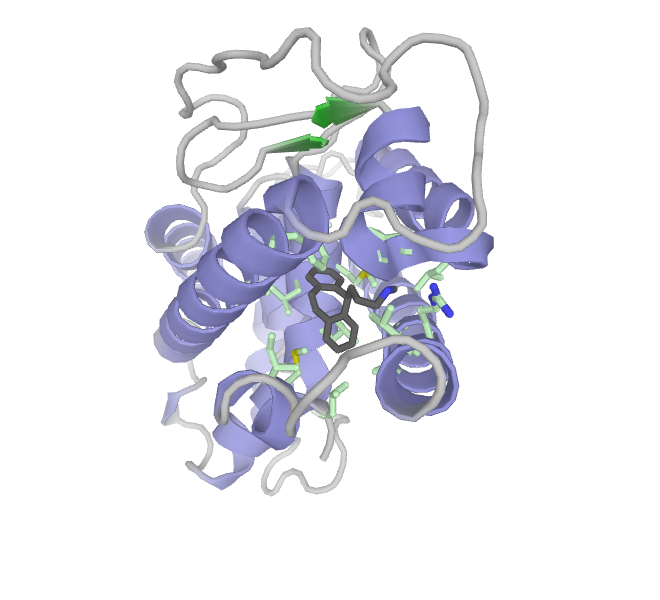

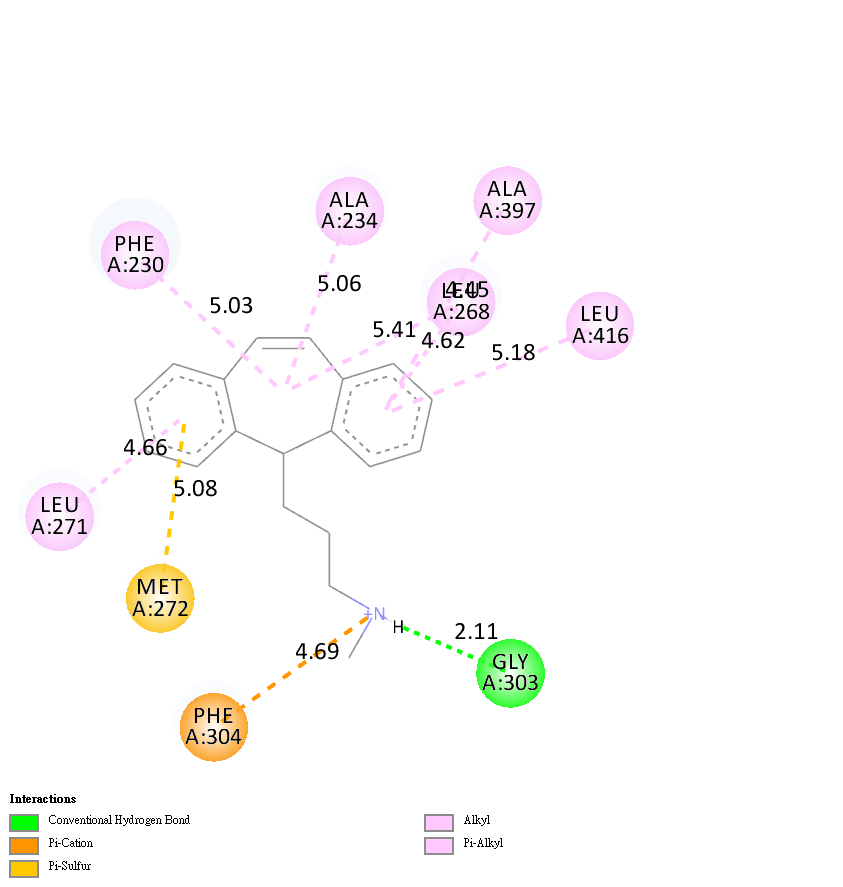

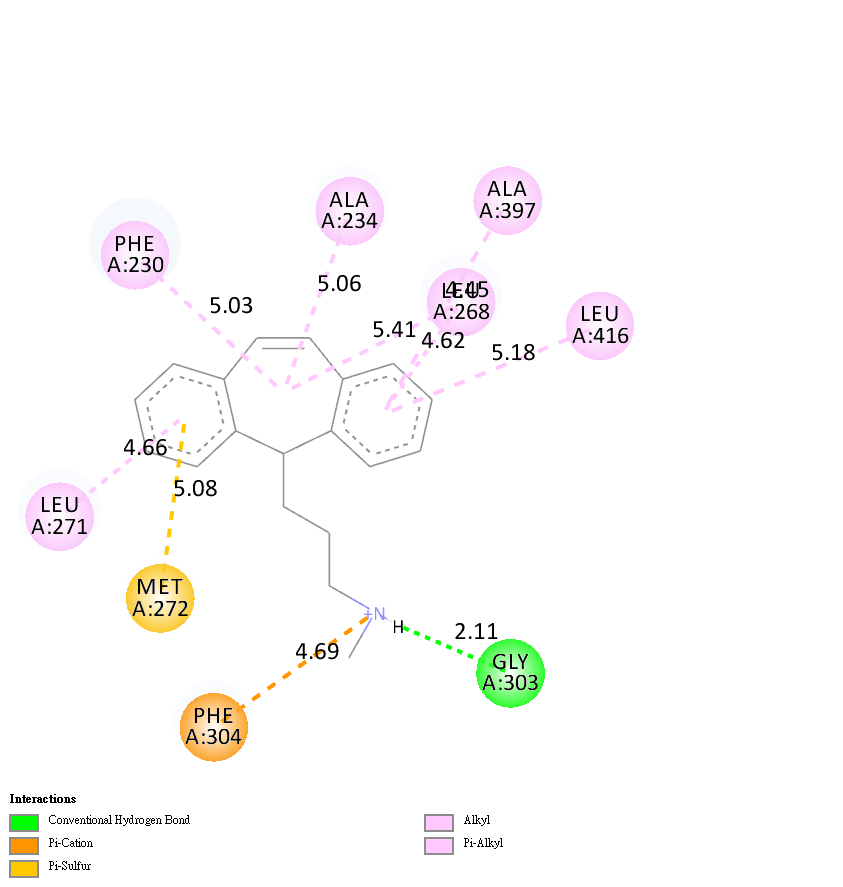

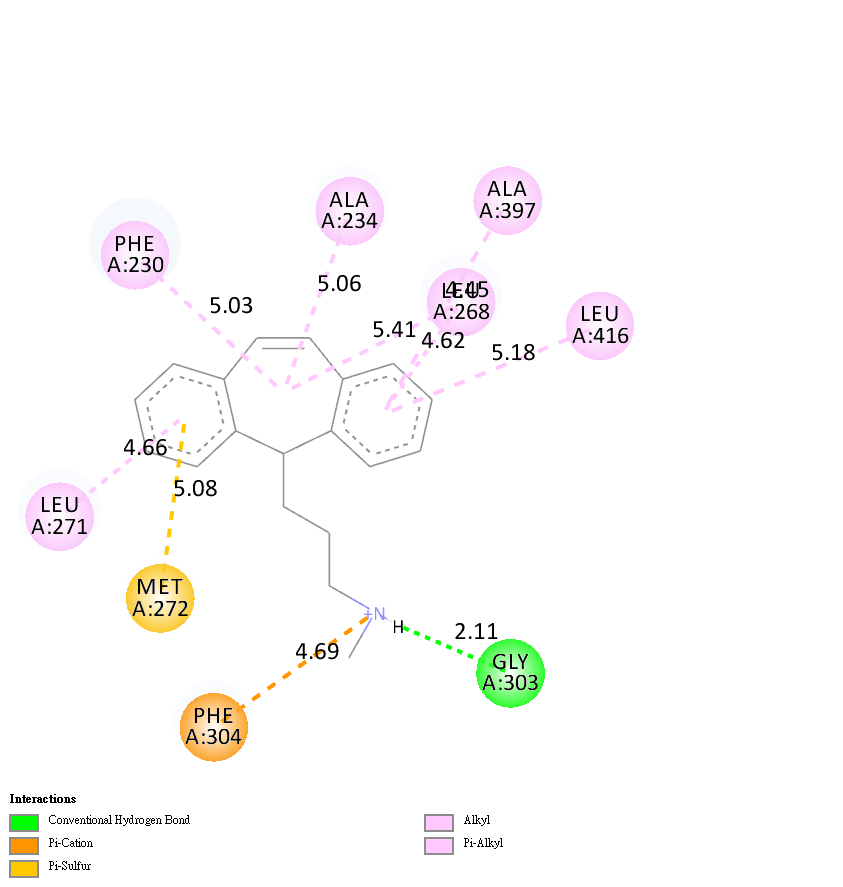

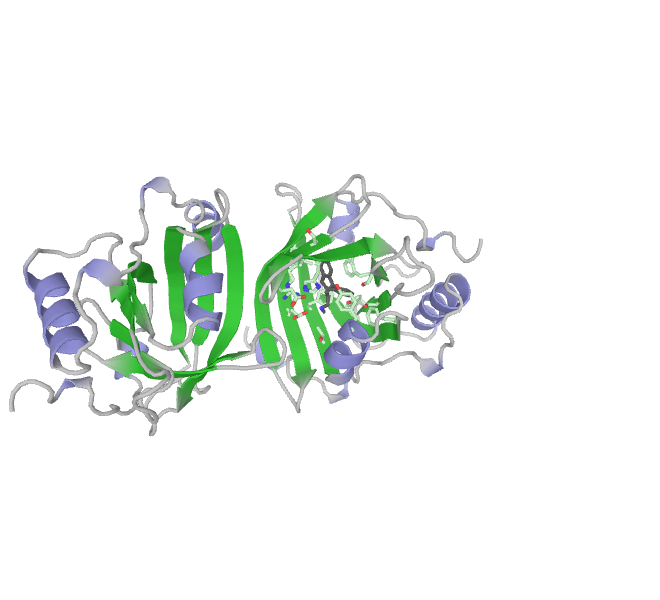

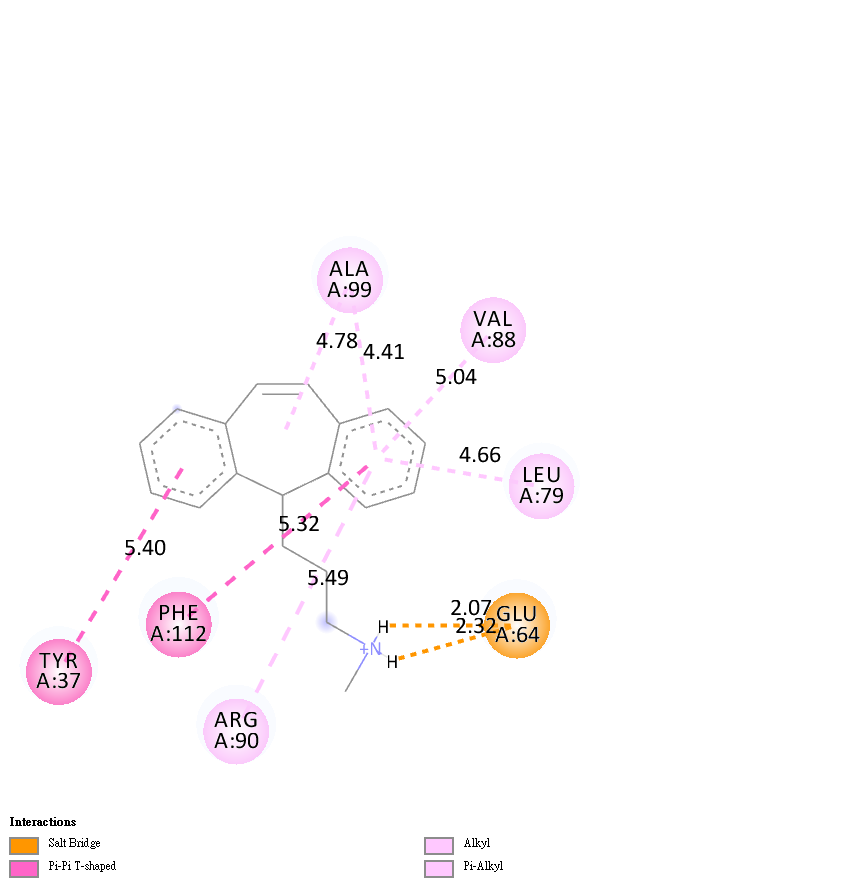

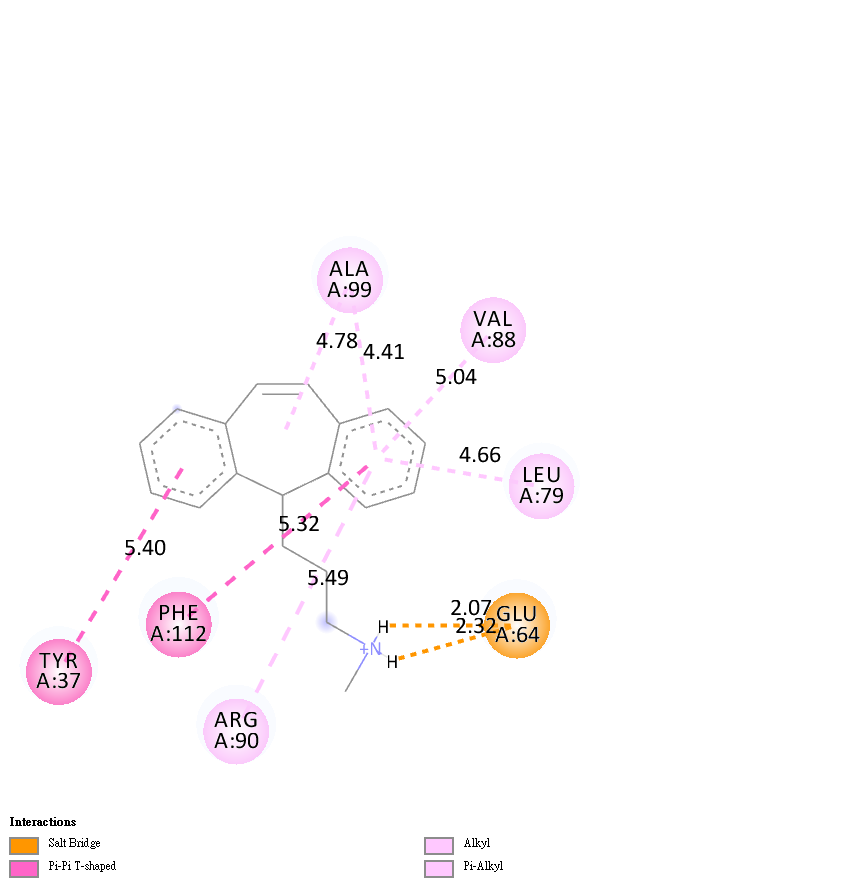

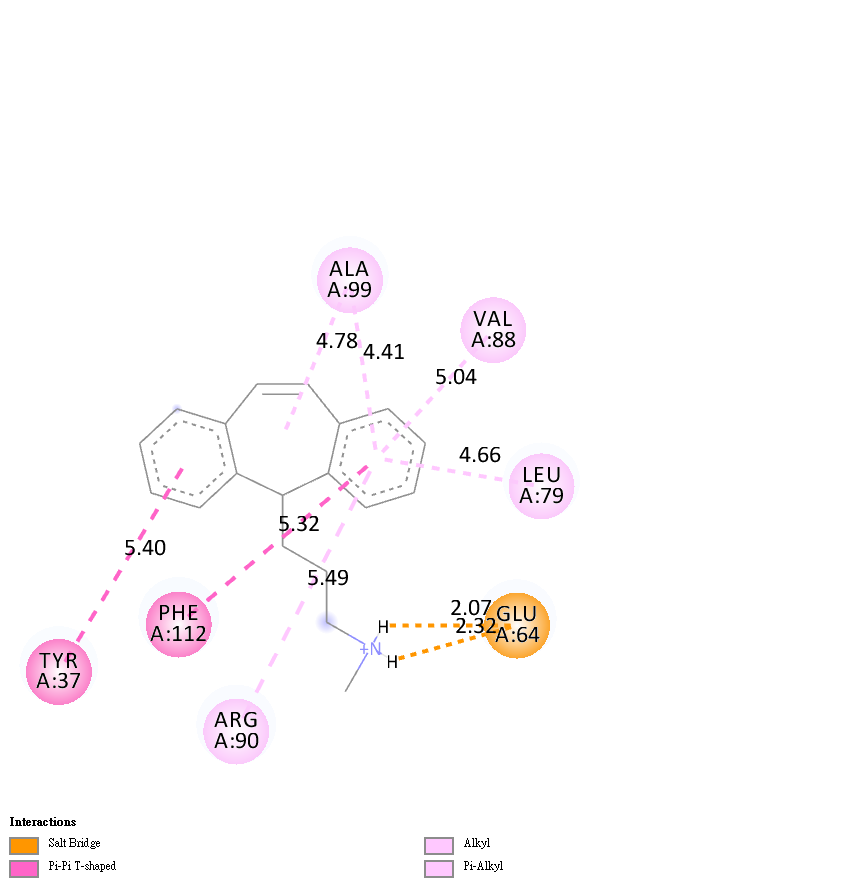


Figure S4. Binding pose of candidate drugs and predicted proteins

(A-J) Pose of Norcyclobenzaprine binding with PARP1, PARP2, ORM2, RBP1, SETD7, ESRRG, PGR, ESR1, NCOA2 and RARB, respectively. (K-T) Pose of Protriptyline binding with RBP1, PGR, NCOA2, PARP2, PARP1, RXRA, RARB, CHKA, RARG and ORM2, respectively.

(A)


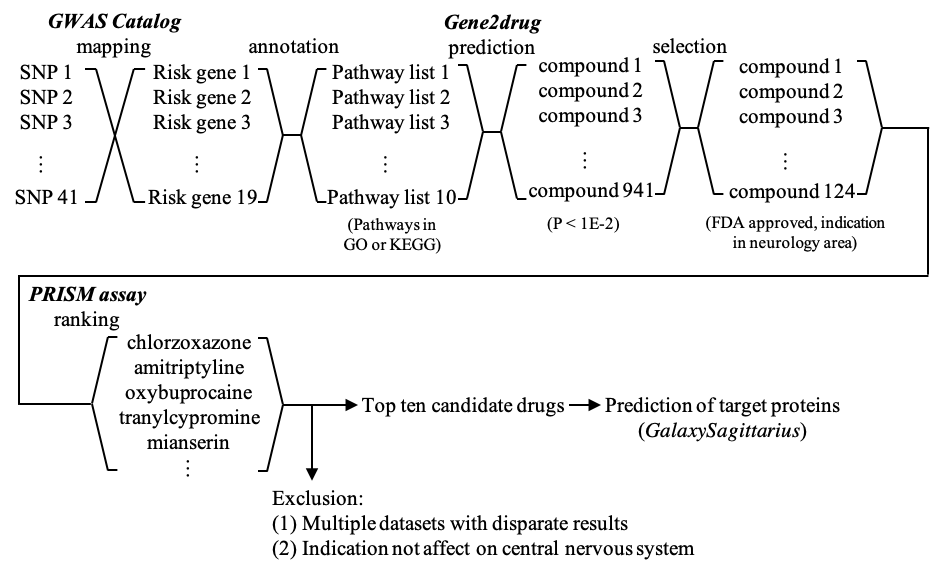


(B)

(C)

(D)

|  | ***Mean sensitivity*** |  | ***95% CI*** | |  | ***P-value*** |
| --- | --- | --- | --- | --- | --- | --- |
| Temozolomide | -0.197 |  | -0.340 | -0.053 |  | Ref. |
| Amitriptyline | 0.362 |  | 0.227 | 0.496 |  | 3.31E-07 |
| Tranylcypromine | 0.255 |  | 0.143 | 0.367 |  | 6.33E-06 |
| Mianserin | 0.230 |  | 0.131 | 0.330 |  | 8.86E-06 |
| Triflupromazine | 0.218 |  | 0.107 | 0.329 |  | 2.63E-05 |
| Doxylamine | 0.195 |  | 0.091 | 0.300 |  | 4.72E-05 |
| Protriptyline | 0.180 |  | 0.078 | 0.282 |  | 7.68E-05 |
| Metixene | 0.173 |  | 0.056 | 0.289 |  | 1.92E-04 |
| Citalopram | 0.167 |  | 0.080 | 0.255 |  | 7.04E-05 |
| Benserazide | 0.163 |  | 0.017 | 0.308 |  | 9.27E-04 |
| Acepromazine | 0.160 |  | 0.040 | 0.280 |  | 3.47E-04 |

(E)

| ***Predicted target proteins*** | ***Candidate drugs*** | ***Count*** |
| --- | --- | --- |
| PGR | acepromazine amitriptyline benserazide citalopram doxylamine metixene mianserin protriptyline triflupromazine | 9 |
| AR | acepromazine benserazide citalopram doxylamine metixene mianserin protriptyline triflupromazine | 8 |
| CHKA | benserazide citalopram doxylamine mianserin protriptyline | 4 |
| ESR1 | benserazide citalopram mianserin triflupromazine | 4 |
| ADPRT2 | acepromazine amitriptyline doxylamine | 3 |
| AGP2 | acepromazine amitriptyline triflupromazine | 3 |
| CHRNA1 | benserazide citalopram doxylamine | 3 |
| CHRNA7 | benserazide citalopram metixene | 3 |
| RARB | amitriptyline metixene protriptyline | 3 |
| RBP1 | amitriptyline metixene protriptyline | 3 |
| RORC | doxylamine mianserin tranylcypromine | 3 |
| ADPRT | acepromazine protriptyline | 2 |
| BRD4 | acepromazine mianserin | 2 |
| CREBBP | acepromazine doxylamine | 2 |
| MEN1 | benserazide citalopram | 2 |
| NR3C1 | acepromazine metixene | 2 |
| NR3C2 | acepromazine triflupromazine | 2 |
| NR5A2 | metixene protriptyline | 2 |
| PIM1 | acepromazine triflupromazine | 2 |
| RXRA | benserazide citalopram | 2 |
| SLC6A4 | benserazide citalopram | 2 |
| Proteins predicted to bind with only one drugs were not shown. | | |

Figure S5. The flow chart and briefly result of the directly screening protocol

(A) The flow chart of directly screening protocol. (B) The mean sensitivity, standard error (SD) and P-value of all the neurological drugs. (C-D) The sensitivity of top ten candidate drugs on 42 glioma cell lines ranking by mean sensitivity. Glioma cells show significantly higher sensitivity to top ten candidate drugs (×: mean value, CI: Confidence Interval). (E) The predicted targets of top ten candidate drugs by GalaxySagittarius.

Table S1. Forty-one risky SNPs associated with GBM and their mapped genes as reported by *GWAS Catalog*.

| ***Risk variant*** | ***P-value*** | ***OR*** | ***CI*** | ***Mapped gene*** | ***Reference*** |
| --- | --- | --- | --- | --- | --- |
| rs10069690 | 8.00E-74 | 1.61 | 1.53-1.69 | TERT | Melin BS, et al.  Nat Genet, 2017. |
| rs2297440 | 4.00E-46 | 1.48 | 1.40-1.56 | RTEL1-TNFRSF6B |  |
| rs634537 | 7.00E-45 | 1.37 | 1.31-1.43 | CDKN2B-AS1 |  |
| rs75061358 | 5.00E-34 | 1.63 | 1.50-1.76 | SEC61G-DT - NA |  |
| rs78378222 | 5.00E-29 | 2.63 | 2.22-3.11 | TP53 |  |
| rs723527 | 5.00E-23 | 1.25 | 1.20-1.31 | EGFR |  |
| rs10852606 | 1.00E-11 | 1.18 | 1.13-1.24 | HEATR3 |  |
| rs2235573 | 2.00E-10 | 1.15 | 1.10-1.20 | SLC16A8 |  |
| rs11233250 | 1.00E-09 | 1.24 | 1.16-1.33 | MIR4300HG |  |
| rs12752552 | 2.00E-09 | 1.22 | 1.15-1.31 | RAVER2 |  |
| rs2562152 | 2.00E-08 | 1.21 | 1.13-1.29 | RHBDF1 |  |
| rs55705857 | 9.00E-07 | 1.27 | 1.16-1.40 | CCDC26 |  |
| rs3751667 | 6.00E-06 | 1.13 | 1.07-1.19 | LMF1 |  |
| rs10069690 | 4.00E-33  5.00E-31  4.00E-18 | 1.78  1.72  1.51 | 1.62-1.95  1.57-1.89  1.38-1.66 | TERT | Ostrom QT et al.  Int J Cancer, 2018. |
| rs634537 | 4.00E-19 | 1.44 | 1.33-1.56 | CDKN2B-AS1 |  |
| rs2297440 | 7.00E-19 | 1.55 | 1.40-1.70 | RTEL1-TNFRSF6B |  |
| rs634537 | 1.00E-16 | 1.40 | 1.29-1.51 | CDKN2B-AS1 |  |
| rs75061358 | 4.00E-16 | 1.85 | 1.60-2.15 | SEC61G-DT - NA |  |
| rs78378222 | 9.00E-16  4.00E-13  3.00E-08 | 3.80  3.26  2.54 | 2.74-5.25  2.37-4.48  1.83-3.53 | TP53 |  |
| rs634537 | 3.00E-15 | 1.38 | 1.27-1.49 | CDKN2B-AS1 |  |
| rs2297440 | 1.00E-14  8.00E-13 | 1.45  1.41 | 1.32-1.60  1.28-1.55 | RTEL1-TNFRSF6B |  |
| rs75061358 | 2.00E-12  7.00E-12 | 1.70  1.67 | 1.47-1.97  1.44-1.94 | SEC61G-DT - NA |  |
| rs723527 | 2.00E-11  2.00E-09 | 1.32  1.28 | 1.21-1.43  1.18-1.39 | EGFR |  |
| rs55705857 | 9.00E-11 | 1.76 | 1.49-2.10 | CCDC26 |  |
| rs11979158 | 2.00E-10  6.00E-08 | 1.42  1.35 | 1.27-1.58  1.21-1.50 | EGFR |  |
| rs72709458 | 6.00E-24 | 1.68 | 1.52-1.86 | TERT | Kinnersley B et al.  Nat Commun, 2015. |
| rs6062302 | 1.00E-13 | 1.43 | 1.3-1.56 | RTEL1-TNFRSF6B |  |
| rs35850753 | 4.00E-12 | 2.79 | 2.09-3.72 | TP53 |  |
| rs145929329 | 5.00E-12 | 1.35 | 1.24-1.47 | CDKN2B-AS1 |  |
| rs75061358 | 6.00E-11 | 1.59 | 1.39-1.83 | SEC61G-DT - NA |  |
| rs3851634 | 3.00E-09 | 1.23 | 1.15-1.32 | POLR3B |  |
| rs59060240 | 4.00E-09 | 1.41 | 1.25-1.59 | EGFR |  |
| rs7732320 | 1.00E-06 | 1.64 | 1.34-2.00 | SSBP2 | Xiao Y et al,  Clin Cancer Res, 2012. |

Table S2. Pathways selected for *in-silico* screening.

| ***Risk Gene*** | ***Gene Ontology*** | ***KEGG pathways*** |
| --- | --- | --- |
| CCDC26 | NA | NA |
| CDKN2B-AS1 | NA | NA |
| EGFR | neurotrophin TRK receptor signaling pathway (GO-BP) epidermal growth factor receptor signaling pathway (GO-BP) fibroblast growth factor receptor signaling pathway (GO-BP) phosphatidylinositol-mediated signaling (GO-BP) positive regulation of cell proliferation (GO-BP) negative regulation of apoptotic process (GO-BP) innate immune response (GO-BP) Fc-epsilon receptor signaling pathway (GO-BP) cell morphogenesis (GO-BP) cell-cell adhesion (GO-BP) regulation of cell proliferation (GO-BP) cell proliferation (GO-BP) response to stress (GO-BP) regulation of nitric-oxide synthase activity (GO-BP) circadian rhythm (GO-BP) negative regulation of mitotic cell cycle (GO-BP) embryonic placenta development (GO-BP) protein phosphorylation (GO-BP) epidermis development (GO-BP) positive regulation of epithelial cell proliferation (GO-BP) translation (GO-BP) hair follicle development (GO-BP) positive regulation of MAP kinase activity (GO-BP) cell surface receptor signaling pathway (GO-BP) neuron projection morphogenesis (GO-BP) ossification (GO-BP) axon guidance (GO-BP) response to estradiol (GO-BP) salivary gland morphogenesis (GO-BP) lung development (GO-BP) protein autophosphorylation (GO-BP) peptidyl-tyrosine phosphorylation (GO-BP) response to oxidative stress (GO-BP) positive regulation of catenin import into nucleus (GO-BP) positive regulation of inflammatory response (GO-BP) response to organic cyclic compound (GO-BP) positive regulation of vasoconstriction (GO-BP) positive regulation of phosphorylation (GO-BP) positive regulation of ERK1 and ERK2 cascade (GO-BP) positive regulation of fibroblast proliferation (GO-BP) positive regulation of protein phosphorylation (GO-BP) liver development (GO-BP) positive regulation of vasodilation (GO-BP) transmembrane receptor protein tyrosine kinase signaling pathway (GO-BP) MAPK cascade (GO-BP) positive regulation of superoxide anion generation (GO-BP) cellular response to estradiol stimulus (GO-BP) positive regulation of cell migration (GO-BP) activation of phospholipase C activity (GO-BP) cellular response to mechanical stimulus (GO-BP) positive regulation of synaptic transmission, glutamatergic (GO-BP) positive regulation of nitric oxide biosynthetic process (GO-BP) activation of MAPKK activity (GO-BP) ovulation cycle (GO-BP) response to calcium ion (GO-BP) cellular response to drug (GO-BP) negative regulation of protein catabolic process (GO-BP) cellular response to growth factor stimulus (GO-BP) digestive tract morphogenesis (GO-BP) positive regulation of DNA replication (GO-BP) learning or memory (GO-BP) positive regulation of protein kinase B signaling (GO-BP) positive regulation of smooth muscle cell proliferation (GO-BP) negative regulation of epidermal growth factor receptor signaling pathway (GO-BP) response to osmotic stress (GO-BP) polysaccharide metabolic process (GO-BP) cellular response to epidermal growth factor stimulus (GO-BP) response to lipid (GO-BP) cellular response to dexamethasone stimulus (GO-BP) positive regulation of DNA repair (GO-BP) tongue development (GO-BP) cerebral cortex cell migration (GO-BP) regulation of peptidyl-tyrosine phosphorylation (GO-BP) positive regulation of cyclin-dependent protein serine/threonine kinase activity involved in G1/S transition of mitotic cell cycle (GO-BP) diterpenoid metabolic process (GO-BP) protein phosphatase binding (GO-MF) protein kinase binding (GO-MF) kinase activity (GO-MF) chromatin binding (GO-MF) signal transducer activity (GO-MF) receptor binding (GO-MF) enzyme binding (GO-MF) identical protein binding (GO-MF) integrin binding (GO-MF) protein heterodimerization activity (GO-MF) actin filament binding (GO-MF) transferase activity, transferring phosphorus-containing groups (GO-MF) double-stranded DNA binding (GO-MF) protein tyrosine kinase activity (GO-MF) protein kinase activity (GO-MF) transmembrane signaling receptor activity (GO-MF) glycoprotein binding (GO-MF) transmembrane receptor protein tyrosine kinase activity (GO-MF) receptor signaling protein tyrosine kinase activity (GO-MF) MAP kinase kinase kinase activity (GO-MF) nitric-oxide synthase regulator activity (GO-MF) basolateral plasma membrane (GO-CC) perinuclear region of cytoplasm (GO-CC) endocytic vesicle (GO-CC) nuclear membrane (GO-CC) membrane raft (GO-CC) cell surface (GO-CC) endoplasmic reticulum membrane (GO-CC) Golgi membrane (GO-CC) endosome membrane (GO-CC) endosome (GO-CC) apical plasma membrane (GO-CC) AP-2 adaptor complex (GO-CC) | MAPK SIGNALING PATHWAY (KEGG) ERBB SIGNALING PATHWAY (KEGG) CALCIUM SIGNALING PATHWAY (KEGG) CYTOKINE CYTOKINE RECEPTOR INTERACTION (KEGG) ENDOCYTOSIS (KEGG) DORSO VENTRAL AXIS FORMATION (KEGG) FOCAL ADHESION (KEGG) ADHERENS JUNCTION (KEGG) GAP JUNCTION (KEGG) REGULATION OF ACTIN CYTOSKELETON (KEGG) GNRH SIGNALING PATHWAY (KEGG) EPITHELIAL CELL SIGNALING IN HELICOBACTER PYLORI INFECTION (KEGG) PATHWAYS IN CANCER (KEGG) PANCREATIC CANCER (KEGG) ENDOMETRIAL CANCER (KEGG) GLIOMA (KEGG) PROSTATE CANCER (KEGG) MELANOMA (KEGG) BLADDER CANCER (KEGG) NON SMALL CELL LUNG CANCER (KEGG) |
| HEATR3 | NA | NA |
| LMF1 | protein secretion (GO-BP) ER to Golgi vesicle-mediated transport (GO-BP) regulation of cholesterol metabolic process (GO-BP) protein maturation (GO-BP) positive regulation of lipoprotein lipase activity (GO-BP) chylomicron remnant clearance (GO-BP) endoplasmic reticulum membrane (GO-CC) | NA |
| MIR4300HG | NA | NA |
| POLR3B | innate immune response (GO-BP) positive regulation of type I interferon production (GO-BP) defense response to virus (GO-BP) positive regulation of interferon-beta production (GO-BP) termination of RNA polymerase III transcription (GO-BP) transcription elongation from RNA polymerase III promoter (GO-BP) transcription from RNA polymerase III promoter (GO-BP) positive regulation of innate immune response (GO-BP) DNA-directed RNA polymerase activity (GO-MF) RNA polymerase III activity (GO-MF) DNA-directed RNA polymerase III complex (GO-CC) | PURINE METABOLISM (KEGG) PYRIMIDINE METABOLISM (KEGG) RNA POLYMERASE (KEGG) CYTOSOLIC DNA SENSING PATHWAY (KEGG) |
| RAVER2 | NA | NA |
| RHBDF1 | cell migration (GO-BP) protein transport (GO-BP) cell proliferation (GO-BP) proteolysis (GO-BP) regulation of protein secretion (GO-BP) regulation of proteasomal protein catabolic process (GO-BP) negative regulation of protein secretion (GO-BP) serine-type endopeptidase activity (GO-MF) growth factor binding (GO-MF) endoplasmic reticulum membrane (GO-CC) | NA |
| RTEL1-TNFRSF6B | nucleobase-containing compound metabolic process (GO-BP) ATP-dependent helicase activity (GO-MF) hydrolase activity, acting on acid anhydrides (GO-MF) hydrolase activity, acting on acid anhydrides, in phosphorus-containing anhydrides (GO-MF) ATP-dependent DNA helicase activity (GO-MF) | NA |
| SEC61G-DT | NA | NA |
| SLC16A8 | leukocyte migration (GO-BP) blood coagulation (GO-BP) cellular metabolic process (GO-BP) organic anion transport (GO-BP) pyruvate metabolic process (GO-BP) symporter activity (GO-MF) secondary active monocarboxylate transmembrane transporter activity (GO-MF) | NA |
| SSBP2 | single-stranded DNA binding (GO-MF) | NA |
| TERT | telomere maintenance (GO-BP) telomere maintenance via telomerase (GO-BP) negative regulation of extrinsic apoptotic signaling pathway in absence of ligand (GO-BP) replicative senescence (GO-BP) protein homodimerization activity (GO-MF) RNA binding (GO-MF) telomeric DNA binding (GO-MF) nucleolus (GO-CC) PML body (GO-CC) chromosome, telomeric region (GO-CC) telomerase holoenzyme complex (GO-CC) nuclear telomere cap complex (GO-CC) | NA |
| TP53 | negative regulation of apoptotic process (GO-BP) protein localization (GO-BP) gastrulation (GO-BP) regulation of cell proliferation (GO-BP) regulation of mitochondrial membrane permeability (GO-BP) multicellular organismal development (GO-BP) cell differentiation (GO-BP) modulation by virus of host morphology or physiology (GO-BP) somitogenesis (GO-BP) in utero embryonic development (GO-BP) multicellular organism growth (GO-BP) regulation of apoptotic process (GO-BP) cell proliferation (GO-BP) negative regulation of transcription from RNA polymerase II promoter (GO-BP) negative regulation of transcription, DNA-dependent (GO-BP) positive regulation of transcription, DNA-dependent (GO-BP) protein complex assembly (GO-BP) intrinsic apoptotic signaling pathway by p53 class mediator (GO-BP) blood coagulation (GO-BP) negative regulation of cell proliferation (GO-BP) regulation of cell cycle (GO-BP) DNA strand renaturation (GO-BP) cellular response to ionizing radiation (GO-BP) response to X-ray (GO-BP) cellular response to DNA damage stimulus (GO-BP) chromosome organization (GO-BP) positive regulation of peptidyl-tyrosine phosphorylation (GO-BP) intrinsic apoptotic signaling pathway in response to DNA damage by p53 class mediator (GO-BP) mitotic G1 DNA damage checkpoint (GO-BP) cell cycle (GO-BP) transforming growth factor beta receptor signaling pathway (GO-BP) positive regulation of apoptotic process (GO-BP) negative regulation of transforming growth factor beta receptor signaling pathway (GO-BP) cellular response to hypoxia (GO-BP) cellular response to UV (GO-BP) double-strand break repair (GO-BP) neuron apoptotic process (GO-BP) response to oxidative stress (GO-BP) DNA damage response, signal transduction by p53 class mediator resulting in transcription of p21 class mediator (GO-BP) cell cycle arrest (GO-BP) DNA damage response, signal transduction by p53 class mediator resulting in cell cycle arrest (GO-BP) central nervous system development (GO-BP) response to antibiotic (GO-BP) negative regulation of cell growth (GO-BP) negative regulation of DNA replication (GO-BP) Ras protein signal transduction (GO-BP) ER overload response (GO-BP) cellular response to glucose starvation (GO-BP) embryonic organ development (GO-BP) cell aging (GO-BP) DNA damage response, signal transduction by p53 class mediator (GO-BP) positive regulation of cell cycle arrest (GO-BP) response to drug (GO-BP) cellular protein localization (GO-BP) positive regulation of cardiac muscle cell apoptotic process (GO-BP) regulation of neuron apoptotic process (GO-BP) response to UV (GO-BP) intrinsic apoptotic signaling pathway (GO-BP) positive regulation of release of cytochrome c from mitochondria (GO-BP) cellular response to drug (GO-BP) base-excision repair (GO-BP) nucleotide-excision repair (GO-BP) positive regulation of neuron apoptotic process (GO-BP) protein import into nucleus, translocation (GO-BP) response to salt stress (GO-BP) positive regulation of intrinsic apoptotic signaling pathway (GO-BP) T cell differentiation in thymus (GO-BP) positive regulation of protein oligomerization (GO-BP) determination of adult lifespan (GO-BP) mitotic cell cycle arrest (GO-BP) response to gamma radiation (GO-BP) positive regulation of reactive oxygen species metabolic process (GO-BP) release of cytochrome c from mitochondria (GO-BP) response to ischemia (GO-BP) rRNA transcription (GO-BP) negative regulation of reactive oxygen species metabolic process (GO-BP) negative regulation of neuroblast proliferation (GO-BP) positive regulation of histone deacetylation (GO-BP) protein tetramerization (GO-BP) negative regulation of fibroblast proliferation (GO-BP) embryo development ending in birth or egg hatching (GO-BP) replicative senescence (GO-BP) T cell proliferation involved in immune response (GO-BP) B cell lineage commitment (GO-BP) T cell lineage commitment (GO-BP) protein kinase binding (GO-MF) chromatin binding (GO-MF) enzyme binding (GO-MF) identical protein binding (GO-MF) ubiquitin protein ligase binding (GO-MF) protein heterodimerization activity (GO-MF) p53 binding (GO-MF) transcription factor binding (GO-MF) protease binding (GO-MF) RNA polymerase II core promoter proximal region sequence-specific DNA binding transcription factor activity involved in positive regulation of transcription (GO-MF) RNA polymerase II transcription regulatory region sequence-specific DNA binding transcription factor activity involved in positive regulation of transcription (GO-MF) transcription regulatory region DNA binding (GO-MF) receptor tyrosine kinase binding (GO-MF) protein N-terminus binding (GO-MF) chaperone binding (GO-MF) RNA polymerase II core promoter sequence-specific DNA binding (GO-MF) damaged DNA binding (GO-MF) copper ion binding (GO-MF) RNA polymerase II transcription factor binding (GO-MF) protein phosphatase 2A binding (GO-MF) histone acetyltransferase binding (GO-MF) MDM2/MDM4 family protein binding (GO-MF) nucleolus (GO-CC) protein complex (GO-CC) nuclear body (GO-CC) PML body (GO-CC) mitochondrial matrix (GO-CC) nuclear matrix (GO-CC) replication fork (GO-CC) nuclear chromatin (GO-CC) chromatin (GO-CC) transcription factor TFIID complex (GO-CC) | MAPK SIGNALING PATHWAY (KEGG) CELL CYCLE (KEGG) P53 SIGNALING PATHWAY (KEGG) APOPTOSIS (KEGG) WNT SIGNALING PATHWAY (KEGG) NEUROTROPHIN SIGNALING PATHWAY (KEGG) AMYOTROPHIC LATERAL SCLEROSIS ALS (KEGG) HUNTINGTONS DISEASE (KEGG) PATHWAYS IN CANCER (KEGG) COLORECTAL CANCER (KEGG) PANCREATIC CANCER (KEGG) ENDOMETRIAL CANCER (KEGG) GLIOMA (KEGG) PROSTATE CANCER (KEGG) THYROID CANCER (KEGG) BASAL CELL CARCINOMA (KEGG) MELANOMA (KEGG) BLADDER CANCER (KEGG) CHRONIC MYELOID LEUKEMIA (KEGG) SMALL CELL LUNG CANCER (KEGG) NON SMALL CELL LUNG CANCER (KEGG) |

Table S3. Compounds and lists of pathways to which the compounds belong.

| ***Compounds*** | ***Lists of pathways to which the compounds belong*** | ***Count*** |
| --- | --- | --- |
| geldanamycin | POLR3B RHBDF1 RTEL1-TNFRSF6B SSBP2 TERT TP53 | 6 |
| 0297417-0002B | EGFR POLR3B RTEL1-TNFRSF6B SLC16A8 TERT | 5 |
| napelline | EGFR POLR3B RTEL1-TNFRSF6B SSBP2 TP53 | 5 |
| butein | LMF1 RHBDF1 RTEL1-TNFRSF6B TERT TP53 | 5 |
| homochlorcyclizine | EGFR POLR3B RHBDF1 RTEL1-TNFRSF6B TERT | 5 |
| scriptaid | EGFR RHBDF1 RTEL1-TNFRSF6B SLC16A8 TP53 | 5 |
| thiostrepton | RHBDF1 RTEL1-TNFRSF6B SSBP2 TERT TP53 | 5 |
| imipramine | EGFR RHBDF1 RTEL1-TNFRSF6B SSBP2 TP53 | 5 |
| protriptyline | EGFR POLR3B RHBDF1 SSBP2 TP53 | 5 |
| nortriptyline | EGFR POLR3B RHBDF1 RTEL1-TNFRSF6B | 4 |
| haloperidol | EGFR RHBDF1 RTEL1-TNFRSF6B TERT | 4 |
| ciclosporin | EGFR RHBDF1 RTEL1-TNFRSF6B TP53 | 4 |
| trifluoperazine | EGFR POLR3B RHBDF1 TP53 | 4 |
| chlorprothixene | EGFR POLR3B TERT TP53 | 4 |
| mefloquine calmidazolium | EGFR POLR3B RHBDF1 RTEL1-TNFRSF6B | 4 |
| amiodarone | EGFR POLR3B RTEL1-TNFRSF6B TERT | 4 |
| mepacrine | EGFR POLR3B RTEL1-TNFRSF6B TP53 | 4 |
| raubasine | RHBDF1 RTEL1-TNFRSF6B SLC16A8 TERT | 4 |
| perphenazine | RHBDF1 RTEL1-TNFRSF6B TERT TP53 | 4 |
| tonzonium_bromide levomepromazine | EGFR RHBDF1 RTEL1-TNFRSF6B TERT | 4 |
| alimemazine spiperone C-75 | EGFR RHBDF1 RTEL1-TNFRSF6B TP53 | 4 |
| rescinnamine | RTEL1-TNFRSF6B SLC16A8 TERT TP53 | 4 |
| chloropyramine STOCK1N-35215 digitoxigenin corynanthine | EGFR RTEL1-TNFRSF6B TERT TP53 | 4 |
| melatonin | EGFR LMF1 TERT TP53 | 4 |
| iobenguane | EGFR POLR3B RHBDF1 TERT | 4 |
| loperamide verteporfin | EGFR POLR3B RHBDF1 TP53 | 4 |
| cytochalasin_B | POLR3B SLC16A8 TERT TP53 | 4 |
| sulfamethoxypyridazine | EGFR POLR3B SLC16A8 TP53 | 4 |
| solanine | EGFR POLR3B TERT TP53 | 4 |
| ionomycin | RHBDF1 SSBP2 TERT TP53 | 4 |
| norcyclobenzaprine | EGFR RHBDF1 SSBP2 TP53 | 4 |
| monensin | EGFR RHBDF1 TERT TP53 | 4 |
| cephaeline digoxigenin | EGFR SLC16A8 TERT TP53 | 4 |
| digoxin | EGFR SSBP2 TERT TP53 | 4 |

Table S4. Downstream pathways shared by the 11 candidate drugs.

| ***Rank*** | ***Pathway Name*** | ***E-Score*** | ***P value*** |
| --- | --- | --- | --- |
| 1 | mitotic nuclear envelope disassembly | -0.81 | 1.26E-06 |
| 2 | endoplasmic reticulum unfolded protein response | 0.78 | 3.39E-06 |
| 3 | mitotic cell cycle | -0.78 | 3.67E-06 |
| 4 | regulation of glucose transport | -0.78 | 4.07E-06 |
| 5 | cholesterol biosynthetic process | 0.77 | 4.40E-06 |
| 6 | isoprenoid biosynthetic process | 0.77 | 4.89E-06 |
| 7 | interferon-gamma-mediated signaling pathway | 0.75 | 9.02E-06 |
| 8 | positive regulation of ATPase activity | 0.75 | 1.08E-05 |
| 9 | ribosome assembly | -0.74 | 1.10E-05 |
| 10 | hexose transport | -0.74 | 1.19E-05 |
| 11 | response to endoplasmic reticulum stress | 0.74 | 1.45E-05 |
| 12 | platelet-derived growth factor receptor signaling pathway | 0.73 | 1.56E-05 |
| 13 | glucose transport | -0.73 | 1.60E-05 |
| 14 | negative regulation of mRNA splicing, via spliceosome | -0.73 | 1.72E-05 |
| 15 | autophagy | 0.73 | 1.72E-05 |
| 16 | activation of signaling protein activity involved in unfolded protein response | 0.73 | 2.00E-05 |
| 17 | DNA repair | -0.72 | 2.26E-05 |
| 18 | Wnt signaling pathway | 0.72 | 2.80E-05 |
| 19 | protein N-linked glycosylation via asparagine | 0.71 | 3.01E-05 |
| 20 | DNA replication | -0.71 | 3.09E-05 |
| 21 | DNA recombination | -0.71 | 3.16E-05 |
| 22 | positive regulation of BMP signaling pathway | 0.7 | 4.41E-05 |
| 23 | positive regulation of autophagy | 0.7 | 4.73E-05 |
| 24 | epidermal growth factor receptor signaling pathway | 0.7 | 4.96E-05 |
| 25 | post-Golgi vesicle-mediated transport | 0.7 | 5.20E-05 |
| 26 | sterol biosynthetic process | 0.7 | 5.20E-05 |
| 27 | retrograde transport, endosome to Golgi | 0.69 | 5.32E-05 |
| 28 | negative regulation of extrinsic apoptotic signaling pathway | 0.69 | 5.71E-05 |
| 29 | ribosome biogenesis | -0.69 | 5.98E-05 |
| 30 | response to organic substance | 0.69 | 6.56E-05 |
| 31 | vesicle-mediated transport | 0.68 | 7.53E-05 |
| 32 | positive regulation of p38MAPK cascade | 0.68 | 7.53E-05 |
| 33 | negative regulation of histone H3-K9 methylation | -0.68 | 7.70E-05 |
| 34 | cell-cell adhesion | 0.68 | 9.25E-05 |
| 35 | rRNA processing | -0.68 | 9.25E-05 |
| 36 | tRNA processing | -0.68 | 9.25E-05 |
| 37 | DNA replication initiation | -0.67 | 9.68E-05 |
| 38 | cellular response to antibiotic | 0.67 | 1.01E-04 |
| 39 | RNA splicing, via transesterification reactions | -0.67 | 1.01E-04 |
| 40 | negative regulation of cell proliferation | 0.67 | 1.08E-04 |
| 41 | DNA strand elongation involved in DNA replication | -0.67 | 1.16E-04 |
| 42 | mRNA processing | -0.67 | 1.27E-04 |
| 43 | RNA processing | -0.66 | 1.33E-04 |
| 44 | endocytosis | 0.66 | 1.33E-04 |
| 45 | nucleotide-excision repair, DNA gap filling | -0.66 | 1.45E-04 |
| 46 | sphingolipid metabolic process | 0.66 | 1.55E-04 |
| 47 | vasculature development | 0.66 | 1.62E-04 |
| 48 | mRNA export from nucleus | -0.66 | 1.69E-04 |
| 49 | G1/S transition of mitotic cell cycle | -0.65 | 1.73E-04 |
| 50 | extrinsic apoptotic signaling pathway in absence of ligand | 0.65 | 1.73E-04 |
| 51 | lysine catabolic process | -0.65 | 1.73E-04 |
| 52 | JNK cascade | 0.65 | 1.77E-04 |
| 53 | response to cholesterol | 0.65 | 1.81E-04 |
| 54 | smooth muscle cell migration | 0.65 | 1.81E-04 |
| 55 | mRNA 3'-end processing | -0.65 | 1.93E-04 |
| 56 | transcription-coupled nucleotide-excision repair | -0.65 | 2.20E-04 |
| 57 | fatty acid biosynthetic process | 0.64 | 2.30E-04 |
| 58 | cranial suture morphogenesis | 0.64 | 2.35E-04 |
| 59 | pre-miRNA processing | -0.64 | 2.62E-04 |
| 60 | RNA catabolic process | -0.64 | 2.73E-04 |
| 61 | regulation of transcription from RNA polymerase II promoter in response to hypoxia | 0.64 | 2.85E-04 |
| 62 | positive regulation of cell death | 0.64 | 2.91E-04 |
| 63 | response to hypoxia | 0.63 | 3.04E-04 |
| 64 | cellular response to starvation | 0.63 | 3.04E-04 |
| 65 | GTP catabolic process | 0.63 | 3.11E-04 |
| 66 | small GTPase mediated signal transduction | 0.63 | 3.11E-04 |
| 67 | RNA splicing | -0.63 | 3.17E-04 |
| 68 | decidualization | 0.63 | 3.31E-04 |
| 69 | mRNA splicing, via spliceosome | -0.63 | 3.38E-04 |
| 70 | mitochondrial DNA replication | -0.63 | 3.53E-04 |
| 71 | telomere maintenance | -0.63 | 3.68E-04 |
| 72 | cellular response to peptide hormone stimulus | 0.63 | 3.76E-04 |
| 73 | toll-like receptor 3 signaling pathway | 0.63 | 3.92E-04 |
| 74 | protein transport | 0.62 | 4.09E-04 |
| 75 | toll-like receptor signaling pathway | 0.62 | 4.09E-04 |
| 76 | telomere maintenance via semi-conservative replication | -0.62 | 4.09E-04 |
| 77 | positive regulation of neuron apoptotic process | 0.62 | 4.35E-04 |
| 78 | phospholipid metabolic process | 0.62 | 4.45E-04 |
| 79 | bone development | 0.62 | 4.45E-04 |
| 80 | transcription elongation from RNA polymerase II promoter | -0.62 | 4.45E-04 |
| 81 | regulation of lamellipodium assembly | 0.62 | 4.54E-04 |
| 82 | flavonoid metabolic process | 0.62 | 4.63E-04 |
| 83 | mitochondrial electron transport, NADH to ubiquinone | -0.62 | 4.73E-04 |
| 84 | DNA unwinding involved in DNA replication | -0.62 | 5.04E-04 |
| 85 | endosome organization | 0.61 | 5.47E-04 |
| 86 | nuclear-transcribed mRNA catabolic process | -0.61 | 5.70E-04 |
| 87 | negative regulation of apoptotic process | 0.61 | 5.94E-04 |
| 88 | DNA double-strand break processing | -0.61 | 5.94E-04 |
| 89 | vitamin metabolic process | -0.61 | 5.94E-04 |
| 90 | cellular metabolic process | -0.61 | 6.06E-04 |
| 91 | COPI coating of Golgi vesicle | 0.61 | 6.19E-04 |
| 92 | negative regulation of protein kinase activity | 0.61 | 6.45E-04 |
| 93 | muscle organ morphogenesis | 0.61 | 6.71E-04 |
| 94 | respiratory chain complex IV assembly | -0.6 | 6.85E-04 |
| 95 | lipid metabolic process | 0.6 | 6.99E-04 |
| 96 | intracellular protein transport | 0.6 | 7.28E-04 |
| 97 | ventricular septum morphogenesis | 0.6 | 7.58E-04 |
| 98 | ethanol catabolic process | -0.6 | 8.06E-04 |
| 99 | response to organic cyclic compound | 0.6 | 8.22E-04 |
| 100 | calcium-mediated signaling | 0.6 | 8.22E-04 |
| 101 | positive regulation of interleukin-1 beta production | 0.6 | 8.39E-04 |
| 102 | telomere maintenance via recombination | -0.6 | 8.56E-04 |
| 103 | protein autoubiquitination | 0.59 | 9.08E-04 |
| 104 | bone mineralization | 0.59 | 9.45E-04 |
| 105 | mitotic metaphase plate congression | -0.59 | 9.84E-04 |
| 106 | toll-like receptor 4 signaling pathway | 0.59 | 9.84E-04 |
| 107 | epithelial cell development | -0.59 | 1.00E-03 |
| 108 | chlorophyll biosynthetic process | -0.59 | 1.00E-03 |
| 109 | photosynthesis | -0.59 | 1.00E-03 |
| 110 | COPII vesicle coating | 0.59 | 1.02E-03 |
| 111 | spindle assembly | -0.59 | 1.06E-03 |
| 112 | non-canonical Wnt signaling pathway | 0.59 | 1.06E-03 |
| 113 | positive regulation of cellular component movement | 0.59 | 1.09E-03 |
| 114 | intracellular signal transduction | 0.59 | 1.13E-03 |
| 115 | positive regulation of catalytic activity | 0.58 | 1.15E-03 |
| 116 | transcription from mitochondrial promoter | -0.58 | 1.18E-03 |
| 117 | Golgi organization | 0.58 | 1.20E-03 |
| 118 | termination of RNA polymerase II transcription | -0.58 | 1.22E-03 |
| 119 | sterol metabolic process | 0.58 | 1.22E-03 |
| 120 | intermediate filament organization | 0.58 | 1.22E-03 |
| 121 | response to growth hormone | -0.58 | 1.25E-03 |
| 122 | outflow tract morphogenesis | 0.58 | 1.30E-03 |
| 123 | ethanol oxidation | -0.58 | 1.32E-03 |
| 124 | gastrulation | 0.58 | 1.35E-03 |
| 125 | neuromuscular process controlling posture | 0.58 | 1.40E-03 |
| 126 | methylation | -0.58 | 1.40E-03 |
| 127 | somatic hypermutation of immunoglobulin genes | -0.58 | 1.40E-03 |
| 128 | skeletal system morphogenesis | 0.58 | 1.46E-03 |
| 129 | ER-associated ubiquitin-dependent protein catabolic process | 0.57 | 1.48E-03 |
| 130 | ER to Golgi vesicle-mediated transport | 0.57 | 1.54E-03 |
| 131 | neuron maturation | 0.57 | 1.54E-03 |
| 132 | negative regulation of mitotic cell cycle | 0.57 | 1.57E-03 |
| 133 | mitotic M phase | -0.57 | 1.63E-03 |
| 134 | RNA modification | -0.57 | 1.63E-03 |
| 135 | DNA metabolic process | -0.57 | 1.67E-03 |
| 136 | cell cycle arrest | 0.57 | 1.73E-03 |
| 137 | activation of MAPKK activity | 0.57 | 1.73E-03 |
| 138 | neuromuscular synaptic transmission | 0.57 | 1.73E-03 |
| 139 | execution phase of apoptosis | 0.57 | 1.80E-03 |
| 140 | folic acid metabolic process | -0.57 | 1.83E-03 |
| 141 | middle ear morphogenesis | 0.57 | 1.87E-03 |
| 142 | phosphatidylinositol biosynthetic process | 0.56 | 1.90E-03 |
| 143 | positive regulation of calcium ion transport into cytosol | 0.56 | 1.98E-03 |
| 144 | negative regulation of wound healing | 0.56 | 2.13E-03 |
| 145 | bundle of His cell to Purkinje myocyte communication | 0.56 | 2.17E-03 |
| 146 | toll-like receptor 9 signaling pathway | 0.56 | 2.21E-03 |
| 147 | neurotrophin TRK receptor signaling pathway | 0.56 | 2.26E-03 |
| 148 | blood vessel development | 0.56 | 2.26E-03 |
| 149 | response to molecule of bacterial origin | 0.56 | 2.26E-03 |
| 150 | angiogenesis | 0.56 | 2.30E-03 |
| 151 | activation of MAPKKK activity | 0.56 | 2.30E-03 |
| 152 | cellular respiration | -0.56 | 2.34E-03 |
| 153 | protein targeting to mitochondrion | -0.56 | 2.34E-03 |
| 154 | negative regulation of phosphorylation | 0.56 | 2.39E-03 |
| 155 | sex determination | 0.56 | 2.39E-03 |
| 156 | nucleotide-excision repair | -0.56 | 2.39E-03 |
| 157 | triglyceride mobilization | 0.56 | 2.39E-03 |
| 158 | UDP-N-acetylglucosamine metabolic process | 0.56 | 2.39E-03 |
| 159 | intrinsic apoptotic signaling pathway in response to endoplasmic reticulum stress | 0.55 | 2.43E-03 |
| 160 | lipid transport | 0.55 | 2.48E-03 |
| 161 | regulation of protein ubiquitination | 0.55 | 2.52E-03 |
| 162 | mismatch repair | -0.55 | 2.67E-03 |
| 163 | 'de novo' IMP biosynthetic process | -0.55 | 2.72E-03 |
| 164 | regulation of MAP kinase activity | 0.55 | 2.72E-03 |
| 165 | regulation of alternative mRNA splicing, via spliceosome | -0.55 | 2.77E-03 |
| 166 | negative regulation of NF-kappaB transcription factor activity | 0.55 | 2.77E-03 |
| 167 | release of sequestered calcium ion into cytosol | 0.55 | 2.77E-03 |
| 168 | protein secretion | 0.55 | 2.77E-03 |
| 169 | brain development | 0.55 | 2.82E-03 |
| 170 | reciprocal meiotic recombination | -0.55 | 2.82E-03 |
| 171 | positive regulation of macroautophagy | 0.55 | 2.82E-03 |
| 172 | proteasome-mediated ubiquitin-dependent protein catabolic process | 0.55 | 2.98E-03 |
| 173 | TRIF-dependent toll-like receptor signaling pathway | 0.55 | 2.98E-03 |
| 174 | mRNA transport | -0.55 | 3.04E-03 |
| 175 | positive regulation of canonical Wnt signaling pathway | 0.54 | 3.09E-03 |
| 176 | ubiquitin-dependent protein catabolic process | -0.54 | 3.15E-03 |
| 177 | positive regulation of exocytosis | 0.54 | 3.15E-03 |
| 178 | negative regulation of DNA damage response, signal transduction by p53 class mediator | 0.54 | 3.21E-03 |
| 179 | cellular response to hormone stimulus | 0.54 | 3.27E-03 |
| 180 | somatic recombination of immunoglobulin gene segments | -0.54 | 3.27E-03 |
| 181 | response to vitamin E | 0.54 | 3.27E-03 |
| 182 | positive regulation of membrane protein ectodomain proteolysis | -0.54 | 3.33E-03 |
| 183 | cellular response to hydrogen peroxide | 0.54 | 3.33E-03 |
| 184 | peptidyl-proline hydroxylation to 4-hydroxy-L-proline | 0.54 | 3.39E-03 |
| 185 | post-embryonic development | 0.54 | 3.45E-03 |
| 186 | long-chain fatty-acyl-CoA biosynthetic process | 0.54 | 3.51E-03 |
| 187 | spliceosomal snRNP assembly | -0.54 | 3.51E-03 |
| 188 | ion transmembrane transport | 0.54 | 3.51E-03 |
| 189 | ER overload response | 0.54 | 3.51E-03 |
| 190 | lipid biosynthetic process | 0.54 | 3.58E-03 |
| 191 | apical junction assembly | 0.54 | 3.58E-03 |
| 192 | positive regulation of protein kinase activity | 0.54 | 3.71E-03 |
| 193 | lung alveolus development | 0.54 | 3.71E-03 |
| 194 | striatum development | -0.54 | 3.71E-03 |
| 195 | negative regulation of protein phosphorylation | 0.54 | 3.78E-03 |
| 196 | MyD88-independent toll-like receptor signaling pathway | 0.54 | 3.78E-03 |
| 197 | transforming growth factor beta receptor signaling pathway | 0.54 | 3.84E-03 |
| 198 | cellular protein localization | 0.54 | 3.84E-03 |
| 199 | release of cytochrome c from mitochondria | 0.54 | 3.84E-03 |
| 200 | Mo-molybdopterin cofactor biosynthetic process | -0.54 | 3.84E-03 |
| 201 | leukocyte migration involved in inflammatory response | -0.54 | 3.84E-03 |
| 202 | response to light stimulus | 0.53 | 3.91E-03 |
| 203 | cellular response to glucose starvation | 0.53 | 3.99E-03 |
| 204 | cellular response to hypoxia | 0.53 | 4.13E-03 |
| 205 | palate development | 0.53 | 4.13E-03 |
| 206 | positive regulation of insulin secretion | 0.53 | 4.13E-03 |
| 207 | tricarboxylic acid cycle | -0.53 | 4.21E-03 |
| 208 | positive regulation of MAPK cascade | 0.53 | 4.21E-03 |
| 209 | multicellular organism growth | 0.53 | 4.28E-03 |
| 210 | nucleobase-containing compound metabolic process | -0.53 | 4.28E-03 |
| 211 | ncRNA metabolic process | -0.53 | 4.28E-03 |
| 212 | intermediate filament cytoskeleton organization | 0.53 | 4.28E-03 |
| 213 | oligosaccharide catabolic process | 0.53 | 4.52E-03 |
| 214 | cell migration | 0.53 | 4.60E-03 |
| 215 | organ regeneration | -0.53 | 4.60E-03 |
| 216 | cellular response to peptide | 0.53 | 4.60E-03 |
| 217 | N-acetylglucosamine metabolic process | 0.53 | 4.68E-03 |
| 218 | sex differentiation | 0.53 | 4.85E-03 |
| 219 | lens fiber cell differentiation | 0.53 | 4.85E-03 |
| 220 | regulation of autophagy | 0.52 | 4.93E-03 |
| 221 | hemidesmosome assembly | 0.52 | 4.93E-03 |
| 222 | protein K6-linked ubiquitination | -0.52 | 4.93E-03 |
| 223 | phosphatidylethanolamine biosynthetic process | 0.52 | 5.02E-03 |
| 224 | negative regulation of microtubule polymerization | -0.52 | 5.02E-03 |
| 225 | cell junction assembly | 0.52 | 5.20E-03 |
| 226 | apoptotic signaling pathway | 0.52 | 5.29E-03 |
| 227 | embryonic cranial skeleton morphogenesis | 0.52 | 5.29E-03 |
| 228 | response to sucrose | 0.52 | 5.29E-03 |
| 229 | chondrocyte development | 0.52 | 5.29E-03 |
| 230 | chaperone-mediated protein transport | -0.52 | 5.29E-03 |
| 231 | cholesterol metabolic process | 0.52 | 5.58E-03 |
| 232 | retinal metabolic process | 0.52 | 5.58E-03 |
| 233 | protein glycosylation | 0.52 | 5.68E-03 |
| 234 | sphingolipid biosynthetic process | 0.52 | 5.68E-03 |
| 235 | autophagic vacuole assembly | 0.52 | 5.78E-03 |
| 236 | response to unfolded protein | 0.52 | 5.88E-03 |
| 237 | negative regulation of DNA recombination | -0.52 | 5.98E-03 |
| 238 | negative regulation of cell cycle | 0.52 | 5.98E-03 |
| 239 | cellular response to insulin stimulus | 0.52 | 6.08E-03 |
| 240 | tissue remodeling | 0.52 | 6.08E-03 |
| 241 | mitochondrion organization | -0.51 | 6.19E-03 |
| 242 | negative regulation of DNA replication | -0.51 | 6.19E-03 |
| 243 | pyrimidine nucleobase catabolic process | 0.51 | 6.19E-03 |
| 244 | cotranslational protein targeting to membrane | 0.51 | 6.30E-03 |
| 245 | positive regulation of helicase activity | -0.51 | 6.30E-03 |
| 246 | positive regulation of histone H3-K4 methylation | -0.51 | 6.41E-03 |
| 247 | histone lysine methylation | -0.51 | 6.41E-03 |
| 248 | lipoprotein biosynthetic process | -0.51 | 6.41E-03 |
| 249 | long-chain fatty acid transport | 0.51 | 6.41E-03 |
| 250 | purinergic nucleotide receptor signaling pathway | 0.51 | 6.41E-03 |
| 251 | chondrocyte differentiation | 0.51 | 6.52E-03 |
| 252 | chromatin modification | -0.51 | 6.63E-03 |
| 253 | cellular response to follicle-stimulating hormone stimulus | 0.51 | 6.63E-03 |
| 254 | negative regulation of T cell receptor signaling pathway | 0.51 | 6.63E-03 |
| 255 | post-translational protein modification | 0.51 | 6.75E-03 |
| 256 | skeletal system development | 0.51 | 6.75E-03 |
| 257 | maturation of 5.8S rRNA | -0.51 | 6.75E-03 |
| 258 | biological process | -0.51 | 6.87E-03 |
| 259 | cellular response to growth factor stimulus | 0.51 | 6.87E-03 |
| 260 | hematopoietic progenitor cell differentiation | 0.51 | 6.98E-03 |
| 261 | mitotic cell cycle arrest | 0.51 | 6.98E-03 |
| 262 | spermatid nucleus differentiation | 0.51 | 6.98E-03 |
| 263 | positive regulation of cell migration | 0.51 | 7.10E-03 |
| 264 | arginine metabolic process | 0.51 | 7.10E-03 |
| 265 | erythrocyte homeostasis | 0.51 | 7.10E-03 |
| 266 | positive regulation of bone mineralization | 0.51 | 7.23E-03 |
| 267 | ribonucleoside monophosphate biosynthetic process | -0.51 | 7.23E-03 |
| 268 | peripheral nervous system development | 0.51 | 7.35E-03 |
| 269 | anaphase-promoting complex-dependent proteasomal ubiquitin-dependent protein catabolic process | -0.51 | 7.35E-03 |
| 270 | axon guidance | 0.51 | 7.35E-03 |
| 271 | cellular response to extracellular stimulus | 0.51 | 7.35E-03 |
| 272 | regulation of fat cell differentiation | 0.51 | 7.35E-03 |
| 273 | protein retention in ER lumen | 0.51 | 7.48E-03 |
| 274 | negative regulation of glucose import | 0.51 | 7.48E-03 |
| 275 | water-soluble vitamin metabolic process | -0.5 | 7.74E-03 |
| 276 | response to mechanical stimulus | 0.5 | 7.87E-03 |
| 277 | response to hyperoxia | 0.5 | 7.87E-03 |
| 278 | common-partner SMAD protein phosphorylation | 0.5 | 8.00E-03 |
| 279 | inflammatory response | 0.5 | 8.14E-03 |
| 280 | cellular iron ion homeostasis | 0.5 | 8.14E-03 |
| 281 | branching involved in mammary gland duct morphogenesis | 0.5 | 8.14E-03 |
| 282 | positive regulation of T cell mediated cytotoxicity | 0.5 | 8.14E-03 |
| 283 | double-strand break repair via homologous recombination | -0.5 | 8.28E-03 |
| 284 | positive regulation of nitric oxide biosynthetic process | 0.5 | 8.28E-03 |
| 285 | regulation of angiogenesis | 0.5 | 8.42E-03 |
| 286 | negative regulation of ubiquitin-protein ligase activity involved in mitotic cell cycle | -0.5 | 8.56E-03 |
| 287 | ossification | 0.5 | 8.56E-03 |
| 288 | type I interferon signaling pathway | 0.5 | 8.56E-03 |
| 289 | cellular protein metabolic process | 0.5 | 8.71E-03 |
| 290 | protein polymerization | -0.5 | 8.71E-03 |
| 291 | positive regulation of phosphorylation | 0.5 | 8.85E-03 |
| 292 | regulation of organ growth | 0.5 | 8.85E-03 |
| 293 | nucleobase-containing small molecule metabolic process | -0.5 | 8.85E-03 |
| 294 | positive regulation of interleukin-17 production | 0.5 | 8.85E-03 |
| 295 | response to fatty acid | 0.5 | 9.00E-03 |
| 296 | branched-chain amino acid catabolic process | -0.5 | 9.00E-03 |
| 297 | response to gonadotropin | 0.5 | 9.00E-03 |
| 298 | negative regulation of adenylate cyclase activity | 0.5 | 9.15E-03 |
| 299 | pantothenate metabolic process | -0.5 | 9.15E-03 |
| 300 | postreplication repair | -0.5 | 9.15E-03 |
| 301 | arachidonic acid secretion | 0.5 | 9.15E-03 |
| 302 | triglyceride metabolic process | 0.5 | 9.31E-03 |
| 303 | endocardial cushion development | 0.5 | 9.31E-03 |
| 304 | positive regulation of protein homooligomerization | 0.5 | 9.31E-03 |
| 305 | commissural neuron axon guidance | 0.5 | 9.31E-03 |
| 306 | relaxation of cardiac muscle | 0.5 | 9.31E-03 |
| 307 | activation of cysteine-type endopeptidase activity involved in apoptotic process by cytochrome c | 0.5 | 9.31E-03 |
| 308 | regulation of ubiquitin-protein ligase activity involved in mitotic cell cycle | -0.5 | 9.46E-03 |
| 309 | positive regulation of peptidyl-threonine phosphorylation | 0.5 | 9.46E-03 |
| 310 | histone mRNA metabolic process | -0.5 | 9.46E-03 |
| 311 | lipid storage | 0.49 | 9.62E-03 |
| 312 | positive regulation of leukocyte migration | 0.49 | 9.62E-03 |
| 313 | negative regulation of apoptotic signaling pathway | 0.49 | 9.62E-03 |
| 314 | negative regulation of JUN kinase activity | 0.49 | 9.78E-03 |
| 315 | centrosome cycle | -0.49 | 9.78E-03 |
| 316 | positive regulation of interferon-beta production | 0.49 | 1.01E-02 |
| 317 | ventricular system development | 0.49 | 1.01E-02 |
| 318 | cellular response to fatty acid | -0.49 | 1.01E-02 |
| 319 | heme biosynthetic process | -0.49 | 1.03E-02 |
| 320 | histone H3 deacetylation | 0.49 | 1.03E-02 |
| 321 | glomerular visceral epithelial cell development | 0.49 | 1.03E-02 |
| 322 | positive regulation of epithelial cell proliferation | 0.49 | 1.05E-02 |
| 323 | peptidyl-threonine dephosphorylation | 0.49 | 1.05E-02 |
| 324 | lung development | 0.49 | 1.06E-02 |
| 325 | regulation of JNK cascade | 0.49 | 1.06E-02 |
| 326 | histone mRNA catabolic process | -0.49 | 1.06E-02 |
| 327 | methionine biosynthetic process | -0.49 | 1.06E-02 |

Table S5. Sensitivity of 42 glioma cells to TMZ and 11 candidates.

| ***treatment*** | ***BRD-K32107296-001-16-9***  ***temozolomide*** | ***BRD-K63165456-001-10-8***  ***norcyclobenzaprine*** | ***BRD-K42098891-003-20-5***  ***protriptyline*** | ***BRD-K43860855-065-11-9***  ***iobenguane*** | ***BRD-K67783091-001-26-1***  ***haloperidol*** | ***BRD-A49225603-045-10-0***  ***alimemazine*** | ***BRD-K91263825-003-21-4***  ***nortriptyline*** | ***BRD-K97530723-001-20-9***  ***melatonin*** | ***BRD-K89732114-300-14-7***  ***trifluoperazine*** | ***BRD-K10995081-001-27-9***  ***perphenazine*** | ***BRD-K55468218-001-26-1***  ***spiperone*** | ***BRD-K38436528-003-26-1***  ***imipramine*** | ***BRD-K35559145-050-08-5***  ***levomepromazine*** |
| --- | --- | --- | --- | --- | --- | --- | --- | --- | --- | --- | --- | --- | --- |
| ***cell line*** |  |  |  |  |  |  |  |  |  |  |  |  |  |
| GOS3 | -0.944 | 0.538 | 0.321 | 0.078 | 0.110 | 0.020 | -0.191 | -1.078 | 0.249 | -0.045 | -0.249 | -0.067 | 0.216 |
| U118MG* | 0.349 | -1.195 |  | 0.911 | -0.718 | 0.725 | 0.544 | 0.952 | -0.737 | 0.263 | -0.182 |  | -0.473 |
| U87MG | 0.111 | 0.807 | 0.898 | 0.073 | -0.151 | 0.117 | 0.145 | -1.269 | -0.224 | -0.269 | -0.476 | 0.138 | 0.414 |
| GAMG | 0.095 | 0.176 | 0.193 | -0.057 | 0.035 | 0.026 | 0.525 | 0.411 | 0.454 | 0.010 | -0.383 | -0.261 | -0.161 |
| GMS10 | -0.260 | 0.287 | 0.380 | 0.578 | 0.178 | -0.079 | 0.657 | -0.189 | -0.432 | 0.170 | -0.009 | 0.024 | -0.003 |
| 8MGBA | -0.978 | -0.343 | 0.121 | 0.740 | 0.049 | 0.691 | -1.187 | 0.639 | -1.049 | 0.023 | 0.547 | -1.392 | 0.261 |
| NMCG1 | -0.297 | 0.345 | -0.058 | 0.407 | -0.063 | -0.707 | 0.496 | -1.087 | 0.315 | 0.004 | 0.522 | 0.294 | -1.322 |
| DAOY | -0.057 | 0.063 | 0.189 | 0.051 | 0.458 | -0.038 | 0.281 | -0.180 | -0.214 | 0.190 | 0.330 | -0.170 | -0.074 |
| KALS1 | -0.301 | -0.069 | -0.113 | 0.770 | -0.043 | 0.329 | -0.236 | -1.142 | 0.140 | -0.043 | 0.210 | -0.526 | 0.310 |
| U251MG | -0.455 | 0.377 | 0.399 | 0.356 | 0.272 | 0.488 | 0.237 | 0.114 | 0.118 | 0.175 | 0.296 | 0.269 | 0.129 |
| DKMG | -0.497 | 0.504 | 0.043 | 0.155 | -0.516 | -0.451 | -0.442 | -0.392 | 0.310 | -0.096 | 0.253 | 0.122 | -0.533 |
| AM38* |  |  |  | 1.587 |  | -0.840 |  | 0.506 |  | -0.109 |  |  | -0.532 |
| SF539 | -0.974 | 0.173 | 0.109 | -0.686 | -0.004 | 0.283 | 0.079 | 0.019 | -0.189 | -0.065 | -0.103 | -0.595 | 0.215 |
| SNU466 | 0.272 | 0.189 | 0.025 | -0.126 | 0.125 | -0.210 | -0.343 | -0.292 | 0.077 | 0.669 | -0.640 | 0.681 | -0.727 |
| 42MGBA | -0.469 | -0.103 | 0.442 | 0.201 | 0.271 | 0.212 | -0.123 | 0.191 | -0.022 | 0.139 | -0.137 | -0.007 | -0.032 |
| CCFSTTG1 | -0.546 | 0.542 | 0.075 | -0.224 | 0.351 | 0.243 | 0.312 | 0.672 | -0.035 | -0.418 | 0.039 | 0.162 | 0.000 |
| SNU1105 | -0.020 | 0.339 | -0.037 | -0.310 | 0.245 | 0.169 | -0.092 | 0.388 | -0.036 | 0.008 | -0.176 | 0.323 | -0.078 |
| SF295 | 0.523 | 0.093 | -0.191 | -0.520 | -0.112 | -0.217 | 0.734 | 0.515 | 0.973 | 0.113 | -0.108 | 0.223 | 0.611 |
| H4 | -0.387 | 0.484 | 0.691 | 0.756 | 0.045 | 0.013 | -0.131 | -0.050 | 0.396 | 0.003 | 0.097 | -0.274 | 0.048 |
| SW1088 | 0.086 | 0.186 | 0.487 | 0.442 | 0.609 | 0.062 | 0.070 | -0.194 | -0.032 | 0.068 | -0.346 | -0.216 | 0.025 |
| KNS60 | -0.127 | 0.330 | 0.278 | -0.024 | -0.028 | 0.207 | 0.259 | -0.054 | 0.015 | 0.129 | -0.167 | -0.267 | -0.006 |
| CAS1 | -0.071 | 0.603 | 0.268 | -0.215 | -0.109 | -0.270 | 0.010 | 0.154 | -0.523 | 0.012 | -0.327 | 0.256 | -0.201 |
| YH13 | 0.302 | -0.115 | -0.780 | -0.145 | 0.268 | -0.170 | 0.232 | -1.006 | -0.105 | -0.265 | 0.578 | 0.216 | 0.185 |
| KNS81 | 0.310 | 0.023 | 0.350 | 0.141 | 0.271 | 0.080 | -0.264 | 0.312 | -0.674 | -0.052 | 0.033 | 0.004 | 0.264 |
| SNB75 | -0.421 | -0.156 | -0.059 | 0.014 | 0.454 | 0.459 | -0.297 | -0.182 | -0.158 | 0.032 | 0.283 | -0.416 | -0.396 |
| SNU489 | -0.011 | 1.260 | 0.769 | 0.245 | -0.107 | -0.382 | 0.359 | 1.550 | 0.587 | 0.212 | 0.882 | -1.136 | 0.129 |
| A172 | 0.326 | 0.707 | 0.108 | -0.043 | 0.262 | 0.305 | -0.257 | 0.343 | 0.059 | 0.072 | -0.001 | 0.290 | 0.358 |
| YKG1 | 0.336 | -0.071 | -0.200 | 0.058 | 0.270 | 0.276 | 0.740 | 0.252 | 0.273 | -0.759 | -0.361 | -0.796 | 0.196 |
| T98G | 0.055 | -0.016 | -0.078 | -0.010 | 0.058 | -0.183 | -0.093 | -0.261 | -0.053 | -0.320 | 0.427 | 0.046 | -0.288 |
| TM31 | -0.057 | 0.095 | 0.891 | 0.049 | 0.659 | -0.328 | -0.427 | 0.281 | 0.006 | -0.347 | 0.485 | -0.031 | 0.332 |
| LN229 | -0.490 | 0.179 | 0.350 | -0.167 | 0.052 | 0.460 | 0.158 | 0.446 | 0.275 | -0.708 | 0.449 | 0.087 | -0.301 |
| SF126 | -0.453 | -0.039 | 0.185 | 0.133 | 0.369 | 0.354 | 0.360 | -0.284 | 0.083 | 0.078 | -0.402 | 0.131 | -0.196 |
| KNS42 | -0.152 | 0.196 | 0.044 | -0.126 | 0.263 | -0.077 | -0.157 | -0.296 | -0.109 | -0.023 | -0.207 | -0.164 | 0.034 |
| SNU201 | -0.596 | 0.641 | -0.327 | -0.009 | -0.022 | -0.271 | -0.710 | 0.686 | -0.100 | -0.411 | -0.761 | -0.136 | -0.284 |
| KS1* | 0.259 |  |  |  | -0.462 | 0.176 | 0.287 | 0.398 | 0.254 |  | -0.394 |  | -0.061 |
| GB1 | 0.443 | 0.422 | 0.115 | 0.136 | -0.208 | -0.472 | 0.469 | -0.542 | -0.021 | 0.189 | 0.050 | 0.030 | 0.254 |
| GI1 | -1.633 | -0.662 | 0.013 | -0.216 | 0.096 | -0.283 | -0.421 | -0.293 | -0.161 | -0.551 | -0.560 | 0.011 | -0.442 |
| ONS76 | -0.136 | 0.110 | 0.500 | -0.325 | 0.273 | 0.130 | -0.010 | 0.237 | -0.162 | 0.091 | -0.389 | 0.343 | 0.123 |
| LN18* | 0.098 | 0.294 | 0.083 | 0.181 | 0.356 | 0.174 | 0.203 | 0.109 | 0.367 |  | -0.020 | 0.114 | -0.136 |
| DBTRG05MG | -0.068 | 0.489 | 0.144 | 0.297 | 0.076 | 0.301 | -0.169 | 0.048 | -0.204 | 0.456 | -0.070 | -0.010 | -0.659 |
| SW1783 | -1.142 | -0.156 | 0.249 | -0.634 | 0.175 | 0.383 | -0.137 | 0.197 | -0.144 | 0.376 | -1.027 | -0.146 | -0.696 |
| BECKER | -0.081 | 0.495 | 0.142 | 0.107 | 0.374 | -0.070 | -0.238 | -0.207 | 0.280 | -0.183 | -0.107 | -0.067 | 0.119 |
| mean | -0.197 | 0.200 | 0.180 | 0.113 | 0.109 | 0.039 | 0.030 | 0.010 | -0.004 | -0.029 | -0.052 | -0.075 | -0.080 |
| 95%CI | -0.340 | 0.073 | 0.078 | -0.017 | 0.024 | -0.065 | -0.092 | -0.165 | -0.115 | -0.119 | -0.174 | -0.200 | -0.194 |
|  | -0.053 | 0.328 | 0.282 | 0.243 | 0.195 | 0.143 | 0.152 | 0.185 | 0.107 | 0.060 | 0.071 | 0.050 | 0.033 |
| P-value | Ref | 5.89E-05 | 3.84E-05 | 1.23E-03 | 3.16E-04 | 5.61E-03 | 1.05E-02 | 3.86E-02 | 2.03E-02 | 2.84E-02 | 6.81E-02 | 1.06E-01 | 1.08E-01 |
| Count | 41 | 40 | 39 | 41 | 41 | 42 | 41 | 42 | 41 | 40 | 41 | 39 | 42 |
| SD | 0.469 | 0.411 | 0.324 | 0.426 | 0.279 | 0.345 | 0.400 | 0.578 | 0.363 | 0.288 | 0.400 | 0.398 | 0.375 |
| * Cell line LN18, KS1, AM38 and U118MG lack part of the sensitivity data. | | | | | | | | | | | | | |

Table S6. Target prediction of candidate drugs

| ***Norcyclobenzaprine*** | | | | |
| --- | --- | --- | --- | --- |
| ***Entry num.*** | ***Gene ID*** | ***Uniprot ID*** | ***PDB ID*** | ***Score*** |
| 1 | PARP1 | P09874 | 5ws0 | 0.809 |
| 2 | PARP2 | Q9UGN5 | 4pjv | 0.746 |
| 3 | ORM2 | P19652 | 3apv | 0.675 |
| 4 | RBP1 | P09455 | 6e6m | 0.634 |
| 5 | SETD7 | Q8WTS6 | 5ayf | 0.632 |
| 6 | ESRRG | P62508 | 2gpp | 0.629 |
| 7 | PGR | P06401,P06401 | 1a28 | 0.593 |
| 8 | ESR1 | P03372 | 5dlr | 0.59 |
| 9 | NCOA2 | O00482,Q15596 | 6or1 | 0.556 |
| 10 | RARB | P10826 | 4jyi | 0.548 |
| ***Protriptyline*** | | | | |
| ***Entry num.*** | ***Gene ID*** | ***Uniprot ID*** | ***PDB ID*** | ***Score*** |
| 1 | RBP1 | P09455 | 6e5t | 0.784 |
| 2 | PGR | P06401,P06401 | 1a28 | 0.767 |
| 3 | NCOA2 | O00482,Q15596 | 6or1 | 0.681 |
| 4 | PARP2 | Q9UGN5 | 4pjv | 0.671 |
| 5 | PARP1 | P09874 | 4r6e | 0.662 |
| 6 | RXRA | P19793 | 6a5y | 0.657 |
| 7 | RARB | P10826 | 4jyi | 0.622 |
| 8 | CHKA | P35790 | 5fut | 0.597 |
| 9 | RARG | P13631 | 1fd0 | 0.594 |
| 10 | ORM2 | P19652 | 3apv | 0.562 |
